# Best Oculomotor Endpoints for Clinical Trials in Hereditary Ataxias: A Systematic Review and Consensus by the Ataxia Global Initiative Working Group on Digital‑Motor Biomarkers

**DOI:** 10.1007/s12311-025-01894-z

**Published:** 2025-08-13

**Authors:** Elena Pretegiani, Pilar Garces, Chrystalina A. Antoniades, Anna Sobanska, Norbert Kovacs, Sarah H. Ying, Anoopum S. Gupta, Susan Perlman, David J. Szmulewicz, Chiara Pane, Andrea H. Németh, Laura B. Jardim, Giulia Coarelli, Michaela Kuzmiak, Andona Milovanovic, Andreas Traschütz, Alexander A. Tarnutzer

**Affiliations:** 1 Unit of Neurology, Centre Hospitalier Universitaire Vaudoise Lausanne, Lausanne, Switzerland; 2 Roche Pharma Research and Early Development, Neuroscience and Rare Diseases, Roche Innovation Center Basel, Basel, Switzerland; 3 NeuroMetrology Lab, Nuffield Department of Clinical Neurosciences, Clinical Neurology, Medical Sciences Division, University of Oxford, Oxford, OX3 9DU UK; 4 Institute of Psychiatry and Neurology, Warsaw, Poland; 5 Department of Neurology, Medical School, University of Pécs, Pécs, Hungary; 6 Department of Otology and Laryngology and Department of Neurology, Harvard Medical School, Boston, MA USA; 7 Department of Neurology, Massachusetts General Hospital, Harvard Medical School, Boston, MA USA; 8 University of California Los Angeles, Los Angeles, CA USA; 9 Balance Disorders and Ataxia Service, Royal Victoria Eye and Ear Hospital, East Melbourne, Melbourne, VIC 3002 Australia; 10 The Bionics Institute, East Melbourne, Melbourne, VIC 3002 Australia; 11 Department of Neurosciences and Reproductive and Odontostomatological Sciences, University of Naples “Federico II”, Naples, Italy; 12 Nuffield Department of Clinical Neurosciences, University of Oxford, Oxford, UK; 13 Oxford Centre for Genomic Medicine, Oxford University Hospitals NHS Trust, Oxford, UK; 14 Departamento de Medicina Interna, Universidade Federal Do Rio Grande Do Sul, Porto Alegre, Brazil; 15 Serviço de Genética Médica/Centro de Pesquisa Clínica E Experimental, Hospital de Clínicas de Porto Alegre, Porto Alegre, Brazil; 16 APHPSorbonne Université, Paris Brain Institute, Inserm, CNRS, Pitié-Salpêtrière Hospital, DMU Biogem, APHP, 75013 Paris, France; 17 Department of Neurology, 2nd Faculty of Medicine, Centre of Hereditary Ataxias, Charles University and Motol University Hospital, Prague, Czech Republic; 18 Clinic for Neurology, University Clinical Center of Serbia, Belgrade, Serbia; 19 Hertie-Institute for Clinical Brain Research and Center of Neurology, Division Translational Genomics of Neurodegenerative Diseases, University of Tübingen, Tübingen, Germany; 20 German Center for Neurodegenerative Diseases (DZNE), University of Tübingen, Tübingen, Germany; 21 Cantonal Hospital of Baden, Baden, Switzerland; 22 Faculty of Medicine, University of Zurich, Zurich, Switzerland

**Keywords:** Oculomotor, Vestibular, Eye movement recordings, Hereditary ataxia, Systematic review, Recommendations

## Abstract

Oculomotor deficits are common in hereditary cerebellar ataxias (HCAs) and their quantitative assessment offers a sensitive and reliable manner to capture disease-severity and progression. As a group of experts of the Ataxia Global Initiative to support trial readiness, we previously established harmonized methodology for quantitative oculomotor assessments in HCAs. Here, we aimed to identify to most promising oculomotor/vestibular outcomes as endpoints for future trials. Through a systematic MEDLINE search we identified 130 articles reporting oculomotor/vestibular recordings in patients with HCAs. A total of 2,018 subjects were included: 1,776 with genetically-confirmed and 242 with clinically-defined HCAs. Studied diseases included spinocerebellar ataxias (SCA) 1/2/3/6/7/27B, episodic ataxia type 2, Friedreich ataxia, RFC1-related ataxia, fragile X-associated tremor/ataxia syndrome, cerebrotendinous xanthomatosis, ataxia-telangiectasia, ataxia with oculomotor apraxia types 1&2, and Niemann-Pick disease type C. We identified up to four oculomotor/vestibular outcomes per diagnostic entity, based on their ability to robustly discriminate patients from controls, correlate with disease-severity, detect longitudinal change, and represent different disease stages. For each parameter we provide recommendations for recordings. While the implementation of quantitative assessments into clinical trials offers a unique opportunity to track dysfunction of oculomotor/vestibular networks and to assess the impact of interventions, in some HCAs, endpoint qualification of available outcomes requires further validation to characterize their reliability, sensitivity to change, and minimally important change to patients. For all HCAs for which quantitative data are scarce or lacking, there is an urgent need for prospective studies covering a broader range of oculomotor/vestibular domains as approaching new treatments require harmonized and reliable endpoints.

**Supplementary Information:**

The online version contains supplementary material available at 10.1007/s12311-025-01894-z.

## Introduction

Hereditary cerebellar ataxias (HCAs) are frequently associated with characteristic oculomotor abnormalities. These oculomotor changes have proven to be sensitive and reliable tools for assessing disease progression and evaluating therapeutic responses. Despite their clinical utility, bedside eye movement assessments are often omitted or only superficially considered in commonly used clinical scales, such as the Scale for the Assessment and Rating of Ataxia (SARA) [1] or the International Ataxia Cooperative Rating Scale (ICARS) [2]. Addressing this issue, recently, a clinical scale reporting on ocular disorders in ataxia (SODA) has been developed, but its application remains limited and not immune to subjectivity [3].

Eye movements can also be recorded quantitatively by increasingly available, portable and easy to use eye-tracking technologies offering significant advantages over clinical examination: reproducibility, objectivity, and increased sensitivity to abnormalities and subtle changes [4]. However, among clinicians and researchers, the knowledge of which parameters are more informative for every specific HCA is still scarce. This is particularly relevant as approaching treatments in HCAs have created a need for the identification of reliable trial endpoints: a central objective of the Ataxia Global Initiative (AGI).

Indeed, the pharmacological landscape for HCAs is rapidly evolving with a combination of emerging symptomatic therapies and a significant pipeline of disease modifying therapies, with the recent U.S. Food and Drug Administration (FDA) approval of omaveloxolone for Friedreich ataxia (FRDA) marking a milestone in disease-modifying treatments. This progress underscores the pressure for robust, objective and measurable biomarkers to support multicentric studies as demanded by regulators such as the FDA.

According to the FDA-NIH biomarker working group, an endpoint is a “defined parameter intended to reflect an outcome of interest that is statistically analyzed to address a particular research question” [5]. To be ideal endpoints, biomarkers should be meaningful to patients, demonstrate proven or likely sensitivity to small changes in a timeframe of a maximum of 1–2 years, with low variability even in a minimal number of patients, and comprehensively address all aspects of a trial scenario (AGI). Moreover, when a parameter is identified as endpoint, indications on the modality and timing of assessments should be provided. In this regard, quantitative oculomotor metrics stand out as potential trial endpoints due to their precision, sensitivity, and non-invasive nature.

As part of the AGI, we have previously reviewed the patterns of oculomotor and vestibular abnormalities in distinct HCAs, and how they relate to other measures of disease severity, also providing consensus recommendations and technical guidelines for best oculomotor and vestibular recordings [4, 6].

In this paper, continuing to adhere to the AGI effort for identifying optimal trial endpoints, we provide an evidence-based analysis of selected quantitative oculomotor parameters for specific HCAs along with consensus suggestions on their optimal measurement and application. We aim to guide the design of clinical trials requiring ataxia endpoints by presenting a curated list of validated quantitative oculomotor and vestibular outcomes for the most prevalent HCAs. Furthermore, we address existing gaps in the identification of these endpoints and the current efforts and future needs to close these gaps.

## Material and Methods

### Data Sources and Searches

We searched MEDLINE (via PubMed) for articles using text words and controlled-vocabulary terms related to research studies reporting on quantitative assessment of oculomotor and/or vestibular functions in HCA. A detailed description of the search strategy can be found in Appendix 1. Our search was updated through May 7th 2024**.**

### Study Selection and Quality Rating

Articles were selected by two independent raters (PG and AAT) using pre-determined inclusion criteria and a structured protocol (see Appendix 1). Our focus was on studies reporting on *quantitative* oculomotor and/or vestibular testing in the most prevalent HCAs. To identify relevant HCAs we followed the nomenclature of autosomal dominant [7] and autosomal recessive [8, 9] ataxias, to those we added cerebellar ataxia neuropathy vestibular areflexia syndrome (CANVAS), Niemann-Pick type syndrome (NPC), and SCA27B.

**Fig. 1:**
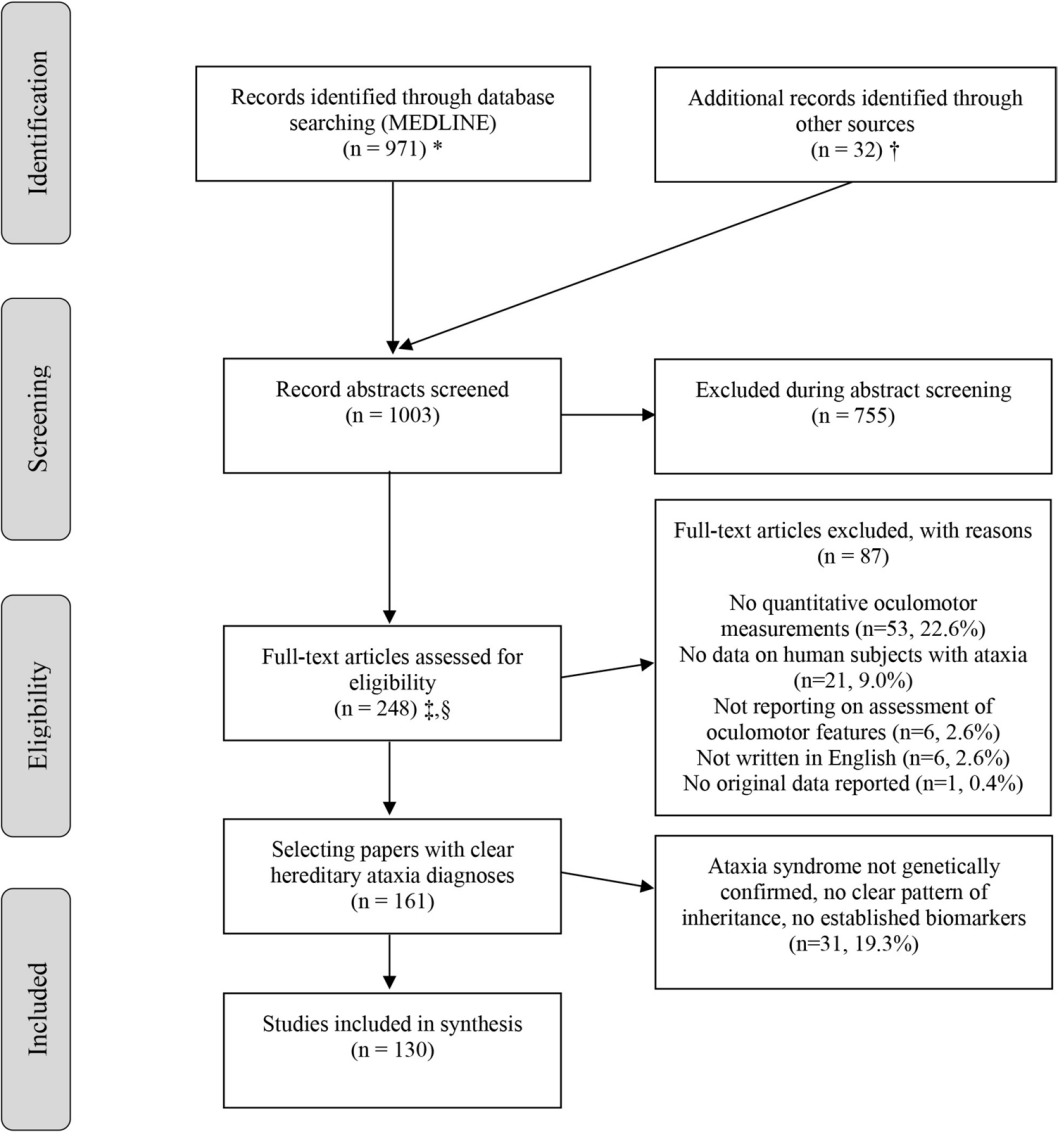
* MEDLINE was accessed via PubMed. † Individual hand search of citation lists from selected studies and investigator files identified 17 additional manuscripts for review. ‡ Abstracts coded as “yes” or “maybe” by at least one reviewer were included in full-text review. § After full-text evaluation by two reviewers, any differences were resolved by discussion and – if needed—adjudication by a third, independent reviewer

For this review we included only studies with well-defined patient cohorts: a) with genetically confirmed HCA or b) (if no genetic testing was available) ataxias with either a positive family history with a clear pattern of inheritance (autosomal dominant, autosomal recessive, X-linked recessive) or c) with established and specific diagnostic biomarkers that allowed to clinically confirm an inherited ataxia (as e.g. Alpha-fetoprotein (AFP) in ataxia telangiectasia and ataxia with ocular motor apraxia 2 [10]), as illustrated in the PRISMA flow-chart (Fig. 1). We calculated inter-rater agreement on full-text inclusion using Cohen’s kappa.

A quality rating of included studies was performed based on eight predefined quality criteria covering items related to (i) the study-cohort, (ii) data acquisition and (iii) data analysis (see Appendix 2 and our previous publication [4, 6]). An overall study rating (high, moderate, low) was derived from this assessment.

### Data Extraction, Data Synthesis and Statistical Analysis

From all eligible articles included in this meta-analysis, we extracted information regarding the study type and its sample size, the disease cohort, the oculomotor paradigms applied and the eye movement recording device(s) used. We also searched for correlations between oculomotor parameters and anchor measures of disease disability such as clinical parameters (clinical scales, various questionnaires, disease duration, age of onset), biological determinants (e.g., CAG repeat length in polyQ diseases), and/or imaging markers. The whole study was performed in accordance with PRISMA guidelines [11].

From each publication, we retrieved key information on the type of oculomotor paradigms performed and rated their potential responsiveness as oculomotor endpoints. In view of the proposed criteria for ideal endpoints [12], we defined a set of criteria for potential oculomotor endpoints, taking into account the number of studies/patients that investigated a given parameter including study design and quality, the magnitude of change of the parameter identified, and any correlations with other established (non-oculomotor) parameters. Specifically, we assessed whether oculomotor parameters: (i) identified any abnormalities in the patient group, (ii) discriminated significantly from healthy control groups, (iii) correlated to anchor measures of disease severity, (iv) captured progression over time (longitudinal observational studies), (v) captured modulation of progression by an intervention (clinical treatment trials), and (vi) included asymptomatic carriers and/or early disease stages (i.e., disease duration < 2 years). Correlations with anchor measures were classified for studies reporting Pearson correlation coefficients or Spearman correlation coefficients (strong: r ≥ 0.7; moderate: r ≥ 0.4 and r < 0.7; weak: r ≥ 0.1 and r < 0.4) [13].

In order to evaluate the potential utility of oculomotor parameters in HCA patients, we devised a scoring system (range: 0–7 points), providing 1 point each for oculomotor measure that met criteria i, ii, iv, v, and vi as listed above. For any correlation analyses (criterium iii), one point was allocated if at least one significant (moderate or strong, as defined above) correlation with one of the following factors was identified: clinical scales, MRI, other imaging (e.g. optical coherence tomography), genotype, clinical parameters (disease duration, age at symptom onset, age at assessment, time to manifestation in pre-clinical carriers), and (validated/non-validated) questionnaires. For studies reporting at least one significant (moderate or strong) correlation analysis for two or more domains, two points were allocated. In addition, data on the total number of studies and total number of patients that were assessed for any given oculomotor domain, and the oculomotor/vestibular changes observed within this domain, for a given HCA were gathered.

We primarily focused on the five oculomotor domains that we had previously recommended for assessment in HCAs [4], i.e. (a) saccadic eye movements (SEM), (b) pursuit eye movements (PEM), (c) fixation (including assessment of spontaneous nystagmus [SN] and saccadic intrusions (SI)), (d) eccentric gaze-holding (including presence of gaze-evoked nystagmus [GEN]) and (e) angular vestibulo-ocular reflex [aVOR], as measured with the quantitative head-impulse test [qHIT]).

We considered only those HCAs which had previously been studied by means of quantitative oculomotor/vestibular measurements and where at least two studies reporting on such results were available. For these HCAs, two raters (PG and AAT) independently calculated the scores for all five domains. Results were then merged, and discrepancies were resolved by discussion. Those domains that scored highest were further evaluated for their potential utility as oculomotor endpoints, identifying the specific paradigms that were applied in the relevant studies (e.g. visually-guided horizontal saccades for the domain “saccadic eye movements”). Taking into account the practical constraints in a clinical trial, we limited to a maximum of four oculomotor outcomes considered as most promising endpoints. Recommendations were made for their implementation and use. Furthermore, additional targeted recommendations were provided taking four specific research questions into account (1) disease characterization, (2) natural course of the disease, (3) treatment response, and (4) pre-ataxic carriers. Wherever parameter details to support recommendation were lacking (e.g. inter-stimulus interval), we adhered to the parameters previously proposed in our consensus paper [4].

Consensus for the most promising oculomotor endpoints and their recording and application recommendations was reached amongst all members of AGI working group.

## Results

### Overview of Studies

We identified 1003 citations, of which 755 (75.3%) were excluded at the abstract level and 87 (8.7%) at the full-text manuscript level (see Appendix 1 for details). From the 161 studies included after the full-text review, 130 studies (13.0%, publication year: 1974–2024) reported on 2018 patients with either genetically confirmed ataxia (n = 1776) or suspected HCA based on family history or biomarkers (n = 242; see Fig. 1 for details). Amongst genetically confirmed (or suspected) HCAs, Friedreich Ataxia (FRDA) (n = 178), spinocerebellar ataxias (SCA) (most often SCA2 [n = 463], SCA3 [n = 320] and SCA6 [n = 154]), Niemann-Pick type C (NPC) (n = 197), fragile-X tremor ataxia syndrome (FXTAS) (n = 157) and ataxia telangiectasia (A-T) (n = 96) were most frequently reported (see Table 1 for distribution of specific disorders). Few studies included pre-symptomatic carriers (n = 10 studies, 202 carriers) for selected disorders (FXTAS [14–16], NPC [17], SCA2 [18, 19], SCA3 [20–22] and SCA6 [23]).

**Table 1:** Overview of study design and clinical population across studies

	Studies (n)	Patients (n)
Gender		
Female	90	778
Male	87	815
Unclear	35	425
Total	130	2018
Study design – time line		
Prospective	119	1860
Retrospective	10	152
Unclear	1	6
Study design—location		
Monocentric	121	1924
Multicentric	9	94
Study type		
Case series	36	478
Case–control studies	78	1232
Single case reports	4	4
Observational studies	4	167
Randomized controlled treatment studies	2	71
Non-randomized treatment studies	6	66
Disease stages		
Symptomatic patients	120	1816
Pre-symptomatic carriers	10	202
Included disorders	Genetically confirmed	Based on positive family history/biochemical markers
ADCA others	49§	51
AOA1	18	NA
AOA2	27	NA
ARCA	NA	7
ARSACS	1	NA
A-T	54	42
ATLD	2	NA
AVED	2	NA
CTX	23	NA
EA	46	NA
GAA-FGF14	104	NA
Friedreich ataxia	102	76
FXTAS	157	NA
HSP	2	NA
NPC	132	65
SCA1	39	NA
SCA2	463	NA
SCA3	320	NA
SCA6	154	NA
SCA7	14	NA
SCAR4	5	NA
RFC1-related ataxia	47	NA
Various	NA	1#
Total	1776	242

§This included SCA8 (n = 7), SCA17 (n = 15), SCA31 (n = 20), SCA37 (n = 2) and other SCAs not specified (n = 5) [71–78]

#1 patient with “non-identified” genetic ataxia [79]

Abbreviations: ADCA = autosomal-dominant cerebellar ataxia; AOA = ataxia with ocular motor apraxia; ARCA = autosomal-recessive cerebellar ataxia; ARSACS = Autosomal recessive spastic ataxia of Charlevoix-Saguenay; A-T = ataxia telangiectasia; ATLD = ataxia telangiectasia like disease; AVED = ataxia with vitamin E deficiency; CTX = cerebrotendinous xanthomatosis; EA = episodic ataxia; FGF14 = fibroblast growth factor 14; FXTAS = Fragile X-Associated Tremor/Ataxia Syndrome; HSP = hereditary spastic paraparesis; NA = not available; NPC = Niemann-Pick disease type C; RFC-1 = replication factor complex subunit 1; SCA = spinocerebellar ataxia; SCAR4 = spinocerebellar ataxia, autosomal recessive 4

Study quality, with respect to the predefined criteria (see Appendix 2), was judged ‘high’ in 37 studies (28.5%), ‘moderate’ in 41 studies (31.5%) and ‘low’ in 52 studies (40.0%) (see Appendix 3, Table S1 for details). Reasons for low-quality ratings were most commonly control-group selection (e.g., non-age matched, health status omitted; n = 34 studies), risk-of-bias in result analysis (e.g., unclear or high risk; n = 23 studies) and concerns regarding statistical analyses (e.g., omission or a paucity of information regarding statistical analyses; n = 20 studies).

Sample size in the studies analyzed ranged from single case reports to larger prospective studies containing up to 103 patients [14]. The primary focus of the vast majority of studies was on the phenotypic characterization of oculomotor abnormalities in the respective disease populations (n = 113). Only five studies used eye movement recordings to monitor disease progression over periods between three months and five years; all of them were cross-sectional observational studies [20, 23–26]. Only 17 studies included the application of oculomotor testing in monitoring treatment response [27–43].

### Oculomotor Paradigms

The included studies reported on a broad range of oculomotor paradigms that were obtained with a variety of recording techniques (see Appendix 3, Tables S2-S4 for details). Specifically, most frequently, video-oculography (n = 59 studies, 1068 patients) and electro-oculography (n = 39 studies, 713 patients) were utilized, followed by scleral search coil recordings in 103 patients (n = 21 studies). Recordings were binocular in 49% of patients (984/2018) and monocular in 34% of patients (684/2018), whereas in the remaining 17% of patients (350/2018) this information was not included. Eye movement recordings were obtained for both the horizontal and vertical plane in 54% of patients (1092/2018), whereas measurements in the horizontal plane only was reported in 44% of patients (889/2018).

The oculomotor paradigms most frequently utilized included visually-guided saccadic eye movements (SEM; n = 1491 patients [94 studies]), pursuit eye movements (PEM; n = 727 patients [48 studies]), saccadic intrusions (SI; n = 478 patients [29 studies]), anti-saccades (AS; n = 398 patients [21 studies]) and gaze holding in primary gaze position (n = 539 patients [28 studies]) and at eccentric gaze (n = 609 patients [35 studies]). Quantitative head-impulse testing (n = 426 patients [19 studies]) was the most frequently applied vestibular assessment (for details see Table S4 in Appendix 3).

The specific quantitative parameters that were extracted for each oculomotor/vestibular domain varied across studies and diseases. Whereas for some HCAs quantitative oculomotor testing was obtained in all six oculomotor/vestibular domains reviewed (FRDA, SCA1/2/3/6/7, A-T), in others data on one or several oculomotor/vestibular domains were lacking (EA2, NPC, RFC1-related ataxia, SCA27B, AOA1, AOA2, FXTAS, CTX). Furthermore, the total number of patients studied for a given HCA varied substantially, ranging between 18 (AOA1) and 463 (SCA2) (see Appendix 3, Table S2 for details).

### Sensitivity to Disease Progression and Treatment Effects

Among the five longitudinal studies monitoring disease progression, three demonstrated significant changes in oculomotor parameters. In SCA2 patients, decreased visually-guided SEM peak velocity and accuracy and increased latency over an observation period of 60 months (disease duration at baseline [mean ± 1SD]: 12.3 ± 6.7 years, range = 2–34 years) was reported [25]; whereas no significant changes in SEM were observed in SCA2 patients over a shorter [12 month] period [24]. For SCA3 (disease duration at baseline = 9.3 ± 4.9 years, range 2–21 years), a significant decrease in the horizontal aVOR-gain during an interval ranging from 9 to 24 months was noted [20]. For SCA6, a significant decrease in horizontal-canal and anterior-canal aVOR-gains was observed over a follow-up period between 3 and 60 months in a single study (disease duration not reported) [26], whereas for anterior canals aVOR-gains remained unchanged. In pre-symptomatic SCA6, no alteration in oculomotor properties (SEM, PEM, SI, SN, GEN) were identified between two sequential visits (interval between visits not reported) [23].

Significant differences in oculomotor parameters were reported in 13 (out of 17) trials assessing treatment response. In several treatment trials horizontal VGS velocity after 12 months of miglustat treatment was improved [27, 28] and stabilization was shown after 24 months of treatment with miglustat [28, 29]. Likewise, improvement in horizontal VGS gain [30, 31] and self-paced saccade rate under treatment with miglustat [30] was demonstrated and saccadic peak acceleration and velocity after 12 months of treatment with miglustat became better [32]. In two randomized-controlled studies, visually-guided SEM latencies were significantly reduced in SCA2 after treatment with zinc sulfate (duration 6 months, 36 patients randomized) [33] and NeuroEPO (duration 6 months, 34 patients randomized), even in absence of proven improvement at the spinocerebellar ataxia functional index (SCAFI) [34]. For A-T patients, treatment with 4-aminopyridine (4-AP) (10 mg 4-AP single dose, 4 patients included, non-randomized) [35] and acetyl-DL-leucine (duration 1 month or more, 6 patients, non-randomized) [36] significantly decreased spontaneous nystagmus. In patients with SCA27B, 40 mg 4-AP per day significantly decreased downbeat nystagmus slow-phase velocity (duration 1 week, 4 patients randomized, cross-over design) [37]. In patients with EA4, gabapentin resulted in improved PEM and steadier gaze holding [38].

No significant changes were reported in response to idebenone in FRDA (duration 6 months to 7 years, 88 patients included, non-randomized) [39], lisuride (duration 4 weeks, 12 patients included, non-randomized) [40] and neuro-rehabilitation (duration 24 weeks, 38 patients randomized) [41] in SCA2, and to acetyl-DL-leucine in NPC (duration 4 weeks, 12 patients included, non-randomized) [42].

### Rating of Potential Oculomotor/Vestibular Endpoints in HCAs

Oculomotor/vestibular studies satisfying the criteria indicated above were identified in 15 HCAs. Overall, SEM (n = 5.5 ± 5.3 studies for every HCA, range = 0–17), was the most frequently studied paradigm with respect to other paradigms (range [average ± 1SD] = 1.8 ± 2.1 (aVOR) to 3.1 ± 3.2 (PEM) studies). SEM was also the paradigm most frequently demonstrating differences in comparison to healthy controls (4.3 ± 3.2 studies for a given HCA, while other paradigms detecting differences ranged from 0.8 ± 1.3 to 2.0 ± 2.3 studies for every HCA). Only four HCAs showed abnormalities in all six domains (FRDA, SCA3, SCA6, A-T).

Our assigned scores for given oculomotor/vestibular parameters in each HCA ranged between 0 (no significant changes observed at all) and 7 depending on the quality of the data (see Appendix 5, Tables A5-1 to A5-15). Highest scores were observed in the following HCAs: FRDA, SCA2, SCA3, SCA6, A-T, and NPC. For FRDA this was achieved when focusing on latency of visually-guided SEM, the error-rate in anti-saccades, and the frequency of SWJ [SI] (data available also on response to treatment and correlation with other clinical parameters). For SCA2 focusing on peak velocity of visually-guided SEM (data available also for pre-clinical carriers, response to treatment, disease progression and correlations with clinical parameters) resulted in a high score. In SCA3 the highest score was obtained when focusing on peak velocity of visually-guided SEM, presence of GEN, and changes in aVOR gain, as data was also available on changes in aVOR gains in pre-clinical carriers, disease progression (and resulting changes in aVOR-gain), and correlations with other clinical parameters. In SCA6 obtaining visually-guided SEM parameters (velocity, gain, accuracy), PEM gain, presence of SN, and aVOR gain (data available on correlations between aVOR gain and other [clinical] parameters) resulted in the highest score. In A-T a combination of visually-guided SEM gain, PEM gain, presence of DBN and SWJ was most promising, as also treatment responses (on DBN) and correlations with other (clinical) parameters were available. In NPC focusing on peak velocity of vertical VGS resulted in the highest score, as also treatment response to miglustat and correlations with other (clinical) parameters have been identified (see Appendix 5, Table A5-16). For most other ataxias studied, scores were 3 or lower on all domains (SCA1, SCA7, EA2, RFC1-related ataxia, AOA1, AOA2, CTX, SCA27B).

In another five HCAs we found single studies reporting on quantitative oculomotor/vestibular findings in small case series (range: 1 to 15 patients). This included the autosomal recessive spastic ataxia of Charlevoix-Saguenay (ARSACS) [43], ataxia telangiectasia-like disorder type 1 (ATLD1) [44], ataxia with vitamin E deficiency (AVED) [45], SCA17 [46], and spinocerebellar ataxia autosomal recessive 4 (SCAR4; formerly known as spinocerebellar ataxia with saccadic intrusions (SCASI) or SCA24) [47]. In these five studies, findings on one to five (out of six) oculomotor/vestibular domains were reported and details can be found in Table A7-1 in Appendix 7.

### Optimal HCA-Specific Oculomotor Paradigms

Based on the ratings, we then selected one to four oculomotor/vestibular parameters to be used as most promising oculomotor endpoints in specific HCAs (see Table 2).

**Table 2:** Preferred oculomotor/vestibular paradigms for specific hereditary ataxias in order of priority (1–4)

Disease	OM-domain 1 – selected parameter(s)	OM-domain 2 – selected parameter(s)	OM-domain 3 – selected parameter(s)	OM-domain 4 – selected parameter(s)	Significant correlations with other parameters	Longitudinal data/tx response data
FRDA	Horizontal VGS latency [80–86] AS latency [82] → Changes in VGS/AS latency	Fixation instabilities [39, 80, 81, 84, 87–92] →Presence/frequency of SWJ	None	None	VGS/AS latency with • Scales (FARS ([81]**, [82]***, [86]**), SLCLC ([81–83, 86]***)) MGS errors with • Clinical parameters (disease duration ([82]**)) • Genetics (CAA repeat length ([82]**))	Treatment response data: • No significant treatment effect of idebenone on SWJ frequency in 1 study [39]
SCA1	Horizontal VGS PV [74, 80, 93–95] →Changes in PV	Fixation instabilities [76, 80, 94] →Presence/frequency of SWJ	Eccentric gaze holding [76, 94] →Changes in gaze holding (GEN)	None	None	None/none
SCA2	Horizontal VGS PV [17, 18, 23, 24, 27, 29, 30, 46, 54–59] →Changes in PV	None	None	None	Saccadic PV with • Scales (SARA ([18]*), ICARS ([96] NR)) • Clinical parameters (age at onset ([18]**, [24] NR), time to sx onset ([97]*), disease duration ([24] NR), age ([18]**)) • MRI ([19]**) • Genetics (CAG repeat length ([18]**, [24] NR), [25]***))	Longitudinal data: • VGS PV significantly decreased over 60 months [25]. No significant changes over 12 months [24] Treatment response data: • Saccade latencies reduced after treatment with zinc sulfate [33] • Saccadic latencies decreased significantly with NeuroEPO treatment [34]
SCA3	Vertical VGS PV [21, 22, 54, 98–100] →Changes in PV	Eccentric gaze holding [22, 54, 76, 94, 99, 100] →Changes in gaze holding (GEN)	Horizontal and vertical aVOR [20, 22, 54–57] →Changes in vHIT gains	None	Saccadic PV with • Scales (SARA ([22]***), ICARS/NESSCA ([22]**), INAScount/SCAFI/CCFS ([22]*)) • Clin parameters (time to sx onset ([22]**), disease duration ([21]*)) Eccentric gaze holding deficits (GEN) with • Scales (SARA ([21, 22]***), ICARS/NESSCA/INAScount/SCAFI/CCFS ([22]***)) • Clin param (time to sx onset ([22]***), disease duration ([21]**)) HC vHIT gain with • Scales (SARA ([22, 54]***, [20]***), SARA change over time ([20]**), ICARS/NESSCA ([22]***), INAScount/SCAFI/CCFS ([22]**)) • Clinical parameters (time to sx onset ([22]**)) • Genetics (CAG repeat length ([20]*))	Longitudinal data: • Horizontal aVOR gain decreased significantly between first and second examination 9 to 24 months apart [20]
SCA6	VGS metrics [23, 26, 54, 73, 76, 94, 101] →Changes in VGS metrics	PEM [23, 26, 54, 73, 94, 95, 101–103] →Changes in pursuit gain	Gaze holding [26, 54, 73, 76, 94, 104] →Changes in vertical gaze holding (presence of DBN?)	Horizontal and vertical aVOR [26, 54, 58] →Changes in vHIT gains	HC vHIT gains with • ICARS ([58]***) • SARA ([26]***) AC and PC vHIT gains with • SARA ([26]***)	Longitudinal data: • HC and AC vHIT gains decreased significantly between first and second examination 3 to 60 months apart [26]
SCA7	Horizontal VGS PV [54, 105] →Changes in PV	PEM [54, 105] →Changes in pursuit gain	Bilateral HC and VC vHIT gains [54, 105] →Changes in vHIT gains	None	None	None/none
EA2	PEM [70, 103, 106–108] →Changes in pursuit gain	Bilateral HC and VC vHIT gains [26, 54, 58, 70, 107] →Changes in vHIT gains	Eccentric gaze holding [70, 108] →Changes in gaze holding (GEN)	None	None	None/none
A-T	VGS metrics [10, 51, 52] →Changes in VGS metrics (esp. hypometria)	PEM [10, 51–53] →Changes in pursuit gain	Gaze holding [10, 36, 67] →Changes in vertical gaze holding (presence of DBN?)	Fixation instabilities [10, 52, 67] →Presence/frequency of SWJ	Pursuit gain with • Scores (A-T index ([52]**)) • Clinical parameters (age ([52]**))	Treatment response data: • DBN SPV decreased after tx with acetyl-DL-leucine [36]
NPC	Vertical VGS PV [17, 42, 50, 63–65] →Changes in PV	None	None	None	Saccadic gain, PV, duration and self-paced saccade rate with • Scales (SARA ([65]**), mDRS ([65]**), SCAFI ([65]**), Iturriaga ([109]***)) Clinical parameters (disease duration ([109]***)) • MRI ([109–111]***, [31]**) and other imaging ([17]***)	Treatment response data: • Improvement of horizontal VGS PV and gain under miglustat [30, 31], with evaluation after 12 months [27, 28, 32]and 24 months of treatment [28, 29]
RFC1-related ataxia	Horizontal aVOR [59–62] →Changes in vHIT gains	Gaze holding [59] →Changes in vertical gaze holding (presence of DBN?)	None	None	HC vHIT gain with • Clinical parameters (disease duration ([60]*))	None/None
SCA27B	Gaze holding [37, 69] →Changes in vertical gaze holding (presence of DBN?)	Hor aVOR [37] →Changes in vHIT gains	None	None	None	Treatment response data: • DBN SPV decreased after treatment with 4-AP [37]
AOA1	VGS metrics [10, 112] and AS error rate [10, 112] →Changes in VGS metrics and AS error rate	Eccentric gaze holding [10] →Changes in gaze holding (GEN)	None	None	None	None/None
AOA2	VGS metrics [10, 113–115] and velocity [10, 113, 114]) AS error rate [10, 113–115] →Changes in VGS metrics and PV →Changes in AS error rate	Eccentric gaze holding [10] →Changes in gaze holding (GEN)	None	None	None	None/None
FXTAS	AS latency [14–16, 116] and error rate [14–16, 116] →Changes in AS latencies and error rate	None	None	None	AS latency in FXTAS patients with • Scores (BDS-2 score ([14]**), MMSE score ([14]*) AS latency in fXPCs with • Genetics (CGG repeat length ([16]*)) AS error rate in fXPCs with • Scores (BDS-2 score ([14]*), ICARS ([16]*)) VGS latency in FXTAS patients with • Scores (BDS-2 score ([14]*)) VGS metrics in fXPCs with • Scores (ICARS ([16]*)) VGS latency in fXPCs with • Genetics (CGG repeat length ([16]*)) Inhibitory cost (AS latency vs. VGS latency) in FXTAS patients • Genetics (CGG repeat length ([15]*))	None/None
CTX	Horizontal VGS metrics [45, 117] and latency [117]) AS latency [117] and error rate [117] →Changes in VGS metrics and latency →Changes in AS error rate	None	None	None	None	None/None

Abbreviations 4-AP = 4-Aminopyridine; AOA = ataxia with oculomotor apraxia; AS = anti-saccades; A-T = ataxia telangiectasia aVOR = angular vestibulo-ocular reflex; BDS = Behavioral Dyscontrol Scales; CCFS = Composite Cerebellar Functional Score; CTX = cerebrotendinous xanthomatosis; DBN = downbeat nystagmus; EA2 = episodic ataxia type 2; FARS = Friedreich *Ataxia* Rating Scale; FRDA = Friedreich Ataxia; fXPCs = fragile X premutation carriers; FXTAS = fragile-X associated tremor/ataxia syndrome; GEN = gaze-evoked nystagmus; HC = horizontal canal; Hor = horizontal; ICARS = International Cooperative *Ataxia* Rating Scale; INAScount = Inventory of Non-Ataxia Signs; mDRS = modified disability rating scale; MGS = memory-guided saccades; MMSE = mini mental state exam; MRI = magnetic resonance imaging; NESSCA = Neurological Examination Score for Spinocerebellar Ataxia; NPC = Niemann-Pick disease Type C; PEM = pursuit eye movements; PV = peak velocity; RFC1 = replication factor C subunit 1; SARA = Scale for the Assessment and Rating of Ataxia; SCA = spinocerebellar ataxia; SCAFI = Spinocerebellar Ataxia Functional Index; SLCLC = Sloan Low-Contrast Letter Chart; SPV = slow phase velocity; SWJ = square-wave jerks; tx = treatment; VGS = visually-guided saccades; vHIT = video-head-impulse test.

Single-domain oculomotor endpoints were proposed for SCA2, NPC, FXTAS and CTX. This was due to either insufficient data or non-significant findings in the other oculomotor domains tested. The most promising combination of oculomotor endpoints was selected for all other HCAs, with either two (FRDA, RFC1-related ataxia, SCA27B, AOA1, AOA2), three (SCA1, SCA3, SCA7, EA2) or four (SCA6, A-T) oculomotor domains.

While demonstrating significant changes as compared to healthy controls was a prerequisite for selection in all oculomotor parameters, data on other potentially valuable factors was less often available, and this included: treatment response (n = 5; significant changes in NPC, A-T, SCA2 and SCA27B, but not in FRDA), natural course of disease (n = 3; significant changes in SCA2, SCA3 and SCA6), significant correlations with other (clinical) parameters (n = 8; significant changes in FRDA, SCA2, SCA3, SCA6, A-T, NPC, RFC1-related ataxia and FXTAS) and presence in pre-clinical carriers (n = 4; SCA2, SCA3, SCA6, FXTAS).

Proposed parameters for selected oculomotor/vestibular paradigms were retrieved from studies and – where necessary – combined with the parameters previously published for quantitative oculomotor/vestibular testing by our group [6]. A detailed overview of the proposed preferred parameters can be found in Appendix 6, Table A6-1.

In selecting the best parameters to be used as potential endpoints, we also considered the study aim(s) as the particular research question examined in any given study may influence the selection of oculomotor/vestibular paradigms and measurements. Thus, we provided recommendations for specific oculomotor/vestibular paradigms/parameters for the different research questions in each selected HCA (Appendix 6, Table A6-2). As result, while recommendations for cross-sectional disease characterization are available for each HCA, paradigm/parameter indications for all the other three research questions listed above (natural course of disease, treatment response, pre-ataxic carriers) are possible only for SCA2. In contrast, no recommendations could be made for one or several of the other research questions in the remaining HCAs (see Appendix 6, Table A6-2 for more details).

## Discussion

In this systematic review, we evaluated quantitative oculomotor parameters as digital-motor outcomes in specific HCAs, with a focus on their utility as potential endpoints in measuring disease progression and treatment response.

We found that many of the reviewed oculomotor measures exhibit properties that support their use as endpoints in clinical trials. Indeed, beyond supporting the disease characterization and diagnosis, these parameters can sensitively detect disease progression or treatment effects in 1 to maximum 2 years.

Additionally, oculomotor endpoints can be easily standardized allowing a broader applicability and generalization of findings. Also important in relatively uncommon diseases, oculomotor endpoints may provide sufficient effect sizes even in small cohorts while maintaining low variability, a key attribute of optimal outcome measures. Finally, oculomotor parameters have direct meaningfulness for patients as visual disturbance caused, for instance, by impaired tracking, delayed or imprecise gaze shifting, or nystagmus significantly impair their quality of life [48]. However, it is important to point out that our systematic review also concludes that for most HCAs there is an incomplete quantitative account of the pathological involvement of the oculomotor function.

### Disease-Specific and Most Promising Oculomotor Endpoints

A single oculomotor endpoint sensitive enough to detect changes in the majority of affected patients could be identified only in SCA2 (peak-velocity of horizontal VGS), NPC (peak-velocity of vertical VGS), RFC1-related ataxia (both aVOR gain reduction and DBN were seen in most or even all patients) and SCA27B (DBN seen in all patients).

For this reason, we proposed combining (up to four) promising oculomotor endpoints for all other HCAs, with the exception of CTX for which quantitative data were available only for a single oculomotor domain.

Noteworthy, for RFC1-related ataxia and SCA27B a combination of aVOR gain measurements and assessment for spontaneous (downbeating) nystagmus was recommended to allow a better distinction between these two entities as aVOR gain deficits are usually more pronounced for RFC1-related ataxia compared to SCA27B [49]. However, the possibility of assessing the suitability of possible endpoint was limited by the availability of data in different domains for each HCA studied.

Overall, visually-guided saccadic eye movements (VGS) provided the largest data set, demonstrating significant changes in either horizontal or vertical peak saccadic eye velocity (SCA1, SCA2, SCA3, SCA7, NPC, AOA2), saccade metrics (SCA6, A-T, AOA1, AOA2, CTX), and/or latency (FRDA, FXTAS, CTX).

Impaired gaze fixation such as saccadic intrusions, spontaneous vertical nystagmus or impaired eccentric gaze holding (i.e. gaze-evoked nystagmus) was documented in many HCAs. Specifically, prominent square-wave jerks were identified in FRDA and SCA1, downbeat nystagmus was frequently reported in SCA6, EA2, A-T, SCA27B and RFC1-related ataxia, whereas gaze-evoked nystagmus was observed in SCA1, SCA3, SCA6, EA2, AOA1 and AOA2.

In contrast, other oculomotor/vestibular domains were less often assessed. This might result from a disease-specific preference in the selection of the oculomotor/vestibular domains, privileging the most informative or typical parameter for a given ataxia. For example, studies reporting on SCA2 patients often focused on visually-guided saccades and works on RFC1-related ataxia restricted testing to aVOR, as slow saccades and vestibular areflexia are distinctive features of these diseases, respectively.

As a consequence, most HCAs were lacking data on one or several of the oculomotor/vestibular domains that we had recommended to test [4, 6]. This was often the case of the vestibular domain with an assessment of the high-frequency angular vestibulo-ocular reflex (aVOR) tested by single studies only in some ataxias (SCA1, A-T, NPC) or completely lacking in others (AOA1, AOA2, FXTAS, CTX). The remaining ataxias (FRDA, RFC1-related ataxia, EA2, SCA2, SCA3, SCA6, and SCA27B) all had instead at least two studies in which vestibular testing results were included.

Although valuable to distinguish affected from healthy subjects and potentially useful to assess evolution, most parameters lack studies were they have been applied to test disease progression and response to treatment, preventing a conclusive judgement on their implementation as endpoints.

Indeed, data on disease progression were only available for three diseases (SCA2, SCA3, SCA6) and significant changes were observed within the required time for a biomarker to be useful in clinical trials (no more than two years) only in SCA3 [20]. In SCA2, significant changes were observed only over a period of five years [25] (with no significant change measured over 12 months probably related to the small sample size [n = 30] [24]) and in SCA6 the follow-up interval for aVOR testing (demonstrating significant decreases in horizontal and anterior canal gains) varied substantially (being between 3 and 60 months) [26].

Regarding response to treatment, significant changes in oculomotor/vestibular parameters were detected in 13 out of 17 HCA treatment trials where quantitative oculomotor outcomes were included: horizontal saccades velocity in SCA2 [33, 34] and NPC [27–32, 50], pursuit gain and nystagmus in EA4 [38], downbeat slow-phase velocity in SCA27B [37] and saccadic intrusions in A-T [35, 36].

Unfortunately, individuals with pre-symptomatic and early stages of disease, who represent the ideal target for upcoming preventive trials, were underrepresented in the published literature: studies involving pre-symptomatic subjects exist for SCA2, SCA3, SCA6, NPC, and FXTAS only.

In symptomatic patients, the reported disease duration varied widely across studies, ranging from an average of 3.9 ± 2.5 years (Costales et al., 2021) to 30.6 ± 10.2 years (Koens et al., 2022). However, oculomotor and vestibular abnormalities may evolve over the course of the disease, necessitating the selection of parameters that are optimized for specific disease stages. The current literature does not provide sufficient data to support such a stage-specific approach, highlighting the need for future studies as a priority.

Also, in constructing a tailored protocol for a given HCA, some oculomotor peculiarities of certain diseases should be considered. For example, in A-T quantitative studies record often saccadic dysmetria. However, in a subset of A-T patients (perhaps more often young-onset patients) saccades may be preceded or proceeded by a characteristic slow’drift’ movement of the eyes. When proceeding the saccade, these slow target-oriented movements result in the accurate landing of the fovea on the object [51–53]. When setting parameters for automatic saccade detection, though, algorithms, if not specifically instructed, may fail to recognize these OM patterns reporting hypometric saccades instead of atypical, but accurate, eye shifting. This emphasizes the importance of a manual examination of raw eye-movement traces (as well as bedside examination by those with adequate expertise) followed by an adjustment of the setting parameters of the algorithms when needed.

As with all instrumented motor metrics, inter-study comparisons and the generalizability of results depend on methodological consistency. Given the diversity of laboratories and the extended timeframe over which these studies were conducted resulting in variations in quantitative eye movement methodologies—including electro-oculography, scleral search coils, and video-oculography—along with recording frequencies ranging from 30 to 1200 Hz, a direct inter-study comparison is often challenging.

To fill this gap and obtain harmonized data, our group has previously provided recommendations for standardized methodologies for oculomotor assessment [4, 6].

### The Utility of Specific Oculomotor Abnormalities in Certain HCAs

Oculomotor abnormalities are common in HCAs but are rarely specific to a single diagnostic entity. However, certain oculomotor and vestibular changes occur more frequently, prominently or typically in specific HCAs. These characteristic abnormalities may be considered “core” features of a given disease and could serve as potential oculomotor endpoints.

For example, in this systematic review we identified prominent and frequent deficits in the aVOR in SCA3 [20, 22, 54–57], SCA6 [26, 54, 58], and RFC1-related ataxia [59–62]. Likewise, saccadic velocity is severely reduced in SCA2 (symptomatic patients [17, 18, 23, 24, 27, 29, 30, 46, 54–59] and pre-symptomatic individuals [18, 19]) and NPC [17, 42, 50, 63–65]. Curved vertical saccades (“round the houses sign”) are most commonly seen in NPC [66]. Dysmetric saccades [10, 51, 52] and gaze-holding deficits (SI [10, 52, 67, 68], DBN [10, 35–37, 67, 69, 70]) are prominent in A-T, EA2, SCA27B and SCASI (SCAR4).

Unlike other clinical outcome measures for ataxia, oculomotor parameters provide a unique opportunity to detect and localize dysfunction across different brain regions using the same standardized paradigms. Indeed, while some oculomotor abnormalities, such as saccadic dysmetria and impaired pursuit eye movements, specifically reflect dysfunction within intrinsic cerebellar structures and are general findings of ataxias, other features indicate specific pathology beyond the cerebellum. For instance, reduced saccadic velocity suggests brainstem involvement and is a typical feature of certain SCAs, especially SCA2, and NPC. This ability to probe multiple neural substrates with a unified set of eye movement tests makes the oculomotor assessment a powerful tool for both disease characterization and tracking neurodegeneration in HCAs.

Nevertheless, it is noteworthy to highlight that the most characteristic oculomotor abnormality of a given disease may be highly useful for distinguishing affected individuals and guiding diagnosis but is not necessarily the best marker for assessing treatment efficacy. This is exemplified by saccadic velocity in NPC. Vertical supranuclear gaze palsy, characterized by progressive vertical saccadic slowing, is a hallmark of NPC and a key diagnostic feature. However, vertical saccadic velocity proved ineffective as a clinical trial endpoint for demonstrating the efficacy of miglustat, a disease-modifying treatment for NPC. Indeed, although vertical gaze palsy is nearly ubiquitous in NPC, by the time the disease is usually diagnosed, vertical saccades are commonly severely impaired or absent, limiting their utility in tracking treatment response. In contrast, horizontal saccadic abnormalities emerge as the disease progresses but remain present to a degree that allows for quantification of treatment effects. This led to the use of the slope of the peak duration versus amplitude regression line, termed horizontal saccadic eye movement alpha (HSEM-α), as a principal outcome measure in the clinical trials of miglustat.

This example underscores the difficulty to select disease-specific oculomotor parameters as biomarkers for diagnosis and also clinical endpoints, as they might not be sensitive enough to detect treatment effects over the course of the disease [30].

### Current Limitations of Eye Movements as Potential Endpoints

The search for optimal oculomotor/vestibular endpoints in HCAs is far from over. Currently, several HCAs are lacking quantitative data for at least one of the OM/vestibular domains we have designated to be of importance. Also, only 15 HCAs met our ‘minimal study requirements’ (at least two studies reporting on quantitative OM testing). Moreover, the patterns of oculomotor/vestibular responses observed strongly depended on the disease studied.

Furthermore, most proposed endpoints here, while promising, have not yet been validated as clinical outcomes and no information could be retrieved on the minimal detectable changes (MDC) for specific paradigms and parameters. Future studies should focus on MDC and should include both pre-symptomatic and symptomatic individuals, the latter at various stages of disease severity.

Sample sizes of most studies were overall small, a common factor in the study of rare diseases, but this was not necessarily a limitation as it proves that oculomotor parameters are able to detect useful changes even in a limited number of subjects, stressing the value of these measures as outcomes for clinical trials.

Finally, our proposed oculomotor endpoints are based on a standardized evaluation by two reviewers using predefined criteria. While oculomotor and visual disturbances impact visual quality and can be objectively quantified using these parameters, the selected outcomes may not fully capture aspects that are most relevant or meaningful to patients.

## Conclusions

Our systematic review underscores the potential of quantitative oculomotor parameters as objective and reliable endpoints for clinical trials in HCAs. Beyond their well-established diagnostic utility, these measures offer a unique opportunity to track disease progression with high sensitivity. Their standardizability enhances reproducibility and facilitates broad applicability across research centers. Moreover, given the rarity of HCAs, oculomotor parameters show the added value of detecting sufficient changes even in small cohorts, making them particularly attractive for clinical trials. Importantly, even if not formally correlated with measures of patients’ relevance, these biomarkers directly relate to patients'quality of life, as they quantify oculomotor dysfunctions that significantly impact vision.

However, our review also highlights critical gaps. Despite their promise, many oculomotor parameters lack longitudinal validation and pre-symptomatic studies. Even when data are available, single parameters rarely seem capable of detecting changes within the standard time of a clinical trials (1 to 2 years). Moreover, several HCAs lack quantitative oculomotor studies and very few have comprehensive data across all oculomotor and vestibular domains, which limits the development of robust endpoints.

Additionally, the most characteristic oculomotor abnormalities of a given disease, while useful for diagnosis and progression assessment, may not always be the most sensitive endpoints for detecting treatment effects, as illustrated by vertical saccadic velocity in Niemann-Pick type C. Indeed, few of the proposed endpoints have been demonstrated to be sensitive to treatment interventions, but this is also due to the scarcity of therapeutic trials in HCAs so far. As novel approaching treatments for HCAs impose clinical trials readiness, there is an urgent need for future studies validating these oculomotor endpoints.

## Supplementary Information

Below is the link to the electronic supplementary material.

Supplementary file1 (DOCX 435 KB)

Supplementary file2 (DOCX 1149 KB)

Supplementary file3 (DOCX 253 KB)

Supplementary file4 (DOCX 447 KB)

Supplementary file5 (DOCX 35 KB)

## Data Availability

The data that support the findings of this study are available from the corresponding author upon reasonable request.
