# Supplementary material for: Best Oculomotor Endpoints for Clinical Trials in Hereditary Ataxias: A Systematic Review and Consensus by the Ataxia Global Initiative Working Group on Digital‑Motor Biomarkers
Source: Cerebellum. 2025 Aug 13;24(5):141. doi: 10.1007/s12311-025-01894-z (PMC12350468; doi:10.1007/s12311-025-01894-z)
Supplement: Supplementary file 2 — Supplementary file2 (DOCX 1149 KB) [file 12311_2025_1894_MOESM2_ESM.docx]

**Appendix 4 - Quantitative oculomotor and vestibular paradigms used in hereditary ataxias – detailed description**

# **Table A4-1: Quantitative oculomotor findings in Friedreich Ataxia (FRDA)**

| **Table A4-1: Quantitative oculomotor findings in FRDA** | | | | | | |
| --- | --- | --- | --- | --- | --- | --- |
|  |  |  |  |  |  |  |
|  | **Saccadic eye movements** | **Pursuit eye movements** | **Saccadic intrusions** | **Spontaneous nystagmus** | **Gaze-evoked nystagmus** | **Quantitative head-impulse test** |
| **Quantitative data (n studies, n patients)** | 13, 125 | 10, 107 | 11, 150 | 4, 62 | 7, 93 | 2, 29 |
| **Parameters assessed** | - Latency for VGS, MGS, AS [1-7] - Metrics for VGS, MGS, AS [1, 3-6, 8-12] - Peak velocity VGS, MGS [1-3, 5, 6, 8, 10-12] - Amplitude variability [1] | - Gain and velocity [2, 4-6, 8-10, 12, 13] | - Presence and frequency of SWJ [1, 2, 6, 8-14] - Presence of ocular flutter [2, 10, 12] | - Presence of horizontal and/or vertical SN [2, 5, 6, 12] | - Presence of eccentric gaze holding [2, 5, 6, 8, 11-13] | - Horizontal and vertical SCCs [2] - Horizontal SCCs [15] |
| **Disease stages covered (average disease duration for clinical stages)** | Clinical stages only (8.6±4.9 to 21.3±4.2 years) * | Clinical stages only (7.0±3.5 to 20.5±8.7 years) * | Clinical stages only (7.0±3.5 to 21.3±4.2 years) * | Clinical stages only (9.0±NR to 19.5±11.7 years) * | Clinical stages only (7.0±3.5 to 19.5±11.7 years) * | Clinical stages only (19.5±11.7 to 23.3±15.7 years) |
| **OM / vestibular pattern identified** | - Dysmetric saccades (VGS, MGS, AS) [1, 3-6, 8-11] - Prolonged latencies (VGS, MGS, AS) [1-7]. - Saccadic velocities normal to mildly reduced (VGS, MGS) [2, 3, 5, 6, 10-12]. - Increased proportions of errors (AS, MGS) [3] and increased error latency (AS) [3] | - PEM gain [2, 4-6, 10] and velocity [2, 8] mildly to moderately reduced | - Frequent SWJ [1, 2, 6, 8-14], ocular flutter [2, 10, 12] | - Downbeat nystagmus [2, 6] - Periodic alternating nystagmus [6] | - GEN [2, 5, 6, 8, 11-13] - RBN [6, 11] | - Reduced aVOR gain (horizontal and vertical SCCs) [2] - Reduced aVOR gain (horizontal SCCs) [15] |
| **Discrimination between patients / controls** | Assessed in 7 studies, significant differences in 6 studies [3-6, 11, 12] | Assessed in 4 studies,  significant differences in 3 studies [5, 6, 11] | Assessed in 1 study, significant differences in 1 study [1] | NA | NA | Assessed in 1 study, significant differences in 1 study [15] |
| **Longitudinal data** | NA | NA | NA | NA | NA | NA |
| **Treatment response data** | NA | NA | 1 study [14], no significant treatment effect (idebenone) | NA | NA | NA |
| **Correlation analyses (sign. corr only)§** | **FARS**   - VGS latency ([2]**, [3]***, [7]**) - AS latency ([3]***) - AS error latency ([3]**) - MGS error latency ([3]**)   **SLCLC**   - VGS latency ([2]***, [3]***, [4]***, [7]***) - MGS latency ([3]***) - AS latency ([3]***) - AS error latency ([3]**) - VGS latencies in change to direction and amplitude ([4]**)   **Disease duration**   - AS latency ([3]**) - MGS errors ([3]***)   **CAA repeats**   - MGS errors ([3]**) | NA | **Age of onset**   - Frequency of SWJ ([2]**) - SWJ duration ([2]***)   **SLCLC**   - macro SWJ ([2]***) - SWJ ([2]**) | **Disease duration**   - Downbeat nystagmus ([2]**) | NA | NA |

Abbreviations: AS=anti-saccades; aVOR=angular vestibulo-ocular reflex; FARS=Friedreich Ataxia Rating Scale; FRDA=Friedreich ataxia; GEN=gaze-evoked nystagmus; ICARS=International Cooperative Ataxia Rating Scale; MGS=memory-guided saccades; NA=not available; NPC=Niemann-Pick disease type C; OM=oculomotor; qHIT=quantitative head-impulse test; RBN=rebound nystagmus; SARA=Scale for the Assessment and Rating of Ataxia; SCA=spinocerebellar ataxia; SEM=saccadic eye movements; SI=saccadic intrusions; SLCLC= SLCLC=Sloan Low-Contrast Letter Chart; SN=spontaneous nystagmus; PEM=pursuit eye movements; SCCs=semicircular canals; SWJ=square-wave jerks; VGS=visually-guided saccades.

* Disease duration not reported in one or more studies

§ Strength of correlation (Pearson or Spearman): ***=strong correlation; **=moderate correlation; *= weak correlation

# **Table A4-2: Quantitative oculomotor findings in spinocerebellar ataxia type 1 (SCA1)**

| **Table A4-2: Quantitative oculomotor findings in SCA1** | | | | | | |
| --- | --- | --- | --- | --- | --- | --- |
|  |  |  |  |  |  |  |
|  | **Saccadic eye movements** | **Pursuit eye movements** | **Saccadic intrusions** | **Spontaneous nystagmus** | **Gaze-evoked nystagmus** | **Quantitative head-impulse test** |
| **Quantitative data (n studies, n patients)** | 7, 24 | 4, 11 | 3, 14 | 2, 8 | 2, 8 | 1, 4 |
| **Parameters assessed** | - Metrics for VGS [1, 16-18] - Latency VGS [1, 16] - Peak velocity for VGS [1, 16, 19-21] | - Pursuit velocity [22] - Pursuit gain [16, 21] | - Presence and frequency of SWJ [1, 16, 18] and ocular flutter [16, 18] | - Presence of SN [16, 18] | - Presence of GEN [16, 18] and RBN [16] | - aVOR gains [15] |
| **Disease stages covered (average disease duration for clinical stages)** | Clinical stages only (7.0±3.6 to 11.2±7.0 years) * | Clinical stages only (3 to 8 years) | Clinical stages only (7.0±3.6 to 11.2±7.0 years) | Clinical stages only (7.0±3.6 to 8.0±3.4 years) | Clinical stages only (7.0±3.6 to 8.0±3.4 years) | Clinical stages only (8.3±4.6 years) |
| **OM / vestibular pattern identified** | - Mildly to moderately reduced velocity [1, 16, 19-21] - Increased intra-subject variability [1] - Normal to increased latency [1, 16] - Mild dysmetria [16-18] | - Mildly reduced pursuit gain [16, 21] | - Frequent SWJ [1, 16, 18] | - No SN detected [16, 18] | - GEN frequently identified [16, 18] - RBN occasionally detected [16] | - Normal aVOR gains [15] |
| **Discrimination between patients / controls** | Assessed in 5 studies, significant differences in 5 studies [1, 16, 17, 19, 20] | Assessed in 4 studies,  significant differences in 3 studies [16, 21, 22] | Assessed in 2 studies, significant differences in 2 studies [1, 16] | Assessed in 1 study, no significant differences found | Assessed in 1 study, significant differences in 1 study [16] | Assessed in 1 study, no significant differences found |
| **Longitudinal data** | NA | NA | NA | NA | NA | NA |
| **Treatment response data** | NA | NA | NA | NA | NA | NA |
| **Correlation analyses (sign. Corr only)§** | NA | NA | NA | NA | NA | NA |

Abbreviations: aVOR=angular vestibulo-ocular reflex; GEN=gaze-evoked nystagmus; NA=not available; OM=oculomotor; RBN=rebound nystagmus; SEM=saccadic eye movements; SI=saccadic intrusions; SN=spontaneous nystagmus; PEM=pursuit eye movements; SWJ=square-wave jerks; VGS=visually-guided saccades.

* Disease duration not reported in one or more studies

§ Strength of correlation (Pearson or Spearman): ***=strong correlation; **=moderate correlation; *= weak correlation

# **Table A4-3: Quantitative oculomotor findings in spinocerebellar ataxia type 2 (SCA2)**

| **Table A4-3: Quantitative oculomotor findings in SCA2** | | | | | | |
| --- | --- | --- | --- | --- | --- | --- |
|  |  |  |  |  |  |  |
|  | **Saccadic eye movements** | **Pursuit eye movements** | **Saccadic intrusions** | **Spontaneous nystagmus** | **Gaze-evoked nystagmus** | **Quantitative head-impulse test** |
| **Quantitative data (n studies, n patients)** | 17, 366 | 4, 18 | 4, 25 | 3, 23 | 3, 23 | 2, 10 |
| **Parameters assessed** | - Metrics for VGS [16, 18, 23-32] - Peak velocity for VGS [16, 19, 20, 23-28, 30-35] - Latency [16, 25-28, 30-32, 35] - Latency and directional error rate in AS [35, 36] | - Pursuit gain [16, 23, 32] | - Presence and frequency of SWJ [16, 18, 23, 32] | - Presence of SN [16, 18, 32] | - Presence of GEN and RBN [16, 18, 32] | - aVOR horizontal gain [15, 32] and vertical aVOR gain [32] |
| **Disease stages covered (average disease duration for clinical stages)** | Both preclinical (carriers [26, 33]) and clinical stages (6.1±6.2 to 31.0±1.4 years) * | clinical stages only (6.1±6.2 to 31.0±1.4 years) | clinical stages only (6.1±6.2 to 31.0±1.4 years) | clinical stages only (6.1±6.2to 11.8±7.6 years) | clinical stages only (6.1±6.2 to 11.8±7.6 years) | clinical stages only (6.1±6.2 to 15.3±3.6 years) |
| **OM / vestibular pattern identified** | - Severely reduced saccadic velocity in symptomatic patients [16, 19, 20, 23, 24, 26-28, 30-35] and carriers [26, 33]. - Saccades accurate in some studies [24] and dysmetric in others [16, 18, 23, 26, 27] - Increased saccadic latency [16, 26, 27] - Prolonged latency with larger variability than normal, lower velocity and increased directional error rate in AS [36] | - Normal to mildly reduced gain [16, 23, 32] | - Increased frequency of SWJ [16, 18, 23, 32] | - No evidence for SN [16, 18] | - GEN present in part of patients [16, 18] - Absence of RBN [16] | - aVOR gain preserved [15, 32] |
| **Discrimination between patients / controls** | Assessed in 11 studies, significant differences in 11 studies [16, 18-20, 24, 26-28, 32, 34, 36] | Assessed in 2 studies, significant differences in 1 study [32] | Assessed in 3 studies, significant differences in 3 studies [16, 18, 32] | Assessed in 3 studies, no significant differences | Assessed in 2 studies, significant differences in 1 study [16] | Assessed in 2 studies, no significant differences |
| **Longitudinal data** | - Pvel and accuracy significantly decreased and latency significantly increased over 60 months [27] - No significant changes in Pvel, accuracy and latency over shorter (12 month) period [34] | NA | NA | NA | NA | NA |
| **Treatment response data** | - No changes in SEM under lisuride treatment [30] - No changes in saccade parameters (neurorehabilitation) [25] - Saccade latencies reduced after treatment with zinc sulfate [31] - Saccadic latencies decreased significantly with NeuroEPO treatment [35] | NA | NA | NA | NA | NA |
| **Correlation analyses (sign. Corr. only) §** | **SARA**   - Saccadic PV ([26]*)   **ICARS**   - Mean saccadic velocity ([24] NR) - Duration of saccades ([24] NR)   **Disease duration**   - Saccadic PV ([34] NR)   **Age**   - Saccadic PV [26]**)   **Age at sx onset**   - Saccadic PV [26]**, [34] NR)   **Time to sx onset**   - AS latency [37]**) - Saccadic PV ([38]*)   **Pontine volume**   - Saccadic PV in preclinical SCA2 ([33]**) - Saccadic PV in manifest SCA2 ([33]**)   **Anterior–posterior diameter of pontine brainstem**   - Saccadic PV in preclinical SCA2 ([33]**) - Saccadic PV in manifest SCA2 ([33]**)   **CAG repeat length**   - Saccadic PV ([26]**, [34] NR) - Progression rate of horizontal PV ([27]***) - Loss of saccadic PV ([38]*) | NR | NR | NR | NR | NR |

Abbreviations: AS=anti-saccades; aVOR=angular vestibulo-ocular reflex; GEN=gaze-evoked nystagmus; ICARS=International Cooperative Ataxia Rating Scale; NA=not available; OM=oculomotor; PV=peak velocity; qHIT=quantitative head-impulse test; RBN=rebound nystagmus; SARA=Scale for the Assessment and Rating of Ataxia; SCA=spinocerebellar ataxia; SEM=saccadic eye movements; SI=saccadic intrusions; SN=spontaneous nystagmus; PEM=pursuit eye movements; SCCs=semicircular canals; SWJ=square-wave jerks; VGS=visually-guided saccades.

* Disease duration not reported in one or more studies

§ Strength of correlation (Pearson or Spearman): ***=strong correlation; **=moderate correlation; *= weak correlation

# **Table A4-4: Quantitative oculomotor findings in spinocerebellar ataxia type 3 (SCA3)**

| **Table A4-4: Quantitative oculomotor findings in SCA3** | | | | | | |
| --- | --- | --- | --- | --- | --- | --- |
|  |  |  |  |  |  |  |
|  | **Saccadic eye movements** | **Pursuit eye movements** | **Saccadic intrusions** | **Spontaneous nystagmus** | **Gaze-evoked nystagmus** | **Quantitative head-impulse test** |
| **Quantitative data (n studies, n patients)** | 11, 197 | 6, 165 | 7, 99 | 4, 97 | 7, 166 | 6, 161 |
| **Parameters assessed** | - Metrics for VGS [1, 16, 18, 32, 39-41] - Peak velocity for VGS [1, 16, 19, 20, 32, 39-43] - Latency [1, 16, 32, 41] - Directional error rate in AS [41] | - Pursuit gain [16, 32, 40-42] | - Presence and frequency of saccadic intrusions [43] including SWJ [1, 16, 18, 32, 41] and micro-opsoclonus [40] - Amplitude of SWJ [41] | - Presence of SN [18, 32, 42] | - Presence of GEN [16, 18, 32, 40-43] and RBN [16] | - Horizontal vHIT gain [15, 32, 42, 44-46] and vertical vHIT gain [32, 46] |
| **Disease stages covered (average disease duration for clinical stages)** | Both preclinical (carriers [41, 42]) and clinical stages (4.5±2.2 to 14.8±7.0 years) * | Both preclinical (carriers [41, 42]) and clinical stages (4.5±2.2 to 10.3±3.4 years) | Both preclinical (carriers [41]) and clinical stages (4.5±2.2 to 14.8±7.0 years) * | Both preclinical (carriers [42]) and clinical stages (5.9±4.1 to 9.4±4.7 years) | Both preclinical (carriers [41, 42]) and clinical stages (4.5±2.2 to 10.3±3.4 years) * | Both preclinical (carriers [42, 45]) and clinical stages (5.9±4.1 to 9.3±4.9 years) * |
| **OM / vestibular pattern identified** | - Normal saccade latency [1, 16, 41] - Saccade velocity normal [1, 16, 19, 20] or mildly reduced [32, 39-43] - Dysmetric [18, 32, 40] or overshooting saccades [16, 39] - Increased AS error rate [41] | - Reduced pursuit gain [16, 41, 42] with frequent saccadic interruptions [40] | - Increased frequency of SWJ [1, 16, 18, 32, 41] and micro-opsoclonus [40] - Pinball intrusions [43] - Increased amplitude of SWJ [41] | - Minor or no SN [18, 42] | - Frequently present GEN [16, 18, 32, 40, 42, 43] and RBN [16] | - Reduced vHIT gain [15, 32, 42, 44-46] |
| **Discrimination between patients / controls** | Assessed in 8 studies, significant differences in 4 studies [18, 32, 39, 41] | Assessed in 3 studies, significant differences in 3 studies [32, 41, 42] | Assessed in 5 studies, significant differences in 4 studies [1, 18, 32, 41] | Assessed in 3 studies, no significant differences found | Assessed in 4 studies, significant differences in 3 studies [18, 32, 42] | Assessed in 7 studies, significant differences in 6 studies [15, 32, 42, 44-46] |
| **Longitudinal data** | NA | NA | NA | NA | NA | Horizontal VOR gain decreased significantly between first and second examination [45] |
| **Treatment response data** | NA | NA | NA | NA | NA | NA |
| **Correlation analyses (sign. Corr only)§** | **SARA**   - Reflexive vertical saccades slope ([42]***) - Upward saccadic latency ([41]*) - Horizontal saccadic PV ([41]*) - Horizontal and upward saccadic accuracy ([41]**) - Antisaccadic error rate ([41]**)   **ICARS / NESSCA**   - Vertical saccades slope ([42]**)   **INAScount / SCAFI / CCFS**   - Vertical saccades slope ([42]*)   **Time to sx onset**   - Vertical saccades slope ([42]**)   **Disease duration**   - Upward saccadic latency ([41]*) and PV ([41]*) - Horizontal ([41]*) and upward ([41]**) saccadic accuracy - Antisaccadic error rate ([41]*)   **CAG repeat length**   - Antisaccadic error rate ([41]*) | **SARA**   - Horizontal and vertical pursuit gain ([41]**)   **ICARS**   - Vertical pursuit gain ([42]*)   **SCAFI**   - Vertical pursuit gain ([42]*)   **CCFS**   - Vertical pursuit gain ([42]*)   **Disease duration**   - horizontal pursuit gain ([41]**) | **SARA**   - SWJ frequency ([41]*) - SWJ amplitude ([41]*) | **ICARS / NESSCA / SCAFI / CCFS**   - SPV SN ([42]*), | **SARA / ICARS / NESSCA / SCAFI / CCFS / INAScount**   - SPV GEN ([42]***)   **SARA**   - Frequency of horizontal GEN ([41]**) - Amplitude of horizontal GEN ([41]***)   **Time to sx onset**   - SPV GEN ([42]***)   **Disease duration**   - Frequency of horizontal GEN ([41]**) - Amplitude of horizontal GEN ([41]**) | **SARA / ICARS / NESSCA**   - HC vHIT gain ([42]***)   **SARA**   - HC vHIT gain ([45]***, [32]***)   **SCAFI / CCFS / INAScout**   - HC vHIT gain ([42]**)   **Time to sx onset**   - HC VHIT gain ([42]**)   **Change in SARA over time**   - Change in HC vHIT gain over time (longitud study) ([45]**)   **CAG repeat length**   - HC vHIT gain ([45]*) |

Abbreviations: AS=anti-saccades; aVOR=angular vestibulo-ocular reflex; CCFS=Composite Cerebellar Functional Score; GEN=gaze-evoked nystagmus; HC=horizontal canal; ICARS=International Cooperative Ataxia Rating Scale; INAScount= Inventory of Non-Ataxic Signs; NA=not available; NESSCA=Neurological Examination Score for Spinocerebellar Ataxia; OM=oculomotor; RBN=rebound nystagmus; SARA=Scale for the Assessment and Rating of Ataxia; SCA=spinocerebellar ataxia; SCAFI=SCA Functional Index; SEM=saccadic eye movements; SI=saccadic intrusions; SLCLC=Sloan Low-Contrast Letter Chart; SN=spontaneous nystagmus; SPV=slow-phase velocity; PEM=pursuit eye movements; sx=symptom; SWJ=square-wave jerks; VGS=visually-guided saccades; vHIT=video head-impulse test.

* Disease duration not reported in one or more studies

§ Strength of correlation (Pearson or Spearman): ***=strong correlation; **=moderate correlation; *= weak correlation

# **Table A4-5: Quantitative oculomotor findings in spinocerebellar ataxia type 6 (SCA6)**

| **Table A4-5: Quantitative oculomotor findings in SCA6** | | | | | | |
| --- | --- | --- | --- | --- | --- | --- |
|  |  |  |  |  |  |  |
|  | **Saccadic eye movements** | **Pursuit eye movements** | **Saccadic intrusions** | **Spontaneous nystagmus** | **Gaze-evoked nystagmus** | **Quantitative head-impulse test** |
| **Quantitative data (n studies, n patients)** | 16, 110 | 10, 72 | 6, 54 | 7, 60 | 7, 60 | 3, 33 |
| **Parameters assessed** | - Metrics for VGS [16, 18, 32, 47-51] - Latency [16, 32, 47, 49] - Peak velocity [16, 32, 47-49, 52] | - Gain [16, 21, 32, 47, 48, 50, 51, 53, 54] | - Presence and frequency of SWJ [16, 18, 32, 47, 48] | - Presence of SN [16, 18, 32, 47, 48, 50, 51] | - Presence of GEN [16, 18, 32, 47, 48, 50, 51] and RBN [16, 18, 47, 48, 51] | - HC vHIT gain [32, 50, 55] and VC vHIT gain [32, 50] |
| **Disease stages covered (average disease duration for clinical stages)** | Both preclinical (carriers [48] and clinical stages (10.3±8.0 to 19.3±6.0) * | Both preclinical (carriers [48]) and clinical stages (9.0±5.6 to 19.3±6.0) * | Both preclinical (carriers [48]) and clinical stages (10.3±8.0 to 13.2±10.0) * | Both preclinical (carriers [48]) and clinical stages (10.3±8.0 to 19.3±6.0) * | Both preclinical (carriers [48]) and clinical stages (10.3±8.0 to 19.3±6.0) * | clinical stages only (9.0±7.0 to 10.3±8.0) |
| **OM / vestibular pattern identified** | - Reduced gain [32, 47] - Normal [16] or mildly reduced peak velocity [47, 48] - Dysmetric saccades [16, 18, 32, 48, 50, 51] - Increased latency of saccades [49] | - Substantially reduced pursuit gain [16, 21, 32, 47, 48, 50, 51, 53, 54] | - SWJ in most [47, 52] or some [18] patients | - Downbeat Nystagmus in most patients [16, 18, 32, 47, 50, 52] - PAN in some [51] | - GEN [16, 18, 32, 47, 50-52] and RBN [16, 47, 51] in some patients | - HC vHIT gain reduced [50, 55] or increased [50] - AC vHIT gain increased [50] - PC vHIT gain reduced [32, 50] or increased [50] |
| **Discrimination between patients / controls** | Assessed in 7 studies, significant differences in 7 studies [16, 18, 32, 47-50] | Assessed in 8 studies, significant differences in 8 studies [16, 21, 32, 47, 48, 50, 53, 54] | Assessed in 3 studies, significant differences in 2 studies [47, 48] | Assessed in 5 studies, significant differences in 5 studies [16, 18, 32, 47, 50] | Assessed in 5 studies, significant differences in 5 studies [16, 18, 32, 47, 50] | Assessed in 3 studies, significant differences in 3 studies [32, 50, 55] |
| **Longitudinal data** | No change in SEM over time in presymptomatic carriers [48] | No change in PEM over time in presymptomatic carriers [48] | No change in SI over time in presymptomatic carriers [48] | No change in SN over time in presymptomatic carriers [48] | No change in GEN over time in presymptomatic carriers [48] | Significant decrease in HC and AC vHIT gains over time, but not for PC vHIT gains [50] |
| **Treatment response data** | NA | NA | NA | NA | NA | NA |
| **Correlation analyses (sign. corr only) §** | **ICARS**   - VGS and MGS latency ([49]**) - VGS amplitude ([49]**) | NA | NA | NA | NA | **ICARS**   - HC vHIT gain ([55]***)   **SARA**   - HC, AC and PC vHIT gain ([50]***) |

Abbreviations: AC=anterior canal; GEN=gaze-evoked nystagmus; HC=horizontal canal; ICARS=International Cooperative Ataxia Rating Scale; MGS=memory-guided saccades; NA=not available; OM=oculomotor; PAN=periodic alternating nystagmus; PC=posterior canal; qHIT=quantitative head-impulse test; RBN=rebound nystagmus; SARA=Scale for the Assessment and Rating of Ataxia; SN=spontaneous nystagmus; SWJ=square-wave jerks; VC=vertical canal; VGS=visually-guided saccades; vHIT=video head-impulse test.

* Disease duration not reported in one or more studies

§ Strength of correlation (Pearson or Spearman): ***=strong correlation; **=moderate correlation; *= weak correlation

# **Table A4-6: Quantitative oculomotor findings in spinocerebellar ataxia type 7 (SCA7)**

| **Table A4-6: Quantitative oculomotor findings in SCA7** | | | | | | |
| --- | --- | --- | --- | --- | --- | --- |
|  |  |  |  |  |  |  |
|  | **Saccadic eye movements** | **Pursuit eye movements** | **Saccadic intrusions** | **Spontaneous nystagmus** | **Gaze-evoked nystagmus** | **Quantitative head-impulse test** |
| **Quantitative data (n studies, n patients)** | 2, 11 | 2, 11 | 1, 9 | 1, 9 | 1, 9 | 1, 9 |
| **Parameters assessed** | - Metrics for VGS [32] - Latency of VGS [56] - Peak velocity [32, 56] | - Pursuit gain [32, 56] | Presence of SWJ [32] | Presence of SN [32] | Presence of GEN [32] | HC and VC vHIT gain [32] |
| **Disease stages covered (average disease duration for clinical stages)** | clinical stages only (4 years to 5.4±2.9) | clinical stages only (4 years to 5.4±2.9) | clinical stages only (5.4±2.9) | clinical stages only (5.4±2.9) | clinical stages only (5.4±2.9) | clinical stages only (5.4±2.9) |
| **OM / vestibular pattern identified** | - Reduced VGS peak velocity [32, 56] - Dysmetric VGS [32] - Increased VGS latency [56] | - Pursuit gain significantly reduced [32, 56] | No SI | No SN | No GEN | Reduced VC vHIT gain [32] |
| **Discrimination between patients / controls** | Assessed in 1 study, significant differences in 1 study [32] | Assessed in 1 study, significant differences in 1 study [32] | Assessed in 1 study, no significant differences found | Assessed in 1 study, no significant differences found | Assessed in 1 study, no significant differences found | Assessed in 1 study, significant differences in 1 study [32] |
| **Longitudinal data** | NA | NA | NA | NA | NA | NA |
| **Treatment response data** | NA | NA | NA | NA | NA | NA |
| **Correlation analyses (sign. corr only) §** | NA | NA | NA | NA | NA | NA |

Abbreviations: GEN=gaze-evoked nystagmus; HC=horizontal canal; NA=not available; OM=oculomotor; qHIT=quantitative head-impulse test; RBN=rebound nystagmus; SN=spontaneous nystagmus; SWJ=square-wave jerks; VC=vertical canal; VGS=visually-guided saccades; vHIT=video head-impulse test.

* Disease duration not reported in one or more studies

§ Strength of correlation (Pearson or Spearman): ***=strong correlation; **=moderate correlation; *= weak correlation

# **Table A4-7: Quantitative oculomotor findings in episodic ataxia type 2 (EA2)**

| **Table A4-7: Quantitative oculomotor findings in EA2** | | | | | | |
| --- | --- | --- | --- | --- | --- | --- |
|  |  |  |  |  |  |  |
|  | **Saccadic eye movements** | **Pursuit eye movements** | **Saccadic intrusions** | **Spontaneous nystagmus** | **Gaze-evoked nystagmus** | **Quantitative head-impulse test** |
| **Quantitative data (n studies, n patients)** | 3, 31 | 5, 36 | 0, 0 | 3, 23 | 2, 29 | 2, 21 |
| **Parameters assessed** | - Peak velocity [23, 57, 58] - Latency of VGS [57] - Metrics for VGS [23, 57, 58] | - Pursuit gain [23, 54, 57-59] | NA | - Presence of SN [23, 57, 60] | - Presence of GEN and RBN [57, 58] | - HC and VC vHIT gain [57, 59] |
| **Disease stages covered (average disease duration for clinical stages)** | clinical stages only (17.4±15.5 to 36 years)* | clinical stages only (17.4±15.5 to 36 years)* | NA | clinical stages only (8.5±7.9 to 36 years) | clinical stages only (17.4±15.5 years)* | clinical stages only (17.4±15.5 years)* |
| **OM / vestibular pattern identified** | - VGS peak velocity reduced [23] or within normal range [57, 58] - VGS latency normal [57] - VGS gain normal to slightly reduced [23] or slightly increased [57, 58] | - Pursuit gain significantly reduced [23, 54, 57-59] | NA | - Downbeat nystagmus [23, 57, 60] | - GEN and RBN in most patients [57, 58] | - HC and/or VC vHIT gain mildly reduced [57, 59] or normal [59] |
| **Discrimination between patients / controls** | NA | Assessed in 2 study, significant differences in 2 studies [54, 59] | NA | NA | NA | Assessed in 1 study, significant differences in 1 study [59] |
| **Longitudinal data** | NA | NA | NA | NA | NA | NA |
| **Treatment response data** | NA | NA | NA | NA | NA | NA |
| **Correlation analyses (sign. corr only) §** | NA | NA | NA | NA | NA | NA |

Abbreviations: GEN=gaze-evoked nystagmus; HC=horizontal canal; NA=not available; OM=oculomotor; qHIT=quantitative head-impulse test; RBN=rebound nystagmus; SN=spontaneous nystagmus; SWJ=square-wave jerks; VC=vertical canal; VGS=visually-guided saccades; vHIT=video head-impulse test.

* Disease duration not reported in one or more studies

§ Strength of correlation (Pearson or Spearman): ***=strong correlation; **=moderate correlation; *= weak correlation

# **Table A4-8: Quantitative oculomotor findings in Ataxia Telangiectasia (A-T)**

| **Table A4-8: Quantitative oculomotor findings in Ataxia Telangiectasia** | | | | | | |
| --- | --- | --- | --- | --- | --- | --- |
|  |  |  |  |  |  |  |
|  | **Saccadic eye movements** | **Pursuit eye movements** | **Saccadic intrusions** | **Spontaneous nystagmus** | **Gaze-evoked nystagmus** | **Quantitative head-impulse test** |
| **Quantitative data (n studies, n patients)** | 6, 70 | 6, 78 | 3, 63 | 5, 46 | 4, 41 | 1, 4 |
| **Parameters assessed** | - Latency [61-63] - Metrics [62-65] - Peak velocity [63, 64] - Directional error rate in AS [63] | - Pursuit gain [61-64, 66] | - Presence, amplitude and frequency of SWJ [62, 63, 67] and ocular flutter [62, 67] | - Presence of SN [61, 63, 66-68] | - Presence of GEN [63, 65-67] | - vHIT gain [66] |
| **Disease stages covered (average disease duration for clinical stages)** | clinical stages only (9.4±2.1 to 25 years mean) * | clinical stages only (13.3±5.4 to 25±NR years) * | clinical stages only (25 years mean) * | clinical stages only (13.3±5.4 to 25±NR years) * | clinical stages only (13.3±5.4 to 25±NR years) * | clinical stages only (13.3±5.4 years) |
| **OM / vestibular pattern(s) identified** | - Normal [63] or increased latency [61, 62], - Hypometric saccades [62-64] - Hypermetric saccades [65] - Normal peak velocity [63, 64] - Increased AS error rate [63] | - Reduced pursuit gain [61-64] | - SWJ and/or ocular flutter in most patients [62, 63, 67] | - Horizontal-torsional SN [61, 67] - Vertical (downbeating) SN [63, 66-68] - Periodic alternating nystagmus [67, 68] | - Horizontal GEN [63] | - Normal to increased vHIT gains [66] |
| **Discrimination between patients / controls** | Assessed in 5 studies, significant differences in 5 studies [61-65] | Assessed in 2 studies, significant differences in 2 studies [62, 64] | Assessed in 2 studies, significant differences in 1 study [62] | Assessed in 1 study, significant differences in 1 study [63] | Assessed in 1 study, significant differences in 1 study [63] | NA |
| **Longitudinal data** | NA | NA | NA | NA | NA | NA |
| **Treatment response data** | NA | NA | NA | - PAN and SN SPV decreased after treatment with 4-AP [68] - DBN SPV decreased after treatment with acetyl-DL-leucine [66] | NA | NA |
| **Correlation analyses (sign. corr. only) §** | NA | **A-T index**   - Pursuit gain ([62]**)   **Age**   - Pursuit gain ([62]**) | NA | NA | NA | NA |

Abbreviations: 4-AP=4-Aminopyridine; AS=anti-saccades; A-T=ataxia telangiectasia; DBN=downbeat nystagmus; GEN=gaze-evoked nystagmus; NA=not available; OM=oculomotor; PAN=periodic alternating nystagmus; qHIT=quantitative head-impulse test; RBN=rebound nystagmus; SN=spontaneous nystagmus; SPV=slow-phase velocity; SWJ=square-wave jerks; vHIT=video head-impulse test.

* Disease duration not reported in one or more studies

§ Strength of correlation (Pearson or Spearman): ***=strong correlation; **=moderate correlation; *= weak correlation

# **Table A4-9: Quantitative oculomotor findings in Niemann-Pick disease type C (NPC)**

| **Table A4-9: Quantitative oculomotor findings in NPC** | | | | | | |
| --- | --- | --- | --- | --- | --- | --- |
|  |  |  |  |  |  |  |
|  | **Saccadic eye movements** | **Pursuit eye movements** | **Saccadic intrusions** | **Spontaneous nystagmus** | **Gaze-evoked nystagmus** | **Quantitative head-impulse test** |
| **Quantitative data (n studies, n patients)** | 17, 202 | 3, 44 | 0, 0 | 0, 0 | 2, 84 | 1, 8 |
| **Parameters assessed** | - Peak velocity [69-84] - Latency [72, 79-81, 83, 84] - Metrics [72-74, 78, 80, 81, 83-85] - Anti-saccade error rate [78, 79, 83, 84] | - Pursuit gain [69-71, 81] | NA | NA | - Presence of GEN [69, 81] | - HC vHIT gain [86] |
| **Disease stages covered (average disease duration for clinical stages)** | clinical stages only (4.4±3.5 to 18.0±12.4 years) * | clinical stages only (13.2±9.0 to 14.0±6.5 years) * | NA | NA | clinical stages only (13.2±9.0 to 13.7±4.1 years) | clinical stages only (13.7±4.1 years) |
| **OM / vestibular pattern identified** | - Marked slowing of vertical VGS [69-71, 74, 80, 81] - Reduced horizontal VGS velocity [79-81] - Hypometric vertical [74, 80, 81] and horizontal [79, 83] VGS - Reduced number of self-paced horizontal saccades [81, 83] - Increased error rate in anti-saccades [78, 79, 83] | - Pursuit gains preserved [71] - Horizontal pursuit gain preserved [81] - Vertical pursuit gain reduced [81] | NA | NA | NA | - Normal HC vHIT gain [86] |
| **Discrimination between patients / controls** | Assessed in 10 study, significant differences in 7 study [74, 76, 78-81, 83] | Assessed in 3 studies, significant differences in 2 studies [80, 81] | NA | NA | Assessed in 2 study, no significant differences | Assessed in 1 study, no significant differences |
| **Longitudinal data** | NA | NA | NA | NA | NA | NA |
| **Treatment response data** | - no changes of SEM after 4 weeks of acetyl-DL-leucine [69] - Improvement of horizontal VGS velocity after 12 months of miglustat treatment [75, 76] and stabilization after 24 months of treatment with miglustat [75, 77] - Improvement in horizontal VGS gain [78, 85] and self-paced saccade rate under treatment with miglustat [78] - Improvement of saccadic peak acceleration and velocity after 12 months of treatment with miglustat [82] | - no changes of horizontal and vertical PEM gain after 4 weeks of acetyl-DL-leucine [69] | NA | NA | no changes of GEN after 4 weeks of acetyl-DL-leucine [69] | NA |
| **Correlation analyses (sign. Corr only) §** | **Total cerebellar volume**   - reflexive saccadic gain ([72]***)   **Total cerebellar grey matter**   - volitional saccadic gain ([72]***)   **Midbrain midsagittal area**   - saccadic PV and gain ([72]**) - Horizonal saccadic PV ([83]***)   **Pontine midsagittal area**   - Horizonal saccadic latency ([83]***)   **Pontine-midbrain-ratio**   - saccadic PV and gain ([72]***) - Horizonal saccadic PV ([83]***)   **Left / right rostal mid-frontal area**   - Horizonal self-paced saccade rate ([83]***)   **Change in cerebellar grey and white matter volume**   - Change in horizontal saccade amplitude ([85]**)   **Total callosal area**   - Horizonal saccadic PV ([84]***) - Horizonal saccadic amplitude ([84]***) - Self-paced horizontal saccade rate ([84]***)   **Callosal thickness**   - Self-paced horizontal saccade rate ([84]***)   **Averaged pRNFL**   - Upward vertical saccade amp ([70]***) - Horizontal saccadic PV ([70]***) - Duration of horizontal saccades ([70]***)   **Averaged GCIP**   - Upward vertical saccade amp ([70]***) - Horizontal saccadic PV ([70]***) - Vertical saccadic gain ([70]***)   **Disease severity (mDRS)**   - Horizonal saccadic PV ([81]**) - Upward and downward saccade amplitude ([81]**) - Downward saccade duration ([81]**)   **Disease severity (SARA)**   - Downward saccade duration ([81]**)   **Disease severity (SCAFI)**   - Horizontal saccadic latency ([81]**)   **Disease severity (Iturriaga)**   - Horizonal saccadic PV ([83]***) - Horizontal saccadic gain ([83]***)   **Disease duration**   - Horizonal saccadic PV ([83]***) - Horizontal saccadic gain ([83]***) - Anti-saccade error rate ([83]***) | **Disease severity (SARA)**   - Vertical PEM gain ([81]*)   **Disease severity (mDRS)**   - Vertical PEM gain ([81]*) | NA | NA | NA | NA |

Abbreviations: GCIP= ganglion cell and inner plexiform layer; GEN=gaze-evoked nystagmus; mDRS=modified disability rating scale; NA=not available; NPC=Niemann-Pick disease type C; OM=oculomotor; PV=peak velocity; RBN=rebound nystagmus; SARA=Scale for the Assessment and Rating of Ataxia; SCA; SCAFI=Spinocerebellar

Ataxia Functional Index; SEM=saccadic eye movements; SI=saccadic intrusions; SN=spontaneous nystagmus; PEM=pursuit eye movements; pRNFL=peripapillary retinal nerve fiber layer thickness; SWJ=square-wave jerks; vHIT=video head-impulse test.

* Disease duration not reported in one or more studies

§ Strength of correlation (Pearson or Spearman): ***=strong correlation; **=moderate correlation; *= weak correlation

# **Table A4-10: Quantitative oculomotor findings in RFC-1 related ataxia**

| **Table A4-10: Quantitative oculomotor findings in RFC-1 related ataxia** | | | | | | |
| --- | --- | --- | --- | --- | --- | --- |
|  |  |  |  |  |  |  |
|  | **Saccadic eye movements** | **Pursuit eye movements** | **Saccadic intrusions** | **Spontaneous nystagmus** | **Gaze-evoked nystagmus** | **Quantitative head-impulse test** |
| **Quantitative data (n studies, n patients)** | 0, 0 | 0, 0 | 0, 0 | 1, 11 | 0, 0 | 4, 47 |
| **Parameters assessed** | NA | NA | NA | - Presence of SN [87] | NA | - vHIT gain [87-90] - Response to caloric irrigation [90] |
| **Disease stages covered (average disease duration for clinical stages)** | NA | NA | NA | clinical stages only (3.9±2.5 years) | NA | clinical stages only (3.9±2.5 to 12 years) |
| **OM / vestibular pattern identified** | NA | NA | NA | - Downbeat nystagmus [87] | NA | - Bilaterally reduced vHIT [87-90] - Bilaterally reduced response to caloric irrigation [90] |
| **Discrimination between patients / controls** | NA | NA | NA | NA | NA | Assessed in 4 studies, significant differences in 4 studies [87-90] |
| **Longitudinal data** | NA | NA | NA | NA | NA | NA |
| **Treatment response data** | NA | NA | NA | NA | NA | NA |
| **Correlation analyses (sign. corr. only) §** | NA | NA | NA | NA | NA | **Disease duration**   - vHIT gain ([88]*) |

Abbreviations: NA=not available; OM=oculomotor; RFC-1=replication factor C subunit 1; SN=spontaneous nystagmus; vHIT=video head-impulse test.

* Disease duration not reported in one or more studies

§ Strength of correlation (Pearson or Spearman): ***=strong correlation; **=moderate correlation; *= weak correlation

# **Table A4-11: Quantitative oculomotor findings in ataxia with oculomotor apraxia type 1 (AOA1)**

| **Table A4-11: Quantitative oculomotor findings in AOA1** | | | | | | |
| --- | --- | --- | --- | --- | --- | --- |
|  |  |  |  |  |  |  |
|  | **Saccadic eye movements** | **Pursuit eye movements** | **Saccadic intrusions** | **Spontaneous nystagmus** | **Gaze-evoked nystagmus** | **Quantitative head-impulse test** |
| **Quantitative data (n studies, n patients)** | 2, 18 | 1, 12 | 1, 12 | 1, 12 | 1, 12 | 0, 0 |
| **Parameters assessed** | - Latencies for VGS [63, 91] - Peak velocities for VGS [63] - Metrics for VGS [63, 91] - Directional error rate in AS [63, 91] | - Pursuit gain [63] | - Presence, amplitude and frequency of SWJ [63] | - Presence of SN [63] | - Presence of GEN [63] | NA |
| **Disease stages covered (average disease duration for clinical stages)** | clinical stages only (avg. 23.5 [5-42 yrs] to 29.8±14.8 years) | clinical stages only (avg. 23.5 [5-42 yrs]) | clinical stages only (avg. 23.5 [5-42 yrs]) | clinical stages only (avg. 23.5 [5-42 yrs]) | clinical stages only (avg. 23.5 [5-42 yrs]) | NA |
| **OM / vestibular pattern identified** | - Dysmetric horizontal VGS (hypometric centrifugal and hypermetric or hypometric centripetal) [63, 91] - Decreased horizontal and vertical VGS velocity [63] - Increased VGS latency in some [63] - Increased AS error rate [63, 91] | NA | - SWJ in some patients [63] | - DBN in some patients [63] | - Horizontal GEN [63] | NA |
| **Discrimination between patients / controls** | Assessed in 2 study, significant differences in 2 [63, 91] | Assessed in 1 study, no significant differences | Assessed in 1 study, no significant differences | Assessed in 1 study, no significant differences | Assessed in 1 study, significant differences in 1 [63] | NA |
| **Longitudinal data** | NA | NA | NA | NA | NA | NA |
| **Treatment response data** | NA | NA | NA | NA | NA | NA |
| **Correlation analyses (sign. corr only)§** | NA | NA | NA | NA | NA | NA |

Abbreviations: AS=anti-saccades; DBN=downbeat nystagmus; NA=not available; GEN=gaze-evoked nystagmus; OM=oculomotor; SWJ=square-wave jerks; VGS=visually-guided saccades

§ Strength of correlation (Pearson or Spearman): ***=strong correlation; **=moderate correlation; *= weak correlation

# **Table A4-12: Quantitative oculomotor findings in ataxia with oculomotor apraxia type 2 (AOA2)**

| **Table A4-12: Quantitative oculomotor findings in AOA2** | | | | | | |
| --- | --- | --- | --- | --- | --- | --- |
|  |  |  |  |  |  |  |
|  | **Saccadic eye movements** | **Pursuit eye movements** | **Saccadic intrusions** | **Spontaneous nystagmus** | **Gaze-evoked nystagmus** | **Quantitative head-impulse test** |
| **Quantitative data (n studies, n patients)** | 5, 27 | 1, 11 | 2, 13 | 1, 11 | 1, 11 | 0, 0 |
| **Parameters assessed** | - Latencies for VGS [63, 92-95] - Peak velocities for VGS [63, 93, 94] - Metrics for VGS [63, 93-95] - Latency, velocity and metrics for MGS [95] - Latency [93] and directional error rate in AS [63, 93-95] | - Pursuit gain [63] | - Presence, amplitude and frequency of SWJ [63, 92] | - Presence of SN [63] | - Presence of GEN [63] | NA |
| **Disease stages covered (average disease duration for clinical stages)** | clinical stages only (15.6±9.9 to 26±14 years) | clinical stages only (avg. 25.3 [9.7-41 yrs]) | clinical stages only (21.5±5.0 to 25.3 [9.7-41] years) | clinical stages only (avg. 25.3 [9.7-41 yrs]) | clinical stages only (avg. 25.3 [9.7-41 yrs]) | NA |
| **OM / vestibular pattern identified** | - Hypometric centrifugal horizontal saccades and hypermetric centripetal horizontal saccades [63] - Hypometric horizontal [93-95] and vertical [93] VGS - Decreased horizontal [63, 93, 95] and vertical [63] VGS velocity - Increased VGS latency [63, 92, 94] - Increased latency for MGS [95] - Hypometric MGS [95] - Increased AS latency [93] - Increased AS error rate [63, 93-95] | NA | - SWJ in some patients [63, 92] | - DBN in some patients [63] | - Horizontal GEN [63] | NA |
| **Discrimination between patients / controls** | Assessed in 5 studies, significant differences in 5 [63, 92-95] | Assessed in 1 study, no significant differences | Assessed in 2 studies, significant differences in 2 [63, 92] | Assessed in 1 study, significant differences in 1 [63] | Assessed in 1 study, significant differences in 1 [63] | NA |
| **Longitudinal data** | NA | NA | NA | NA | NA | NA |
| **Treatment response data** | NA | NA | NA | NA | NA | NA |
| **Correlation analyses (sign. corr only)§** | NA | NA | NA | NA | NA | NA |

Abbreviations: AS=anti-saccades; DBN=downbeat nystagmus; NA=not available; GEN=gaze-evoked nystagmus; MGS=memory-guided saccades; OM=oculomotor; SWJ=square-wave jerks; VGS=visually-guided saccades

§ Strength of correlation (Pearson or Spearman): ***=strong correlation; **=moderate correlation; *= weak correlation

# **Table A4-13: Quantitative oculomotor findings in fragile-X tremor ataxia syndrome (FXTAS)**

| **Table A4-13: Quantitative oculomotor findings in FXTAS** | | | | | | |
| --- | --- | --- | --- | --- | --- | --- |
|  |  |  |  |  |  |  |
|  | **Saccadic eye movements** | **Pursuit eye movements** | **Saccadic intrusions** | **Spontaneous nystagmus** | **Gaze-evoked nystagmus** | **Quantitative head-impulse test** |
| **Quantitative data (n studies, n patients)** | 5, 178 | 1, 21 | 1, 22 | 0, 0 | 0, 0 | 0, 0 |
| **Parameters assessed** | - Latencies for VGS [96-99] - Peak velocities for VGS [96-98] - Metrics for VGS [97-99] - Latency, metrics and velocity of MGS [98] - Latency [96-99], gain [98, 99], velocity [98] and directional error rate in AS [96-98] - Frequency and duration of fixations of an image [100] | - Pursuit gain [97] | - Presence of SWJ [99] | NA | NA | NA |
| **Disease stages covered (average disease duration for clinical stages)** | Both preclinical carriers [96, 97, 99] and clinical stages° | Preclinical carriers only [97] | Preclinical carriers only [99] | NA | NA | NA |
| **OM / vestibular pattern identified** | - Increased latency in VGS overlap paradigm [98] - Increased latency in MGS [98] - Hypometric 30° MGS [98] - Increased AS latencies in carriers [97, 99] and in patients [96] - Increased AS error rate [96] - Fewer and shorter fixations [100] | NA | NA | NA | NA | NA |
| **Discrimination between patients / controls** | Assessed in 5 studies, significant differences in 5 [96-100] | Assessed in 1 study, no significant differences | Assessed in 1 study, no significant differences | NA | NA | NA |
| **Longitudinal data** | NA | NA | NA | NA | NA | NA |
| **Treatment response data** | NA | NA | NA | NA | NA | NA |
| **Correlation analyses (sign. corr only) §** | **BDS-2 score**   - AS latency in patients [96]** - VGS latency in patients [96]* - AS error rate in carriers [96]*   **MMSE score**   - AS latency in patients [96]*   **CGG repeat length**   - Inhibitory cost [97]* - VGS latency [99]* and AS latency [99]* in carriers   **ICARS**   - VGS metrics in carriers [99]* - AS error rate in carriers [99]* | NA | NA | NA | NA | NA |

Abbreviations: AS=anti-saccades; NA=not available; MGS=memory-guided saccades; OM=oculomotor; VGS=visually-guided saccades

° Disease duration not reported in one or more studies

§ Strength of correlation (Pearson or Spearman): ***=strong correlation; **=moderate correlation; *= weak correlation

# **Table A4-14: Quantitative oculomotor findings in cerebrotendinous xanthomatosis (CTX)**

| **Table A4-14: Quantitative oculomotor findings in CTX** | | | | | | |
| --- | --- | --- | --- | --- | --- | --- |
|  |  |  |  |  |  |  |
|  | **Saccadic eye movements** | **Pursuit eye movements** | **Saccadic intrusions** | **Spontaneous nystagmus** | **Gaze-evoked nystagmus** | **Quantitative head-impulse test** |
| **Quantitative data (n studies, n patients)** | 2, 23 | 0, 0 | 1, 4 | 0, 0 | 0, 0 | 0, 0 |
| **Parameters assessed** | - Latencies for VGS [101] - Peak velocities for VGS [101] - Metrics for VGS [101, 102] - Latency [101] and directional error rate [101] in AS | NA | - Presence of saccadic intrusions [102] | NA | NA | NA |
| **Disease stages covered (average disease duration for clinical stages)** | Clinical stages only (9.2±8.6 to 30.6±10.2 years) | NA | Clinical stages only (30.6±10.2 years) | NA | NA | NA |
| **OM / vestibular pattern identified** | - Dysmetric VGS [102] - Hypometric horizontal VGS in those CTX patients without DN involvement [101] - Increased percentage of multistep horizontal and vertical VGS [101] - Increased VGS latency in those CTX patients with DN involvement [101] - Increased AS error rate in CTX patients with or without DN involvement [101] - Increased AS latency in CTX patients with DN involvement [101] | NA | - Microsaccadic oscillations in some patients [102] | NA | NA | NA |
| **Discrimination between patients / controls** | Assessed in 1 study, significant differences in 1 [101] | NA | NA | NA | NA | NA |
| **Longitudinal data** | NA | NA | NA | NA | NA | NA |
| **Treatment response data** | NA | NA | NA | NA | NA | NA |
| **Correlation analyses (sign. corr only)§** | NA | NA | NA | NA | NA | NA |

Abbreviations: AS=anti-saccades; DN=dentate nucleus; NA=not available; OM=oculomotor; VGS=visually-guided saccades

§ Strength of correlation (Pearson or Spearman): ***=strong correlation; **=moderate correlation; *= weak correlation

# **Table A4-15: Quantitative oculomotor findings in SCA27B**

| **Table A4-15: Quantitative oculomotor findings in SCA27B** | | | | | | |
| --- | --- | --- | --- | --- | --- | --- |
|  |  |  |  |  |  |  |
|  | **Saccadic eye movements** | **Pursuit eye movements** | **Saccadic intrusions** | **Spontaneous nystagmus** | **Gaze-evoked nystagmus** | **Quantitative head-impulse test or caloric irrigation** |
| **Quantitative data (n studies, n patients)** | 0, 0 | 0, 0 | 0, 0 | 2, 6 | 0, 0 | 2, 104 |
| **Parameters assessed** | NA | NA | NA | - Presence of SN [103, 104] | NA | - aVOR gains [103, 104] |
| **Disease stages covered (average disease duration for clinical stages)** | NA | NA | NA | Clinical stages only (2 to 12 years, range) | NA | Clinical stages only (2 to 12 years, range) |
| **OM / vestibular pattern identified** | NA | NA | NA | - DBN in all patients [103, 104] | NA | - Bilaterally reduced aVOR gains in some patients [103] |
| **Discrimination between patients / controls** | NA | NA | NA | Assessed in 1 study, significant differences in 1 [103] | NA | Assessed in 1 study, significant differences in 1 [103] |
| **Longitudinal data** | NA | NA | NA | NA | NA | NA |
| **Treatment response data** | NA | NA | NA | - DBN SPV decreased after treatment with 4-AP [103] | NA | NA |
| **Correlation analyses (sign. corr. only) §** | NA | NA | NA | NA | NA | NA |

Abbreviations: 4-AP=4-aminopyridine; DBN=downbeat nystagmus; NA=not available; SPV=slow-phase velocity.

§ Strength of correlation (Pearson or Spearman): ***=strong correlation; **=moderate correlation; *= weak correlation

# **References**

[1] Alexandre MF, Rivaud-Péchoux S, Challe G, Durr A and Gaymard B. Functional consequences of oculomotor disorders in hereditary cerebellar ataxias. Cerebellum (London, England) 2013: 12:396-405. doi 10.1007/s12311-012-0433-z

[2] Fahey MC, Cremer PD, Aw ST, Millist L, Todd MJ, White OB, Halmagyi M, Corben LA, Collins V, Churchyard AJ, Tan K, Kowal L and Delatycki MB. Vestibular, saccadic and fixation abnormalities in genetically confirmed Friedreich ataxia. Brain : a journal of neurology 2008: 131:1035-45. doi 10.1093/brain/awm323

[3] Fielding J, Corben L, Cremer P, Millist L, White O and Delatycki M. Disruption to higher order processes in Friedreich ataxia. Neuropsychologia 2010: 48:235-42. doi 10.1016/j.neuropsychologia.2009.09.009

[4] Hocking DR, Corben LA, Fielding J, Cremer PD, Millist L, White OB and Delatycki MB. Saccade reprogramming in Friedreich ataxia reveals impairments in the cognitive control of saccadic eye movement. Brain and cognition 2014: 87:161-7. doi 10.1016/j.bandc.2014.03.018

[5] Moschner C, Perlman S and Baloh RW. Comparison of oculomotor findings in the progressive ataxia syndromes. Brain : a journal of neurology 1994: 117 ( Pt 1):15-25. doi 10.1093/brain/117.1.15

[6] Wessel K, Moschner C, Wandinger KP, Kömpf D and Heide W. Oculomotor testing in the differential diagnosis of degenerative ataxic disorders. Archives of neurology 1998: 55:949-56. doi 10.1001/archneur.55.7.949

[7] Hocking DR, Fielding J, Corben LA, Cremer PD, Millist L, White OB and Delatycki MB. Ocular motor fixation deficits in Friedreich ataxia. Cerebellum (London, England) 2010: 9:411-8. doi 10.1007/s12311-010-0178-5

[8] Baloh RW, Konrad HR and Honrubia V. Vestibulo-ocular function in patients with cerebellar atrophy. Neurology 1975: 25:160-8. doi 10.1212/wnl.25.2.160

[9] Ciuffreda KJ, Kenyon RV and Stark L. Eye movements during reading: further case reports. American journal of optometry and physiological optics 1985: 62:844-52. doi 10.1097/00006324-198512000-00005

[10] Ell J, Prasher D and Rudge P. Neuro-otological abnormalities in Friedreich's ataxia. Journal of neurology, neurosurgery, and psychiatry 1984: 47:26-32. doi 10.1136/jnnp.47.1.26

[11] Furman JM, Perlman S and Baloh RW. Eye movements in Friedreich's ataxia. Archives of neurology 1983: 40:343-6. doi 10.1001/archneur.1983.04050060043006

[12] Spieker S, Schulz JB, Petersen D, Fetter M, Klockgether T and Dichgans J. Fixation instability and oculomotor abnormalities in Friedreich's ataxia. Journal of neurology 1995: 242:517-21. doi 10.1007/bf00867423

[13] Dale RT, Kirby AW and Jampel RS. Square wave jerks in Friedreich's ataxia. American journal of ophthalmology 1978: 85:400-6. doi 10.1016/s0002-9394(14)77738-4

[14] Ribaï P, Pousset F, Tanguy ML, Rivaud-Pechoux S, Le Ber I, Gasparini F, Charles P, Béraud AS, Schmitt M, Koenig M, Mallet A, Brice A and Dürr A. Neurological, cardiological, and oculomotor progression in 104 patients with Friedreich ataxia during long-term follow-up. Archives of neurology 2007: 64:558-64. doi 10.1001/archneur.64.4.558

[15] Luis L, Costa J, Munoz E, de Carvalho M, Carmona S, Schneider E, Gordon CR and Valls-Sole J. Vestibulo-ocular reflex dynamics with head-impulses discriminates spinocerebellar ataxias types 1, 2 and 3 and Friedreich ataxia. Journal of vestibular research : equilibrium & orientation 2016: 26:327-34. doi 10.3233/VES-160579

[16] Buttner N, Geschwind D, Jen JC, Perlman S, Pulst SM and Baloh RW. Oculomotor phenotypes in autosomal dominant ataxias. Archives of neurology 1998: 55:1353-7. doi 10.1001/archneur.55.10.1353

[17] Crowdy KA, Hollands MA, Ferguson IT and Marple-Horvat DE. Evidence for interactive locomotor and oculomotor deficits in cerebellar patients during visually guided stepping. Experimental brain research 2000: 135:437-54. doi 10.1007/s002210000539

[18] Kim JS, Kim JS, Youn J, Seo DW, Jeong Y, Kang JH, Park JH and Cho JW. Ocular motor characteristics of different subtypes of spinocerebellar ataxia: distinguishing features. Movement disorders : official journal of the Movement Disorder Society 2013: 28:1271-7. doi 10.1002/mds.25464

[19] Bürk K, Fetter M, Skalej M, Laccone F, Stevanin G, Dichgans J and Klockgether T. Saccade velocity in idiopathic and autosomal dominant cerebellar ataxia. Journal of neurology, neurosurgery, and psychiatry 1997: 62:662-4. doi 10.1136/jnnp.62.6.662

[20] Bürk K, Abele M, Fetter M, Dichgans J, Skalej M, Laccone F, Didierjean O, Brice A and Klockgether T. Autosomal dominant cerebellar ataxia type I clinical features and MRI in families with SCA1, SCA2 and SCA3. Brain : a journal of neurology 1996: 119 ( Pt 5):1497-505. doi 10.1093/brain/119.5.1497

[21] Kerber KA, Jen JC, Perlman S and Baloh RW. Late-onset pure cerebellar ataxia: differentiating those with and without identifiable mutations. Journal of the neurological sciences 2005: 238:41-5. doi 10.1016/j.jns.2005.06.006

[22] Anastasopoulos D, Haslwanter T, Fetter M and Dichgans J. Smooth pursuit eye movements and otolith-ocular responses are differently impaired in cerebellar ataxia. Brain : a journal of neurology 1998: 121 ( Pt 8):1497-505. doi 10.1093/brain/121.8.1497

[23] Anderson JH, Christova PS, Xie TD, Schott KS, Ward K and Gomez CM. Spinocerebellar ataxia in monozygotic twins. Archives of neurology 2002: 59:1945-51. doi 10.1001/archneur.59.12.1945

[24] Federighi P, Cevenini G, Dotti MT, Rosini F, Pretegiani E, Federico A and Rufa A. Differences in saccade dynamics between spinocerebellar ataxia 2 and late-onset cerebellar ataxias. Brain : a journal of neurology 2011: 134:879-91. doi 10.1093/brain/awr009

[25] Rodríguez-Díaz JC, Velázquez-Pérez L, Rodríguez Labrada R, Aguilera Rodríguez R, Laffita Pérez D, Canales Ochoa N, Medrano Montero J, Estupiñán Rodríguez A, Osorio Borjas M, Góngora Marrero M, Reynaldo Cejas L, González Zaldivar Y and Almaguer Gotay D. Neurorehabilitation therapy in spinocerebellar ataxia type 2: A 24-week, rater-blinded, randomized, controlled trial. Movement disorders : official journal of the Movement Disorder Society 2018: 33:1481-7. doi 10.1002/mds.27437

[26] Rodríguez-Labrada R, Vázquez-Mojena Y, Canales-Ochoa N, Medrano-Montero J and Velázquez-Pérez L. Heritability of saccadic eye movements in spinocerebellar ataxia type 2: insights into an endophenotype marker. Cerebellum & ataxias 2017: 4:19. doi 10.1186/s40673-017-0078-2

[27] Rodríguez-Labrada R, Velázquez-Pérez L, Auburger G, Ziemann U, Canales-Ochoa N, Medrano-Montero J, Vázquez-Mojena Y and González-Zaldivar Y. Spinocerebellar ataxia type 2: Measures of saccade changes improve power for clinical trials. Movement disorders : official journal of the Movement Disorder Society 2016: 31:570-8. doi 10.1002/mds.26532

[28] Rufa A and Federighi P. Fast versus slow: different saccadic behavior in cerebellar ataxias. Annals of the New York Academy of Sciences 2011: 1233:148-54. doi 10.1111/j.1749-6632.2011.06126.x

[29] Saglam M and Lehnen N. Gaze stabilization in chronic vestibular-loss and in cerebellar ataxia: interactions of feedforward and sensory feedback mechanisms. Journal of vestibular research : equilibrium & orientation 2014: 24:425-31. doi 10.3233/VES-140538

[30] Velázquez-Pérez L, Rodríguez-Labrada R, Álvarez-González L, Aguilera-Rodríguez R, Álvarez Sánchez M, Canales-Ochoa N, Galicia Polo L, Haro-Valencia R, Medrano-Montero J, Vázquez-Mojena Y, Peña-Acosta A, Estupiñán-Rodríguez A and Rodríguez Pupo N. Lisuride reduces involuntary periodic leg movements in spinocerebellar ataxia type 2 patients. Cerebellum (London, England) 2012: 11:1051-6. doi 10.1007/s12311-012-0382-6

[31] Velázquez-Pérez L, Rodríguez-Chanfrau J, García-Rodríguez JC, Sánchez-Cruz G, Aguilera-Rodríguez R, Rodríguez-Labrada R, Rodríguez-Díaz JC, Canales-Ochoa N, Gotay DA, Almaguer Mederos LE, Laffita Mesa JM, Porto-Verdecia M, Triana CG, Pupo NR, Batista IH, López-Hernandez OD, Polanco ID and Novas AJ. Oral zinc sulphate supplementation for six months in SCA2 patients: a randomized, double-blind, placebo-controlled trial. Neurochemical research 2011: 36:1793-800. doi 10.1007/s11064-011-0496-0

[32] Kim JM, Nam TS, Choi SM, Kim BC and Lee SH. Clinical value of vestibulo-ocular reflex in the differentiation of spinocerebellar ataxias. Scientific reports 2023: 13:14783. doi 10.1038/s41598-023-41924-6

[33] Reetz K, Rodríguez-Labrada R, Dogan I, Mirzazade S, Romanzetti S, Schulz JB, Cruz-Rivas EM, Alvarez-Cuesta JA, Aguilera Rodríguez R, Gonzalez Zaldivar Y, Auburger G and Velázquez-Pérez L. Brain atrophy measures in preclinical and manifest spinocerebellar ataxia type 2. Annals of clinical and translational neurology 2018: 5:128-37. doi 10.1002/acn3.504

[34] Seifried C, Velázquez-Pérez L, Santos-Falcón N, Abele M, Ziemann U, Almaguer LE, Martínez-Góngora E, Sánchez-Cruz G, Canales N, Pérez-González R, Velázquez-Manresa M, Viebahn B, Stuckrad-Barre S, Klockgether T, Fetter M and Auburger G. Saccade velocity as a surrogate disease marker in spinocerebellar ataxia type 2. Annals of the New York Academy of Sciences 2005: 1039:524-7. doi 10.1196/annals.1325.059

[35] Rodriguez-Labrada R, Ortega-Sanchez R, Hernandez Casana P, Santos Morales O, Padron-Estupinan MDC, Batista-Nunez M, Jimenez Rodriguez D, Canales-Ochoa N, Pena Acosta A, Medrano Montero J, Labrada Aguilera PE, Estupinan Rodriguez A, Vazquez-Mojena Y, Almaguer Gotay D, Aymed-Garcia J, Garcia-Garcia I, Torres Vega R, Viada Gonzalez C, Valenzuela Silva CM, Silva Ricardo Y, Columbie Ximelis J, Tribin Rivero K, Valle Cabrera R, Garcia-Rodriguez JC, Crombet Ramos T, Amaro-Gonzalez D, Rodriguez-Obaya T and Velazquez-Perez L. Erythropoietin in Spinocerebellar Ataxia Type 2: Feasibility and Proof-of-Principle Issues from a Randomized Controlled Study. Movement disorders : official journal of the Movement Disorder Society 2022: 37:1516-25. doi 10.1002/mds.29045

[36] Pretegiani E, Piu P, Rosini F, Federighi P, Serchi V, Tumminelli G, Dotti MT, Federico A and Rufa A. Anti-Saccades in Cerebellar Ataxias Reveal a Contribution of the Cerebellum in Executive Functions. Frontiers in neurology 2018: 9:274. doi 10.3389/fneur.2018.00274

[37] Velázquez-Pérez L, Rodríguez-Labrada R, Cruz-Rivas EM, Fernández-Ruiz J, Vaca-Palomares I, Lilia-Campins J, Cisneros B, Peña-Acosta A, Vázquez-Mojena Y, Diaz R, Magaña-Aguirre JJ, Cruz-Mariño T, Estupiñán-Rodríguez A, Laffita-Mesa JM, González-Piña R, Canales-Ochoa N and González-Zaldivar Y. Comprehensive study of early features in spinocerebellar ataxia 2: delineating the prodromal stage of the disease. Cerebellum (London, England) 2014: 13:568-79. doi 10.1007/s12311-014-0574-3

[38] Velázquez-Pérez L, Seifried C, Abele M, Wirjatijasa F, Rodríguez-Labrada R, Santos-Falcón N, Sánchez-Cruz G, Almaguer-Mederos L, Tejeda R, Canales-Ochoa N, Fetter M, Ziemann U, Klockgether T, Medrano-Montero J, Rodríguez-Díaz J, Laffita-Mesa JM and Auburger G. Saccade velocity is reduced in presymptomatic spinocerebellar ataxia type 2. Clinical neurophysiology : official journal of the International Federation of Clinical Neurophysiology 2009: 120:632-5. doi 10.1016/j.clinph.2008.12.040

[39] Caspi A, Zivotofsky AZ and Gordon CR. Multiple saccadic abnormalities in spinocerebellar ataxia type 3 can be linked to a single deficiency in velocity feedback. Investigative ophthalmology & visual science 2013: 54:731-8. doi 10.1167/iovs.12-10689

[40] Ghasia FF, Wilmot G, Ahmed A and Shaikh AG. Strabismus and Micro-Opsoclonus in Machado-Joseph Disease. Cerebellum (London, England) 2016: 15:491-7. doi 10.1007/s12311-015-0718-0

[41] Wu C, Chen DB, Feng L, Zhou XX, Zhang JW, You HJ, Liang XL, Pei Z and Li XH. Oculomotor deficits in spinocerebellar ataxia type 3: Potential biomarkers of preclinical detection and disease progression. CNS Neurosci Ther 2017: 23:321-8. doi 10.1111/cns.12676

[42] de Oliveira CM, Leotti VB, Bolzan G, Cappelli AH, Rocha AG, Ecco G, Kersting N, Rieck M, Martins AC, Sena LS, Saraiva-Pereira ML and Jardim LB. Pre-ataxic Changes of Clinical Scales and Eye Movement in Machado-Joseph Disease: BIGPRO Study. Movement disorders : official journal of the Movement Disorder Society 2021. doi 10.1002/mds.28466

[43] Lemos J, Novo A, Duque C, Castelhano J, Eggenberger E and Januário C. "Pinball" intrusions in spinocerebellar ataxia type 3. Neurology 2018: 90:36-7. doi 10.1212/wnl.0000000000004772

[44] Gordon CR, Zivotofsky AZ and Caspi A. Impaired vestibulo-ocular reflex (VOR) in spinocerebellar ataxia type 3 (SCA3): bedside and search coil evaluation. Journal of vestibular research : equilibrium & orientation 2014: 24:351-5. doi 10.3233/ves-140527

[45] Elyoseph Z, Geisinger D, Zaltzman R, Mintz M and Gordon CR. Horizontal Vestibulo-Ocular Reflex Deficit as a Biomarker for Clinical Disease Onset, Severity, and Progression of Machado-Joseph Disease. Cerebellum (London, England) 2023. doi 10.1007/s12311-023-01552-2

[46] Geisinger D, Elyoseph Z, Zaltzman R, Mintz M and Gordon CR. Angular vestibulo ocular reflex loss with preserved saccular function in Machado-Joseph disease. Journal of the neurological sciences 2021: 424:117393. doi 10.1016/j.jns.2021.117393

[47] Bour LJ, van Rootselaar AF, Koelman JH and Tijssen MA. Oculomotor abnormalities in myoclonic tremor: a comparison with spinocerebellar ataxia type 6. Brain : a journal of neurology 2008: 131:2295-303. doi 10.1093/brain/awn177

[48] Christova P, Anderson JH and Gomez CM. Impaired eye movements in presymptomatic spinocerebellar ataxia type 6. Archives of neurology 2008: 65:530-6. doi 10.1001/archneur.65.4.530

[49] Inomata-Terada S, Fukuda H, Tokushige SI, Matsuda SI, Hamada M, Ugawa Y, Tsuji S and Terao Y. Abnormal saccade profiles in hereditary spinocerebellar degeneration reveal cerebellar contribution to visually guided saccades. Clinical neurophysiology : official journal of the International Federation of Clinical Neurophysiology 2023: 154:70-84. doi 10.1016/j.clinph.2023.07.006

[50] Lee SU, Kim JS, Kim HJ, Choi JY, Park JY, Kim JM and Yang X. Evolution of the vestibular function during head impulses in spinocerebellar ataxia type 6. Journal of neurology 2020: 267:1672-8. doi 10.1007/s00415-020-09756-w

[51] Hashimoto T, Sasaki O, Yoshida K, Takei Y and Ikeda S. Periodic alternating nystagmus and rebound nystagmus in spinocerebellar ataxia type 6. Movement disorders : official journal of the Movement Disorder Society 2003: 18:1201-4. doi 10.1002/mds.10511

[52] Gomez CM, Thompson RM, Gammack JT, Perlman SL, Dobyns WB, Truwit CL, Zee DS, Clark HB and Anderson JH. Spinocerebellar ataxia type 6: gaze-evoked and vertical nystagmus, Purkinje cell degeneration, and variable age of onset. Annals of neurology 1997: 42:933-50. doi 10.1002/ana.410420616

[53] Takeichi N, Fukushima K, Sasaki H, Yabe I, Tashiro K and Inuyama Y. Dissociation of smooth pursuit and vestibulo-ocular reflex cancellation in SCA-6. Neurology 2000: 54:860-6. doi 10.1212/wnl.54.4.860

[54] Wiest G, Tian JR, Baloh RW, Crane BT and Demer JL. Otolith function in cerebellar ataxia due to mutations in the calcium channel gene CACNA1A. Brain : a journal of neurology 2001: 124:2407-16. doi 10.1093/brain/124.12.2407

[55] Huh YE, Kim JS, Kim HJ, Park SH, Jeon BS, Kim JM, Cho JW and Zee DS. Vestibular Performance During High-Acceleration Stimuli Correlates with Clinical Decline in SCA6. Cerebellum (London, England) 2015: 14:284-91. doi 10.1007/s12311-015-0650-3

[56] Oh AK, Jacobson KM, Jen JC and Baloh RW. Slowing of voluntary and involuntary saccades: an early sign in spinocerebellar ataxia type 7. Annals of neurology 2001: 49:801-4. doi 10.1002/ana.1059

[57] Choi JH, Oh EH, Choi SY, Kim HJ, Lee SK, Choi JY, Kim JS and Choi KD. Vestibular impairments in episodic ataxia type 2. Journal of neurology 2022: 269:2687-95. doi 10.1007/s00415-021-10856-4

[58] Baloh RW, Yue Q, Furman JM and Nelson SF. Familial episodic ataxia: clinical heterogeneity in four families linked to chromosome 19p. Annals of neurology 1997: 41:8-16. doi 10.1002/ana.410410105

[59] Gordon CR, Caspi A, Levite R and Zivotofsky AZ. Mechanisms of vestibulo-ocular reflex (VOR) cancellation in spinocerebellar ataxia type 3 (SCA-3) and episodic ataxia type 2 (EA-2). Prog Brain Res 2008: 171:519-25. doi 10.1016/S0079-6123(08)00674-2

[60] Choi JH, Seo JD, Choi YR, Kim MJ, Shin JH, Kim JS and Choi KD. Exercise-induced downbeat nystagmus in a Korean family with a nonsense mutation in CACNA1A. Neurological sciences : official journal of the Italian Neurological Society and of the Italian Society of Clinical Neurophysiology 2015: 36:1393-6. doi 10.1007/s10072-015-2157-6

[61] Baloh RW, Yee RD and Boder E. Eye movements in ataxia-telangiectasia. Neurology 1978: 28:1099-104. doi 10.1212/wnl.28.11.1099

[62] Lewis RF, Lederman HM and Crawford TO. Ocular motor abnormalities in ataxia telangiectasia. Annals of neurology 1999: 46:287-95. doi 10.1002/1531-8249(199909)46:3<287::aid-ana3>3.0.co;2-0

[63] Mariani LL, Rivaud-Pechoux S, Charles P, Ewenczyk C, Meneret A, Monga BB, Fleury MC, Hainque E, Maisonobe T, Degos B, Echaniz-Laguna A, Renaud M, Wirth T, Grabli D, Brice A, Vidailhet M, Stoppa-Lyonnet D, Dubois-d'Enghien C, Le Ber I, Koenig M, Roze E, Tranchant C, Durr A, Gaymard B and Anheim M. Comparing ataxias with oculomotor apraxia: a multimodal study of AOA1, AOA2 and AT focusing on video-oculography and alpha-fetoprotein. Scientific reports 2017: 7:15284. doi 10.1038/s41598-017-15127-9

[64] Lewis RF and Crawford TO. Slow target-directed eye movements in ataxia-telangiectasia. Investigative ophthalmology & visual science 2002: 43:686-91.

[65] Mironets SA, Shurupova MA and Karelin AF. Videoocular assessment of eye movement activity in an ataxia-telangiectasia: a case study. Doc Ophthalmol 2024: 148:107-14. doi 10.1007/s10633-024-09964-z

[66] Brueggemann A, Bicvic A, Goeldlin M, Kalla R, Kerkeni H, Mantokoudis G, Abegg M, Kolnikova M, Mohaupt M and Bremova-Ertl T. Effects of Acetyl-DL-Leucine on Ataxia and Downbeat-Nystagmus in Six Patients With Ataxia Telangiectasia. J Child Neurol 2022: 37:20-7. doi 10.1177/08830738211028394

[67] Shaikh AG, Marti S, Tarnutzer AA, Palla A, Crawford TO, Straumann D, Taylor AM and Zee DS. Gaze fixation deficits and their implication in ataxia-telangiectasia. Journal of neurology, neurosurgery, and psychiatry 2009: 80:858-64. doi 10.1136/jnnp.2008.170522

[68] Shaikh AG, Marti S, Tarnutzer AA, Palla A, Crawford TO, Zee DS and Straumann D. Effects of 4-aminopyridine on nystagmus and vestibulo-ocular reflex in ataxia-telangiectasia. Journal of neurology 2013: 260:2728-35. doi 10.1007/s00415-013-7046-4

[69] Bremova T, Malinova V, Amraoui Y, Mengel E, Reinke J, Kolnikova M and Strupp M. Acetyl-dl-leucine in Niemann-Pick type C: A case series. Neurology 2015: 85:1368-75. doi 10.1212/WNL.0000000000002041

[70] Havla J, Moser M, Sztatecsny C, Lotz-Havla AS, Maier EM, Hizli B, Schinner R, Kümpfel T, Strupp M, Bremova-Ertl T and Schneider SA. Retinal axonal degeneration in Niemann-Pick type C disease. Journal of neurology 2020: 267:2070-82. doi 10.1007/s00415-020-09796-2

[71] Solomon D, Winkelman AC, Zee DS, Gray L and Büttner-Ennever J. Niemann-Pick type C disease in two affected sisters: ocular motor recordings and brain-stem neuropathology. Annals of the New York Academy of Sciences 2005: 1039:436-45. doi 10.1196/annals.1325.041

[72] Walterfang M, Abel LA, Desmond P, Fahey MC, Bowman EA and Velakoulis D. Cerebellar volume correlates with saccadic gain and ataxia in adult Niemann-Pick type C. Molecular genetics and metabolism 2013: 108:85-9. doi 10.1016/j.ymgme.2012.11.009

[73] Walterfang M, Macfarlane MD, Looi JC, Abel L, Bowman E, Fahey MC, Desmond P and Velakoulis D. Pontine-to-midbrain ratio indexes ocular-motor function and illness stage in adult Niemann-Pick disease type C. European journal of neurology 2012: 19:462-7. doi 10.1111/j.1468-1331.2011.03545.x

[74] Rottach KG, von Maydell RD, Das VE, Zivotofsky AZ, Discenna AO, Gordon JL, Landis DM and Leigh RJ. Evidence for independent feedback control of horizontal and vertical saccades from Niemann-Pick type C disease. Vision Res 1997: 37:3627-38. doi 10.1016/S0042-6989(96)00066-1

[75] Wraith JE, Vecchio D, Jacklin E, Abel L, Chadha-Boreham H, Luzy C, Giorgino R and Patterson MC. Miglustat in adult and juvenile patients with Niemann-Pick disease type C: long-term data from a clinical trial. Molecular genetics and metabolism 2010: 99:351-7. doi 10.1016/j.ymgme.2009.12.006

[76] Patterson MC, Vecchio D, Prady H, Abel L and Wraith JE. Miglustat for treatment of Niemann-Pick C disease: a randomised controlled study. Lancet Neurol 2007: 6:765-72. doi 10.1016/S1474-4422(07)70194-1

[77] Patterson MC, Vecchio D, Jacklin E, Abel L, Chadha-Boreham H, Luzy C, Giorgino R and Wraith JE. Long-term miglustat therapy in children with Niemann-Pick disease type C. J Child Neurol 2010: 25:300-5. doi 10.1177/0883073809344222

[78] Abel LA, Walterfang M, Stainer MJ, Bowman EA and Velakoulis D. Longitudinal assessment of reflexive and volitional saccades in Niemann-Pick Type C disease during treatment with miglustat. Orphanet J Rare Dis 2015: 10:160. doi 10.1186/s13023-015-0377-8

[79] Abel LA, Walterfang M, Fietz M, Bowman EA and Velakoulis D. Saccades in adult Niemann-Pick disease type C reflect frontal, brainstem, and biochemical deficits. Neurology 2009: 72:1083-6. doi 10.1212/01.wnl.0000345040.01917.9d

[80] Karaaslan Z, Hanagasi HA, Gurvit IH and Bilgic B. Video-Oculography Assessment in Neurodegenerative Ataxias and Niemann Pick Type C. Noro Psikiyatr Ars 2024: 61:101-6. doi 10.29399/npa.28563

[81] Bremova-Ertl T, Abel L, Walterfang M, Salsano E, Ardissone A, Malinova V, Kolnikova M, Gascon Bayarri J, Reza Tavasoli A, Reza Ashrafi M, Amraoui Y, Mengel E, Kolb SA, Brecht A, Bardins S and Strupp M. A cross-sectional, prospective ocular motor study in 72 patients with Niemann-Pick disease type C. European journal of neurology 2021: 28:3040-50. doi 10.1111/ene.14955

[82] Zhang H, Xiong H, Wei C, Yi M, Che Y, Zhuo J and Li X. Evaluation of the safety and efficacy of miglustat for the treatment of Chinese patients with Niemann-Pick disease type C: A prospective, open-label, single-arm, phase IV trial. Intractable Rare Dis Res 2024: 13:227-35. doi 10.5582/irdr.2024.01056

[83] Abel LA, Bowman EA, Velakoulis D, Fahey MC, Desmond P, Macfarlane MD, Looi JC, Adamson CL and Walterfang M. Saccadic eye movement characteristics in adult Niemann-Pick Type C disease: relationships with disease severity and brain structural measures. PloS one 2012: 7:e50947. doi 10.1371/journal.pone.0050947

[84] Walterfang M, Fahey M, Abel L, Fietz M, Wood A, Bowman E, Reutens D and Velakoulis D. Size and shape of the corpus callosum in adult Niemann-Pick type C reflects state and trait illness variables. AJNR Am J Neuroradiol 2011: 32:1340-6. doi 10.3174/ajnr.A2490

[85] Bowman EA, Walterfang M, Abel L, Desmond P, Fahey M and Velakoulis D. Longitudinal changes in cerebellar and subcortical volumes in adult-onset Niemann-Pick disease type C patients treated with miglustat. Journal of neurology 2015: 262:2106-14. doi 10.1007/s00415-015-7819-z

[86] Bremova T, Krafczyk S, Bardins S, Reinke J and Strupp M. Vestibular function in patients with Niemann-Pick type C disease. Journal of neurology 2016: 263:2260-70. doi 10.1007/s00415-016-8247-4

[87] Costales M, Casanueva R, Suárez V, Asensi JM, Cifuentes GA, Diñeiro M, Cadiñanos J, López F, Álvarez-Marcos C, Otero A, Gómez J, Llorente JL and Cabanillas R. CANVAS: A New Genetic Entity in the Otorhinolaryngologist's Differential Diagnosis. Otolaryngology--head and neck surgery : official journal of American Academy of Otolaryngology-Head and Neck Surgery 2021:1945998211008398. doi 10.1177/01945998211008398

[88] Borsche M, Tadic V, Konig IR, Lohmann K, Helmchen C and Bruggemann N. Head impulse testing in bilateral vestibulopathy in patients with genetically defined CANVAS. Brain Behav 2022: 12:e32546. doi 10.1002/brb3.2546

[89] Harrell RG, Cassidy AR, Klatt BN, Hovareshti P and Whitney SL. Vestibular rehabilitation in cerebellar ataxia with neuropathy and vestibular areflexia syndrome (CANVAS)- A case report. J Otol 2023: 18:199-207. doi 10.1016/j.joto.2023.06.004

[90] Pellerin D, Heindl F, Traschutz A, Rujescu D, Hartmann AM, Brais B, Houlden H, Dufke C, Riess O, Haack T, Strupp M and Synofzik M. RFC1 repeat expansions in downbeat nystagmus syndromes: frequency and phenotypic profile. Journal of neurology 2024: 271:2886-92. doi 10.1007/s00415-024-12229-z

[91] Le Ber I, Moreira MC, Rivaud-Pechoux S, Chamayou C, Ochsner F, Kuntzer T, Tardieu M, Said G, Habert MO, Demarquay G, Tannier C, Beis JM, Brice A, Koenig M and Durr A. Cerebellar ataxia with oculomotor apraxia type 1: clinical and genetic studies. Brain : a journal of neurology 2003: 126:2761-72. doi 10.1093/brain/awg283

[92] Clausi S, De Luca M, Chiricozzi FR, Tedesco AM, Casali C, Molinari M and Leggio MG. Oculomotor deficits affect neuropsychological performance in oculomotor apraxia type 2. Cortex; a journal devoted to the study of the nervous system and behavior 2013: 49:691-701. doi 10.1016/j.cortex.2012.02.007

[93] Bargagli A, Rosini F, Zanca D, Serchi V and Rufa A. Ataxia with oculomotor apraxia type 2 (AOA2): an eye movement study of two siblings. Neurological sciences : official journal of the Italian Neurological Society and of the Italian Society of Clinical Neurophysiology 2021: 42:3039-42. doi 10.1007/s10072-021-05206-1

[94] Le Ber I, Bouslam N, Rivaud-Pechoux S, Guimaraes J, Benomar A, Chamayou C, Goizet C, Moreira MC, Klur S, Yahyaoui M, Agid Y, Koenig M, Stevanin G, Brice A and Durr A. Frequency and phenotypic spectrum of ataxia with oculomotor apraxia 2: a clinical and genetic study in 18 patients. Brain : a journal of neurology 2004: 127:759-67. doi 10.1093/brain/awh080

[95] Panouilleres M, Frismand S, Sillan O, Urquizar C, Vighetto A, Pelisson D and Tilikete C. Saccades and eye-head coordination in ataxia with oculomotor apraxia type 2. Cerebellum (London, England) 2013: 12:557-67. doi 10.1007/s12311-013-0463-1

[96] McLennan YA, Mosconi MW, McKenzie FJ, Famula J, Krawchuk B, Kim K, Clark CJ, Hessl D, Rivera SM, Simon TJ, Tassone F and Hagerman RJ. Prosaccade and Antisaccade Behavior in Fragile X-Associated Tremor/Ataxia Syndrome Progression. Mov Disord Clin Pract 2022: 9:473-8. doi 10.1002/mdc3.13449

[97] Wong LM, Goodrich-Hunsaker NJ, McLennan Y, Tassone F, Zhang M, Rivera SM and Simon TJ. Eye movements reveal impaired inhibitory control in adult male fragile X premutation carriers asymptomatic for FXTAS. Neuropsychology 2014: 28:571-84. doi 10.1037/neu0000066

[98] Lasker AG, Mazzocco MM and Zee DS. Ocular motor indicators of executive dysfunction in fragile X and Turner syndromes. Brain and cognition 2007: 63:203-20. doi 10.1016/j.bandc.2006.08.002

[99] Fielding-Gebhardt H, Kelly SE, Unruh KE, Schmitt LM, Pulver SL, Khemani P and Mosconi MW. Sensorimotor and inhibitory control in aging FMR1 premutation carriers. Front Hum Neurosci 2023: 17:1271158. doi 10.3389/fnhum.2023.1271158

[100] Farzin F, Scaggs F, Hervey C, Berry-Kravis E and Hessl D. Reliability of eye tracking and pupillometry measures in individuals with fragile X syndrome. J Autism Dev Disord 2011: 41:1515-22. doi 10.1007/s10803-011-1176-2

[101] Rosini F, Pretegiani E, Mignarri A, Optican LM, Serchi V, De Stefano N, Battaglini M, Monti L, Dotti MT, Federico A and Rufa A. The role of dentate nuclei in human oculomotor control: insights from cerebrotendinous xanthomatosis. J Physiol 2017: 595:3607-20. doi 10.1113/JP273670

[102] Koens LH, Tuitert I, Blokzijl H, Engelen M, Klouwer FCC, Lange F, Leen WG, Lunsing RJ, Koelman J, Verrips A, de Koning TJ and Tijssen MAJ. Eye movement disorders in inborn errors of metabolism: A quantitative analysis of 37 patients. J Inherit Metab Dis 2022: 45:981-95. doi 10.1002/jimd.12533

[103] Pellerin D, Heindl F, Wilke C, Danzi MC, Traschutz A, Ashton C, Dicaire MJ, Cuillerier A, Del Gobbo G, Boycott KM, Claassen J, Rujescu D, Hartmann AM, Zuchner S, Brais B, Strupp M and Synofzik M. GAA-FGF14 disease: defining its frequency, molecular basis, and 4-aminopyridine response in a large downbeat nystagmus cohort. EBioMedicine 2024: 102:105076. doi 10.1016/j.ebiom.2024.105076

[104] Shirai S, Mizushima K, Fujiwara K, Koshimizu E, Matsushima M, Miyatake S, Iwata I, Yaguchi H, Matsumoto N and Yabe I. Case series: Downbeat nystagmus in SCA27B. Journal of the neurological sciences 2023: 454:120849. doi 10.1016/j.jns.2023.120849
