# Supplementary material for: Best Oculomotor Endpoints for Clinical Trials in Hereditary Ataxias: A Systematic Review and Consensus by the Ataxia Global Initiative Working Group on Digital‑Motor Biomarkers
Source: Cerebellum. 2025 Aug 13;24(5):141. doi: 10.1007/s12311-025-01894-z (PMC12350468; doi:10.1007/s12311-025-01894-z)
Supplement: Supplementary file 3 — Supplementary file3 (DOCX 253 KB) [file 12311_2025_1894_MOESM3_ESM.docx]

**Appendix 5 - Scoring sheet for assessing quality of potential OM endpoint markers**

| **Table A5-1: Scoring sheet – assessing quality of potential OM endpoint markers for hereditary ataxias**  **Disease rated: Friedreich Ataxia (FRDA)**  **Consensus ratings (PG and AAT)** | | | | | | |
| --- | --- | --- | --- | --- | --- | --- |
|  | **Oculomotor domain** | | | | | |
| **Parameter** | **Saccadic eye movements** | **Pursuit eye movements** | **Saccadic intrusions** | **Spontaneous nystagmus** | **Gaze-evoked nystagmus** | **Quantitative head-impulse test** |
| **Maximal number of studies reporting on a given parameter** | 10 (metrics) | 9 (gain and velocity) | 10 (presence and frequency of SWJ) | 4 (presence of horizontal and/or vertical SN) | 7 (presence of eccentric gaze holding) | 2 (gain of horizontal vHIT) |
| **Inclusion of carriers and/or early disease stages (0/1) *** | No | No | No | No | No | No |
| **Abnormalities detected (studies [n]) (0/1)** | - Dysmetric saccades (n=9) - Prolonged latencies (n=6) | Pursuit gain (n=5) and velocity (n=2) mildly to moderately reduced | Frequent SWJ (n=10) and ocular flutter (n=3) | DBN (n=2), PAN (n=1) | GEN (n=7), RBN (n=2) | Reduced gains (HC vHIT) (n=2) |
| **Discrimination between patients / controls (0/1)** | Significant differences in 6 studies | Significant differences in 3 studies | Significant differences in 1 study | NA | NA | Significant differences in 1 study |
| **Longitudinal data demonstrating significant changes §** | NA | NA | NA | NA | NA | NA |
| **Treatment response data demonstrating significant changes §** | NA | NA | 1 study, no significant treatment effect | NA | NA | NA |
| **Significant correlation analyses #** | VGS / AS latency with   - Scales (FARS, SLCLC)   MGS errors with   - Clinical parameters (disease duration) - Genetics (CAA repeat length) | NA | Scales (SLCLC)  Age of onset | Disease duration | NA | NA |
| **Preferred endpoint for given oculomotor domain** | **VGS / AS latency and MGS errors** | **Reduced pursuit gain** | **SWJ** | **Presence of DBN** | **Presence of GEN** | **Reduced HC aVOR gains** |
|  |  |  |  |  |  |  |
| **Overall quality rating (points)** | **4** | **2** | **4** | **2** | **1** | **2** |

* Early disease stage defined as within 2 years of symptom onset (i.e., disease duration < 2 years).

§ Rating (points): 0=no data available or data available (from at least 1 study) demonstrated no significant changes; 1= data from at least 1 study available, demonstrating significant changes.

# Rating (points): 0=no correlation analyses available or correlation analyses available being non-significant or weak; 1=significant (moderate or strong) correlation analyses in one domain (domains=clinical scores, MRI, other imaging, genetics, clinical parameters [disease duration, age at symptom onset, age, time to manifestation], questionnaires); 2=significant (moderate or strong) correlation analyses in two or more domains.

**Abbreviations**: AS=anti-saccades; aVOR=angular vestibulo-ocular reflex; DBN=downbeat nystagmus; FARS=Friedreich Ataxia Rating Scale; FRDA=Friedreich ataxia; GEN=gaze-evoked nystagmus; HC=horizontal canal; MGS=memory-guided saccades; MRI=magnetic resonance imaging; PAN=period alternating nystagmus; SLCLC=Sloan Low-Contrast Letter Chart; SN=spontaneous nystagmus; SWJ=square-wave jerks; VGS=visually-guided saccades; vHIT=video-head-impulse test.

| **Table A5-2: Scoring sheet – assessing quality of potential OM endpoint markers for hereditary ataxias**  **Disease rated: spinocerebellar ataxia type 1 (SCA1)**  **Consensus ratings (PG and AAT)** | | | | | | |
| --- | --- | --- | --- | --- | --- | --- |
|  | **Oculomotor domain** | | | | | |
| **Parameter** | **Saccadic eye movements** | **Pursuit eye movements** | **Saccadic intrusions** | **Spontaneous nystagmus** | **Gaze-evoked nystagmus** | **Quantitative head-impulse test** |
| **Maximal number of studies reporting on a given parameter** | 5 (peak velocity VGS) | 2 (pursuit gain) | 3 (presence and frequency of SWJ) | 2 (presence of SN) | 2 (presence of GEN) | 1 (aVOR gains) |
| **Inclusion of carriers and/or early disease stages (0/1) *** | No | No | No | No | No | No |
| **Abnormalities detected (studies [n]) (0/1)** | - Mildly to moderately reduced velocity (n=5) - Normal to increased latency (n=2) - Mild dysmetria (n=3) | Mildly reduced pursuit gain (n=3) | Frequent SWJ (n=3) | None | GEN frequently identified (n=2) | None |
| **Discrimination between patients / controls (0/1)** | Significant differences in 5 studies | Significant differences in 3 studies | Significant differences in 2 studies | None | Significant differences in 1 study | None |
| **Longitudinal data demonstrating significant changes §** | None | None | None | None | None | None |
| **Treatment response data demonstrating significant changes §** | None | None | None | None | None | None |
| **Significant correlation analyses #** | None | None | None | None | None | None |
| **Preferred endpoint for given oculomotor domain** | **Peak velocity VGS** | **Pursuit gain** | **Presence of SWJ** | None | **Presence of GEN** | None |
|  |  |  |  |  |  |  |
| **Overall quality rating (points)** | **2** | **2** | **2** | **0** | **2** | **0** |

* Early disease stage defined as within 2 years of symptom onset (i.e., disease duration < 2 years).

§ Rating (points): 0=no data available or data available (from at least 1 study) demonstrated no significant changes; 1= data from at least 1 study available, demonstrating significant changes.

# Rating (points): 0=no correlation analyses available or correlation analyses available being non-significant or weak; 1=significant (moderate or strong) correlation analyses in one domain (domains=clinical scores, MRI, other imaging, genetics, clinical parameters [disease duration, age at symptom onset, age, time to manifestation], questionnaires); 2=significant (moderate or strong) correlation analyses in two or more domains.

**Abbreviations**: aVOR=angular vestibulo-ocular reflex; DBN=downbeat nystagmus; GEN=gaze-evoked nystagmus; HC=horizontal canal; MGS=memory-guided saccades; MRI=magnetic resonance imaging; SN=spontaneous nystagmus; SWJ=square-wave jerks; VGS=visually-guided saccades; vHIT=video-head-impulse test.

| **Table A5-3: Scoring sheet – assessing quality of potential OM endpoint markers for hereditary ataxias**  **Disease rated: spinocerebellar ataxia type 2 (SCA2)**  **Consensus ratings (PG and AAT)** | | | | | | |
| --- | --- | --- | --- | --- | --- | --- |
|  | **Oculomotor domain** | | | | | |
| **Parameter** | **Saccadic eye movements** | **Pursuit eye movements** | **Saccadic intrusions** | **Spontaneous nystagmus** | **Gaze-evoked nystagmus** | **Quantitative head-impulse test** |
| **Maximal number of studies reporting on a given parameter** | 15 (peak velocity for VGS) | 3 (pursuit gain) | 4 (presence and frequency of SWJ) | 3 (presence of SN) | 3 (presence of GEN and RBN) | 2 (horizontal or horizontal and vertical aVOR gains) |
| **Inclusion of carriers and/or early disease stages (0/1) *** | Preclinical carriers | None | None | None | None | None |
| **Abnormalities detected (studies [n]) (0/1)** | - Severely reduced saccadic velocity in symptomatic patients (n=14) and carriers (n=2). - Saccades dysmetric in some studies (n=5) - Increased saccadic latency (n=3) | Normal to mildly reduced gain (n=3) | Increased frequency of SWJ (n=4) | None | GEN present in part of patients (n=2) | None |
| **Discrimination between patients / controls (0/1)** | Significant differences in 11 studies | Significant differences in 1 study | Significant differences in 3 studies | None | Significant differences in 1 study | None |
| **Longitudinal data demonstrating significant changes §** | - Peak velocity VGS and accuracy significantly decreased and latency significantly increased over 60 months - No significant changes in Pvel, accuracy and latency over shorter (12 month) period | None | None | None | None | None |
| **Treatment response data demonstrating significant changes §** | - Saccade latencies reduced after treatment with zinc sulfate - Saccadic latencies decreased significantly with NeuroEPO treatment | None | None | None | None | None |
| **Significant correlation analyses #** | Saccadic velocity with   - Scales (SARA, ICARS) - Clinical parameters (age at onset, time to sx onset, disease duration, age) - MRI - Genetics (CAG repeat length) |  |  |  |  |  |
| **Preferred endpoint for given oculomotor domain** | **Peak velocity VGS** | **Pursuit gain** | **Presence of SWJ** | **None** | **Presence of GEN** | **none** |
|  |  |  |  |  |  |  |
| **Overall quality rating (points)** | **7** | **2** | **2** | **0** | **2** | **0** |

* Early disease stage defined as within 2 years of symptom onset (i.e., disease duration < 2 years).

§ Rating (points): 0=no data available or data available (from at least 1 study) demonstrated no significant changes; 1= data from at least 1 study available, demonstrating significant changes.

# Rating (points): 0=no correlation analyses available or correlation analyses available being non-significant or weak; 1=significant (moderate or strong) correlation analyses in one domain (domains=clinical scores, MRI, other imaging, genetics, clinical parameters [disease duration, age at symptom onset, age, time to manifestation], questionnaires); 2=significant (moderate or strong) correlation analyses in two or more domains.

**Abbreviations**: AS=anti-saccades; aVOR=angular vestibulo-ocular reflex; DBN=downbeat nystagmus; GEN=gaze-evoked nystagmus; HC=horizontal canal; ICARS=International Cooperative Ataxia Rating Scale; MGS=memory-guided saccades; MRI=magnetic resonance imaging; RBN=rebound nystagmus; SARA=Scale for the Assessment and Rating of Ataxia; SN=spontaneous nystagmus; SWJ=square-wave jerks; VGS=visually-guided saccades; vHIT=video-head-impulse test.

| **Table A5-4: Scoring sheet – assessing quality of potential OM endpoint markers for hereditary ataxias**  **Disease rated: spinocerebellar ataxia type 3 (SCA3)**  **Consensus ratings (PG and AAT)** | | | | | | |
| --- | --- | --- | --- | --- | --- | --- |
|  | **Oculomotor domain** | | | | | |
| **Parameter** | **Saccadic eye movements** | **Pursuit eye movements** | **Saccadic intrusions** | **Spontaneous nystagmus** | **Gaze-evoked nystagmus** | **Quantitative head-impulse test** |
| **Maximal number of studies reporting on a given parameter** | 10 (peak velocity for VGS) | 5 (pursuit gain) | 5 (presence and frequency of SWJ) | 3 (presence of SN) | 7 (presence of GEN) | 6 (horizontal or horizontal and vertical aVOR gains) |
| **Inclusion of carriers and/or early disease stages (0/1) *** | Preclinical carriers | Preclinical carriers | Preclinical carriers | Preclinical carriers | Preclinical carriers | Preclinical carriers |
| **Abnormalities detected (studies [n]) (0/1)** | - Saccade velocity mildly reduced in some studies (n=6) - Dysmetric (n=3) or overshooting saccades (n=3) - Increased AS error rate (n=1) | Reduced pursuit gain (n=4) | Increased frequency of SWJ (n=5) | Mild SN in some patients (n=2) | Frequently present GEN (n=6) | - Reduced horizontal vHIT gains (n=6) - Reduced horizontal and vertical vHIT gains (n=2) |
| **Discrimination between patients / controls (0/1)** | Significant differences in 4 studies | Significant differences in 3 studies | Significant differences in 4 studies | None | Significant differences in 6 studies | Significant differences in 6 studies |
| **Longitudinal data demonstrating significant changes §** | None | None | None | None | None | Horizontal VOR gain decreased significantly between first and second examination |
| **Treatment response data demonstrating significant changes §** | None | None | None | None | None | None |
| **Significant correlation analyses #** | Saccadic velocity with   - Scales (SARA, ICARS, NESSCA, INAScount, SCAFI, CCFS) - Clinical parameters (time to sx onset, disease duration)   Saccadic latency with   - Scales (SARA) - Clinical parameters (disease duration)   Antisaccadic error rate with   - Scales (SARA) - Clinical parameters (disease duration) - Genetics (CAG repeat length) | Horizontal pursuit gain with   - Scales (SARA) - Clinical parameters (disease duration) | SWJ frequency and amplitude with   - Scales (SARA) | SPV SN with   - ICARS / NESSCA / SCAFI / CCFS | GEN with   - Scales (SARA, ICARS, NESSCA, INAScount, SCAFI, CCFS) - Clinical parameters (time to sx onset, disease duration) | HC vHIT gain with   - Scales (SARA, SARA change over time, ICARS, NESSCA, INAScount, SCAFI, CCFS) - Clinical parameters (time to sx onset) - Genetics (CAG repeat length) |
| **Preferred endpoint for given oculomotor domain** | VGS peak vel and metrics | Pursuit gain | Frequency of SWJ | SPV SN | GEN | HC vHIT gains |
|  |  |  |  |  |  |  |
| **Overall quality rating (points)** | **5** | **5** | **4** | **3** | **5** | **6** |

* Early disease stage defined as within 2 years of symptom onset (i.e., disease duration < 2 years).

§ Rating (points): 0=no data available or data available (from at least 1 study) demonstrated no significant changes; 1= data from at least 1 study available, demonstrating significant changes.

# Rating (points): 0=no correlation analyses available or correlation analyses available being non-significant or weak; 1=significant (moderate or strong) correlation analyses in one domain (domains=clinical scores, MRI, other imaging, genetics, clinical parameters [disease duration, age at symptom onset, age, time to manifestation], questionnaires); 2=significant (moderate or strong) correlation analyses in two or more domains.

**Abbreviations**: AS=anti-saccades; aVOR=angular vestibulo-ocular reflex; CCFS=Composite Cerebellar Functional Score; DBN=downbeat nystagmus; GEN=gaze-evoked nystagmus; HC=horizontal canal; ICARS=International Cooperative Ataxia Rating Scale; INAScount=Inventory of Non-Ataxic Signs; MGS=memory-guided saccades; MRI=magnetic resonance imaging; NESSCA=Neurological Examination Score for Spinocerebellar Ataxia; RBN=rebound nystagmus; SARA=Scale for the Assessment and Rating of Ataxia; SN=spontaneous nystagmus; SWJ=square-wave jerks; VGS=visually-guided saccades; vHIT=video-head-impulse test.

| **Table A5-5: Scoring sheet – assessing quality of potential OM endpoint markers for hereditary ataxias**  **Disease rated: spinocerebellar ataxia type 6 (SCA6)**  **Consensus ratings (PG and AAT)** | | | | | | |
| --- | --- | --- | --- | --- | --- | --- |
|  | **Oculomotor domain** | | | | | |
| **Parameter** | **Saccadic eye movements** | **Pursuit eye movements** | **Saccadic intrusions** | **Spontaneous nystagmus** | **Gaze-evoked nystagmus** | **Quantitative head-impulse test** |
| **Maximal number of studies reporting on a given parameter** | 7 (metrics and peak velocity for VGS) | 9 (pursuit gain) | 5 (presence and frequency of SWJ) | 7 (presence of SN) | 7 (presence of GEN and RBN) | 3 (horizontal or horizontal and vertical aVOR gains) |
| **Inclusion of carriers and/or early disease stages (0/1) *** | Preclinical carriers | Preclinical carriers | Preclinical carriers | Preclinical carriers | Preclinical carriers | none |
| **Abnormalities detected (studies [n]) (0/1)** | - Reduced gain (n=2) - Mildly reduced peak velocity (n=2) - Dysmetric saccades (n=6) | Substantially reduced pursuit gain (n=9) | Increased number of SWJ (n=3) | Downbeat Nystagmus in most patients (n=6) | GEN (n=7) and RBN (n=3) in some patients | - Reduced (n=2) or increased (n=1) HC vHIT gains - Increased AC vHIT gains (n=1) - Reduced (n=2) or increased (n=1) PC vHIT gains |
| **Discrimination between patients / controls (0/1)** | Significant differences in 6 studies | Significant differences in 8 studies | Significant differences in 2 studies | Significant differences in 5 studies | Significant differences in 5 studies | Significant differences in 3 studies |
| **Longitudinal data demonstrating significant changes §** | No change in SEM over time in pre-symptomatic carriers | No change in PEM over time in pre-symptomatic carriers | No change in SI over time in pre-symptomatic carriers | No change in SN over time in pre-symptomatic carriers | No change in GEN over time in pre-symptomatic carriers | Significant decrease in HC and AC vHIT gains over time, but not for PC vHIT gains |
| **Treatment response data demonstrating significant changes §** | None | None | None | None | None | None |
| **Significant correlation analyses #** | None | None | None | None | None | HC vHIT gains with   - ICARS, SARA   AC and PC vHIT gains with   - SARA |
| **Preferred endpoint for given oculomotor domain** | Peak velocity and metrics | Pursuit gain | Frequency of SWJ | DBN | GEN | HC and VC vHIT gains |
|  |  |  |  |  |  |  |
| **Overall quality rating (points)** | **3** | **3** | **3** | **3** | **3** | **4** |

* Early disease stage defined as within 2 years of symptom onset (i.e., disease duration < 2 years).

§ Rating (points): 0=no data available or data available (from at least 1 study) demonstrated no significant changes; 1= data from at least 1 study available, demonstrating significant changes.

# Rating (points): 0=no correlation analyses available or correlation analyses available being non-significant or weak; 1=significant (moderate or strong) correlation analyses in one domain (domains=clinical scores, MRI, other imaging, genetics, clinical parameters [disease duration, age at symptom onset, age, time to manifestation], questionnaires); 2=significant (moderate or strong) correlation analyses in two or more domains.

**Abbreviations**: aVOR=angular vestibulo-ocular reflex; DBN=downbeat nystagmus; GEN=gaze-evoked nystagmus; HC=horizontal canal; MRI=magnetic resonance imaging; PEM=pursuit eye movements; RBN=rebound nystagmus; SEM=saccadic eye movements; SN=spontaneous nystagmus; SWJ=square-wave jerks; VC=vertical canal; VGS=visually-guided saccades; vHIT=video-head-impulse test.

| **Table A5-6: Scoring sheet – assessing quality of potential OM endpoint markers for hereditary ataxias**  **Disease rated: spinocerebellar ataxia type 7 (SCA7)**  **Consensus ratings (PG and AAT)** | | | | | | |
| --- | --- | --- | --- | --- | --- | --- |
|  | **Oculomotor domain** | | | | | |
| **Parameter** | **Saccadic eye movements** | **Pursuit eye movements** | **Saccadic intrusions** | **Spontaneous nystagmus** | **Gaze-evoked nystagmus** | **Quantitative head-impulse test** |
| **Maximal number of studies reporting on a given parameter** | 2 (peak velocity for VGS) | 2 (pursuit gain) | 1 (presence of SWJ) | 1 (presence of SN) | 1 (presence of GEN) | 1 (horizontal and vertical aVOR gains) |
| **Inclusion of carriers and/or early disease stages (0/1) *** | None | None | None | None | None | None |
| **Abnormalities detected (studies [n]) (0/1)** | - Increased latency (n=1) - Reduced peak velocity (n=2) - Dysmetric saccades (n=1) | Substantially reduced pursuit gain (n=2) | None | None | None | - Reduced VC vHIT gains (n=1) |
| **Discrimination between patients / controls (0/1)** | Significant differences in 1 study | Significant differences in 1 study | None | None | None | Significant differences in 1 study |
| **Longitudinal data demonstrating significant changes §** | None | None | None | None | None | None |
| **Treatment response data demonstrating significant changes §** | None | None | None | None | None | None |
| **Significant correlation analyses #** | None | None | None | None | None | None |
| **Preferred endpoint for given oculomotor domain** | Peak velocity | Pursuit gain | None | None | None | HC and VC vHIT gains |
|  |  |  |  |  |  |  |
| **Overall quality rating (points)** | **2** | **2** | **0** | **0** | **0** | **2** |

* Early disease stage defined as within 2 years of symptom onset (i.e., disease duration < 2 years).

§ Rating (points): 0=no data available or data available (from at least 1 study) demonstrated no significant changes; 1= data from at least 1 study available, demonstrating significant changes.

# Rating (points): 0=no correlation analyses available or correlation analyses available being non-significant or weak; 1=significant (moderate or strong) correlation analyses in one domain (domains=clinical scores, MRI, other imaging, genetics, clinical parameters [disease duration, age at symptom onset, age, time to manifestation], questionnaires); 2=significant (moderate or strong) correlation analyses in two or more domains.

| **Table A5-7: Scoring sheet – assessing quality of potential OM endpoint markers for hereditary ataxias**  **Disease rated: Episodic ataxia type 2 (EA2)**  **Consensus ratings (PG and AAT)** | | | | | | |
| --- | --- | --- | --- | --- | --- | --- |
|  | **Oculomotor domain** | | | | | |
| **Parameter** | **Saccadic eye movements** | **Pursuit eye movements** | **Saccadic intrusions** | **Spontaneous nystagmus** | **Gaze-evoked nystagmus** | **Quantitative head-impulse test** |
| **Maximal number of studies reporting on a given parameter** | 3 (metrics and peak velocity for VGS) | 5 (pursuit gain) | NA | 3 (presence of SN) | 2 (presence of GEN) | 2 (horizontal and vertical aVOR gains) |
| **Inclusion of carriers and/or early disease stages (0/1) *** | None | None | NA | None | None | None |
| **Abnormalities detected (studies [n]) (0/1)** | - Reduced peak velocity (n=1) - Dysmetric saccades (n=2) | - Pursuit gain significantly reduced (n=5) | NA | Downbeat nystagmus (n=3) | Presence of GEN and RBN (n=2) | HC and/or VC vHIT gain mildly reduced or normal (n=2) |
| **Discrimination between patients / controls (0/1)** | None | Significant differences in 2 studies | NA | None | None | Significant differences in 1 study |
| **Longitudinal data demonstrating significant changes §** | None | None | NA | None | None | None |
| **Treatment response data demonstrating significant changes §** | None | None | NA | None | None | None |
| **Significant correlation analyses #** | None | None | NA | None | None | None |
| **Preferred endpoint for given oculomotor domain** | Saccade metrics | Pursuit gain | NA | Downbeat nystagmus | GEN | None |
|  |  |  |  |  |  |  |
| **Overall quality rating (points)** | **1** | **2** | NA | **1** | **1** | **2** |

* Early disease stage defined as within 2 years of symptom onset (i.e., disease duration < 2 years).

§ Rating (points): 0=no data available or data available (from at least 1 study) demonstrated no significant changes; 1= data from at least 1 study available, demonstrating significant changes.

# Rating (points): 0=no correlation analyses available or correlation analyses available being non-significant or weak; 1=significant (moderate or strong) correlation analyses in one domain (domains=clinical scores, MRI, other imaging, genetics, clinical parameters [disease duration, age at symptom onset, age, time to manifestation], questionnaires); 2=significant (moderate or strong) correlation analyses in two or more domains.

**Abbreviations**: aVOR=angular vestibulo-ocular reflex; DBN=downbeat nystagmus; GEN=gaze-evoked nystagmus; HC=horizontal canal; MRI=magnetic resonance imaging; NPC=Niemann-Pick disease type C; PEM=pursuit eye movements; RBN=rebound nystagmus; SEM=saccadic eye movements; SN=spontaneous nystagmus; SWJ=square-wave jerks; VGS=visually-guided saccades.

| **Table A5-8: Scoring sheet – assessing quality of potential OM endpoint markers for hereditary ataxias**  **Disease rated: Ataxia telangiectasia (A-T)**  **Consensus ratings (PG and AAT)** | | | | | | |
| --- | --- | --- | --- | --- | --- | --- |
|  | **Oculomotor domain** | | | | | |
| **Parameter** | **Saccadic eye movements** | **Pursuit eye movements** | **Saccadic intrusions** | **Spontaneous nystagmus** | **Gaze-evoked nystagmus** | **Quantitative head-impulse test** |
| **Maximal number of studies reporting on a given parameter** | 4 (latency and metrics of VGS) | 5 (pursuit gain) | 3 (presence, amplitude and frequency of SWJ) | 5 (presence of SN) | 4 (presence of GEN) | 1 (aVOR gains) |
| **Inclusion of carriers and/or early disease stages (0/1) *** | None | None | None | None | None | None |
| **Abnormalities detected (studies [n]) (0/1)** | - Hypometric saccades (n=3) - Hypermetric saccades (n=1) - Normal to increased (n=2) latency | Reduced pursuit gain (n=4) | Saccadic intrusions (SWJ, OF) in most patients (n=3) | - Horizontal-torsional (n=2) or DBN (n=3) - PAN (n=2) | Horizontal GEN (n=1) | Normal to increased vHIT gains (n=1) |
| **Discrimination between patients / controls (0/1)** | Significant differences in 4 studies | Significant differences in 2 studies | Significant differences in 1 study | Significant differences in 1 study | Significant differences in 1 study | None |
| **Longitudinal data demonstrating significant changes §** | None | None | None | None | None | None |
| **Treatment response data demonstrating significant changes §** | None | None | None | - PAN and SN SPV decreased after treatment with 4-AP - DBN SPV decreased after treatment with acetyl-DL-leucine | None | None |
| **Significant correlation analyses #** | None | Pursuit gain with   - Scores (A-T index) - Clinical parameters (age) | None | None | None | None |
| **Preferred endpoint for given oculomotor domain** | VGS hypometria | Reduced pursuit gain | Presence of SI | Presence of DBN | Presence of GEN | None |
|  |  |  |  |  |  |  |
| **Overall quality rating (points)** | **2** | **4** | **2** | **3** | **2** | **1** |

* Early disease stage defined as within 2 years of symptom onset (i.e., disease duration < 2 years).

§ Rating (points): 0=no data available or data available (from at least 1 study) demonstrated no significant changes; 1= data from at least 1 study available, demonstrating significant changes.

# Rating (points): 0=no correlation analyses available or correlation analyses available being non-significant or weak; 1=significant (moderate or strong) correlation analyses in one domain (domains=clinical scores, MRI, other imaging, genetics, clinical parameters [disease duration, age at symptom onset, age, time to manifestation], questionnaires); 2=significant (moderate or strong) correlation analyses in two or more domains.

**Abbreviations**: 4-AP=4-aminopyridine; A-T=ataxia telangiectasia; aVOR=angular vestibulo-ocular reflex; DBN=downbeat nystagmus; GEN=gaze-evoked nystagmus; HC=horizontal canal; MRI=magnetic resonance imaging; OF=ocular flutter; PAN=periodic alternating nystagmus; PEM=pursuit eye movements; RBN=rebound nystagmus; SEM=saccadic eye movements; SN=spontaneous nystagmus; SI=saccadic intrusions; SPV=slow-phase velocity; SWJ=square-wave jerks; VGS=visually-guided saccades; vHIT=video-head-impulse test.

| **Table A5-9: Scoring sheet – assessing quality of potential OM endpoint markers for hereditary ataxias**  **Disease rated: Niemann-Pick disease type C (NPC)**  **Consensus ratings (PG and AAT)** | | | | | | |
| --- | --- | --- | --- | --- | --- | --- |
|  | **Oculomotor domain** | | | | | |
| **Parameter** | **Saccadic eye movements** | **Pursuit eye movements** | **Saccadic intrusions** | **Spontaneous nystagmus** | **Gaze-evoked nystagmus** | **Quantitative head-impulse test** |
| **Maximal number of studies reporting on a given parameter** | 17 (peak velocity VGS) | 3 (pursuit gain) | None | None | 2 (presence of GEN) | 1 (horizontal aVOR gains) |
| **Inclusion of carriers and/or early disease stages (0/1) *** | None | None | NA | NA | None | None |
| **Abnormalities detected (studies [n]) (0/1)** | Marked slowing of vertical saccades (n=6) | Pursuit gain preserved (n=1) or reduced (n=2) | NA | NA | None | None |
| **Discrimination between patients / controls (0/1)** | Significant differences in 7 studies | None | NA | NA | None | None |
| **Longitudinal data demonstrating significant changes §** | None | None | NA | NA | None | None |
| **Treatment response data demonstrating significant changes §** | - No changes of SEM after 4 weeks of acetyl-DL-leucine - Improvement of VGS velocity and PV after 12 months of miglustat treatment, stabilization after 24 months of treatment | No changes of PEM after 4 weeks of acetyl-DL-leucine | NA | NA | no changes of GEN after 4 weeks of acetyl-DL-leucine | None |
| **Significant correlation analyses #** | VGS gain with   - MRI and other imaging - Disease severity - Disease duration   Horizontal VGS PV with   - Disease severity - Disease duration   Horizontal VGS latency with   - Disease severity   Anti-saccade error rate   - Disease duration | NA | NA | NA | None | None |
| **Preferred endpoint for given oculomotor domain** | Vertical VGS peak velocity reduced | Pursuit gain reduction | NA | NA | None | None |
|  |  |  |  |  |  |  |
| **Overall quality rating (points)** | **5** | **1** | NA | NA | **0** | **0** |

* Early disease stage defined as within 2 years of symptom onset (i.e., disease duration < 2 years).

§ Rating (points): 0=no data available or data available (from at least 1 study) demonstrated no significant changes; 1= data from at least 1 study available, demonstrating significant changes.

# Rating (points): 0=no correlation analyses available or correlation analyses available being non-significant or weak; 1=significant (moderate or strong) correlation analyses in one domain (domains=clinical scores, MRI, other imaging, genetics, clinical parameters [disease duration, age at symptom onset, age, time to manifestation], questionnaires); 2=significant (moderate or strong) correlation analyses in two or more domains.

**Abbreviations**: aVOR=angular vestibulo-ocular reflex; DBN=downbeat nystagmus; GEN=gaze-evoked nystagmus; HC=horizontal canal; MRI=magnetic resonance imaging; NPC=Niemann-Pick disease type C; PEM=pursuit eye movements; RBN=rebound nystagmus; SEM=saccadic eye movements; SN=spontaneous nystagmus; SWJ=square-wave jerks; VGS=visually-guided saccades.

| **Table A5-10: Scoring sheet – assessing quality of potential OM endpoint markers for hereditary ataxias**  **Disease rated: RFC1-related ataxia**  **Consensus ratings (PG and AAT)** | | | | | | |
| --- | --- | --- | --- | --- | --- | --- |
|  | **Oculomotor domain** | | | | | |
| **Parameter** | **Saccadic eye movements** | **Pursuit eye movements** | **Saccadic intrusions** | **Spontaneous nystagmus** | **Gaze-evoked nystagmus** | **Quantitative head-impulse test** |
| **Maximal number of studies reporting on a given parameter** | None | None | None | 1 (presence of SN) | None | 4 (aVOR gains) |
| **Inclusion of carriers and/or early disease stages (0/1) *** | NA | NA | NA | None | NA | None |
| **Abnormalities detected (studies [n]) (0/1)** | NA | NA | NA | Downbeat nystagmus (n=1) | NA | Bilaterally reduced HC vHIT gains (n=4) |
| **Discrimination between patients / controls (0/1)** | NA | NA | NA | None | NA | Significant differences in 4 studies |
| **Longitudinal data demonstrating significant changes §** | NA | NA | NA | None | NA | None |
| **Treatment response data demonstrating significant changes §** | NA | NA | NA | None | NA | None |
| **Significant correlation analyses #** | NA | NA | NA | None | NA | HC vHIT gain with   - Clinical parameters (disease duration) |
| **Preferred endpoint for given oculomotor domain** | NA | NA | NA | DBN | NA | Bilateral HC vHIT gain |
|  |  |  |  |  |  |  |
| **Overall quality rating (points)** | NA | NA | NA | 1 | NA | 3 |

* Early disease stage defined as within 2 years of symptom onset (i.e., disease duration < 2 years).

§ Rating (points): 0=no data available or data available (from at least 1 study) demonstrated no significant changes; 1= data from at least 1 study available, demonstrating significant changes.

# Rating (points): 0=no correlation analyses available or correlation analyses available being non-significant or weak; 1=significant (moderate or strong) correlation analyses in one domain (domains=clinical scores, MRI, other imaging, genetics, clinical parameters [disease duration, age at symptom onset, age, time to manifestation], questionnaires); 2=significant (moderate or strong) correlation analyses in two or more domains.

**Abbreviations**: aVOR=angular vestibulo-ocular reflex; DBN=downbeat nystagmus; GEN=gaze-evoked nystagmus; HC=horizontal canal; MRI=magnetic resonance imaging; RFC1=replication factor C subunit 1; SN=spontaneous nystagmus; vHIT=video-head-impulse test.

| **Table A5-11: Scoring sheet – assessing quality of potential OM endpoint markers for hereditary ataxias**  **Disease rated: ataxia with oculomotor apraxia type 1 (AOA1)**  **Consensus ratings (PG and AAT)** | | | | | | |
| --- | --- | --- | --- | --- | --- | --- |
|  | **Oculomotor domain** | | | | | |
| **Parameter** | **Saccadic eye movements** | **Pursuit eye movements** | **Saccadic intrusions** | **Spontaneous nystagmus** | **Gaze-evoked nystagmus** | **Quantitative head-impulse test** |
| **Maximal number of studies reporting on a given parameter** | 2 (latency VGS) | 1 (gain) | 1 (presence and frequency of SWJ) | 1 (presence of horizontal SN) | 1 (presence of eccentric gaze holding deficits) | 0 |
| **Inclusion of carriers and/or early disease stages (0/1) *** | No | No | No | No | No | NaN |
| **Abnormalities detected (studies [n]) (0/1)** | - Severe dysmetria (n=2) - Moderately decreased velocity (n=1) - Normal to increased latency (n=1) - Increased AS error rate (n=2) | None | Increased number of SWJ in some patients (n=1) | DBN in some patients (n=1) | Horizontal GEN in some patients (n=1) | NaN |
| **Discrimination between patients / controls (0/1)** | Significant differences in 2 studies | Assessed in 1 study, no significant differences | None | None | Significant differences in 1 study | NaN |
| **Longitudinal data demonstrating significant changes §** | None | None | None | None | None | NaN |
| **Treatment response data demonstrating significant changes §** | None | None | None | None | None | NaN |
| **Significant correlation analyses #** | None | None | None | None | None | NaN |
| **Preferred endpoint for given oculomotor domain** | **VGS metrics and AS error rate** |  |  |  | **Horizontal GEN** | NaN |
|  |  |  |  |  |  |  |
| **Overall quality rating (points)** | **2** | **0** | **1** | **1** | **2** | NaN |

* Early disease stage defined as within 2 years of symptom onset (i.e., disease duration < 2 years).

§ Rating (points): 0=no data available or data available (from at least 1 study) demonstrated no significant changes; 1= data from at least 1 study available, demonstrating significant changes.

# Rating (points): 0=no correlation analyses available or correlation analyses available being non-significant or weak; 1=significant (moderate or strong) correlation analyses in one domain (domains=clinical scores, MRI, other imaging, genetics, clinical parameters [disease duration, age at symptom onset, age, time to manifestation], questionnaires); 2=significant (moderate or strong) correlation analyses in two or more domains.

**Abbreviations**: AOA=ataxia with oculomotor apraxia; AS=anti-saccades; DBN=downbeat nystagmus; GEN=gaze-evoked nystagmus; MRI=magnetic resonance imaging; RBN=rebound nystagmus; SN=spontaneous nystagmus; SWJ=square-wave jerks; VGS=visually-guided saccades.

Abbreviations: AS=anti-saccades; aVOR=angular vestibulo-ocular reflex; FARS=Friedreich Ataxia Rating Scale; FRDA=Friedreich ataxia; GEN=gaze-evoked nystagmus; ICARS=International Cooperative Ataxia Rating Scale; MGS=memory-guided saccades; NA=not available; NPC=Niemann-Pick disease type C; OM=oculomotor; qHIT=quantitative head-impulse test; RBN=rebound nystagmus; SARA=Scale for the Assessment and Rating of Ataxia; SCA=spinocerebellar ataxia; *SCAFI=SCA Functional Index;* SEM=saccadic eye movements; SI=saccadic intrusions; SLCLC=Sloan Low-Contrast Letter Chart; SN=spontaneous nystagmus; PEM=pursuit eye movements; SCCs=semicircular canals; SWJ=square-wave jerks; VGS=visually-guided saccades.

| **Table A5-12: Scoring sheet – assessing quality of potential OM endpoint markers for hereditary ataxias**  **Disease rated: ataxia with oculomotor apraxia type 2 (AOA2)**  **Consensus ratings (PG and AAT)** | | | | | | |
| --- | --- | --- | --- | --- | --- | --- |
|  | **Oculomotor domain** | | | | | |
| **Parameter** | **Saccadic eye movements** | **Pursuit eye movements** | **Saccadic intrusions** | **Spontaneous nystagmus** | **Gaze-evoked nystagmus** | **Quantitative head-impulse test** |
| **Maximal number of studies reporting on a given parameter** | 5 (latency VGS) | 1 (pursuit gain) | 2 (presence and frequency of SWJ) | 1 (presence of SN) | 1 (presence of GEN) | 0 |
| **Inclusion of carriers and/or early disease stages (0/1) *** | No | No | No | No | No | NaN |
| **Abnormalities detected (studies [n]) (0/1)** | - Mild to moderate VGS dysmetria (n=4) - Normal to moderately decreased VGS velocity (n=4) - Normal to mildly increased VGS latencies (n=3) - Strongly increased AS error rate (n=4) - Increased AS latency (n=1) - Increased MGS latency (n=1) - Dysmetric MGS (n=1) | None | SWJ in some patients (n=2) | DBN in some patients (n=2) | Horizontal GEN in most patients (n=1) | NaN |
| **Discrimination between patients / controls (0/1)** | Assessed in 5 studies, significant differences in 5 | Assessed in 1 study, no significant differences | Assessed in 2 studies, significant differences in 2 | Assessed in 1 study, significant differences in 1 | Assessed in 1 study, significant differences in 1 | NaN |
| **Longitudinal data demonstrating significant changes §** | None | None | None | None | None | NaN |
| **Treatment response data demonstrating significant changes §** | None | None | None | None | None | NaN |
| **Significant correlation analyses #** | None | None | None | None | None | NaN |
| **Preferred endpoint for given oculomotor domain** | **AS error rate and VGS metrics / velocity** |  |  |  | **Horizontal GEN** | NaN |
|  |  |  |  |  |  | NaN |
| **Overall quality rating (points)** | **2** | **0** | **2** | **2** | **2** | NaN |

* Early disease stage defined as within 2 years of symptom onset (i.e., disease duration < 2 years).

§ Rating (points): 0=no data available or data available (from at least 1 study) demonstrated no significant changes; 1= data from at least 1 study available, demonstrating significant changes.

# Rating (points): 0=no correlation analyses available or correlation analyses available being non-significant or weak; 1=significant (moderate or strong) correlation analyses in one domain (domains=clinical scores, MRI, other imaging, genetics, clinical parameters [disease duration, age at symptom onset, age, time to manifestation], questionnaires); 2=significant (moderate or strong) correlation analyses in two or more domains.

**Abbreviations**: AOA=ataxia with oculomotor apraxia; AS=anti-saccades; DBN=downbeat nystagmus; GEN=gaze-evoked nystagmus; MGS=memory-guided saccades; MRI=magnetic resonance imaging; RBN=rebound nystagmus; SN=spontaneous nystagmus; SWJ=square-wave jerks; VGS=visually-guided saccades.

Abbreviations: AS=anti-saccades; aVOR=angular vestibulo-ocular reflex; FARS=Friedreich Ataxia Rating Scale; FRDA=Friedreich ataxia; GEN=gaze-evoked nystagmus; ICARS=International Cooperative Ataxia Rating Scale; MGS=memory-guided saccades; NA=not available; NPC=Niemann-Pick disease type C; OM=oculomotor; qHIT=quantitative head-impulse test; RBN=rebound nystagmus; SARA=Scale for the Assessment and Rating of Ataxia; SCA=spinocerebellar ataxia; *SCAFI=SCA Functional Index;* SEM=saccadic eye movements; SI=saccadic intrusions; SLCLC=Sloan Low-Contrast Letter Chart; SN=spontaneous nystagmus; PEM=pursuit eye movements; SCCs=semicircular canals; SWJ=square-wave jerks; VGS=visually-guided saccades.

| **Table A5-13: Scoring sheet – assessing quality of potential OM endpoint markers for hereditary ataxias**  **Disease rated: fragile X tremor ataxia syndrome (FTXAS)**  **Consensus ratings (PG and AAT)** | | | | | | |
| --- | --- | --- | --- | --- | --- | --- |
|  | **Oculomotor domain** | | | | | |
| **Parameter** | **Saccadic eye movements** | **Pursuit eye movements** | **Saccadic intrusions** | **Spontaneous nystagmus** | **Gaze-evoked nystagmus** | **Quantitative head-impulse test** |
| **Maximal number of studies reporting on a given parameter** | 4 (peak velocity and latency for VGS; latency for AS) | 1 (pursuit gain) | 1 (SWJ) | 0 | 0 | 0 |
| **Inclusion of carriers and/or early disease stages (0/1) *** | Yes | Yes (carriers only) | Yes (carriers only) | NaN | NaN | NaN |
| **Abnormalities detected (studies [n]) (0/1)** | - Normal to moderately increased AS latencies in carriers (n=1) - Moderately increased AS latencies in patients (n=2) - Increased AS error rate in patients and carriers (n=1) - Mildly increased latency in MGS (n=1) and overlap VGS (n=1) - Mild hypometria for 30° MGS (n=1) - Fewer and shorter fixations in patients (n=1) | None | NaN | NaN | NaN | NaN |
| **Discrimination between patients / controls (0/1)** | Assessed in 5 studies, significant differences in 5 | Assessed in 1 study, no significant differences | NaN | NaN | NaN | NaN |
| **Longitudinal data demonstrating significant changes §** | None | None | NaN | NaN | NaN | NaN |
| **Treatment response data demonstrating significant changes §** | None | None | NaN | NaN | NaN | NaN |
| **Significant correlation analyses #** | AS latency in patients with   - BDS-2 score - MMSE score   AS error rate in carriers with   - BDS-2 score - ICARS   VGS latency in patients with   - BDS-2 score   VGS metrics in carriers with   - ICARS   VGS latency and AS latency in carriers with   - ICARS   Inhibitory cost in patients (AS latency vs. VGS latency)   - CGG repeat length | None | NaN | NaN | NaN | NaN |
| **Preferred endpoint for given oculomotor domain** | **AS latency and error rate** |  | NaN | NaN | NaN | NaN |
|  |  |  |  |  |  |  |
| **Overall quality rating (points)** | **4** | **1** | NaN | NaN | NaN | NaN |

* Early disease stage defined as within 2 years of symptom onset (i.e., disease duration < 2 years).

§ Rating (points): 0=no data available or data available (from at least 1 study) demonstrated no significant changes; 1= data from at least 1 study available, demonstrating significant changes.

# Rating (points): 0=no correlation analyses available or correlation analyses available being non-significant or weak; 1=significant (moderate or strong) correlation analyses in one domain (domains=clinical scores, MRI, other imaging, genetics, clinical parameters [disease duration, age at symptom onset, age, time to manifestation], questionnaires); 2=significant (moderate or strong) correlation analyses in two or more domains.

**Abbreviations**: AS=anti-saccades; BDS-2= Behavioral Dyscontrol Scales 2; FXTAS=fragile-X tremor ataxia syndrome; MGS=memory-guided saccades; MMSE=mini mental state exam; MRI=magnetic resonance imaging; VGS=visually-guided saccades.

| **Table A5-14: Scoring sheet – assessing quality of potential OM endpoint markers for hereditary ataxias**  **Disease rated: Cerebrotendinous xanthomatosis (CTX)**  **Consensus ratings (PG and AAT)** | | | | | | |
| --- | --- | --- | --- | --- | --- | --- |
|  | **Oculomotor domain** | | | | | |
| **Parameter** | **Saccadic eye movements** | **Pursuit eye movements** | **Saccadic intrusions** | **Spontaneous nystagmus** | **Gaze-evoked nystagmus** | **Quantitative head-impulse test** |
| **Maximal number of studies reporting on a given parameter** | 1 (peak velocity, latency and metrics for VGS, latency and error rate for AS) | 0 | 1 (presence and frequency of saccadic intrusions) | 0 | 0 | 0 |
| **Inclusion of carriers and/or early disease stages (0/1) *** | No | NaN | No | NaN | NaN | NaN |
| **Abnormalities detected (studies [n]) (0/1)** | - Mildly increased latency for horizontal VGS in CTX patients with DN involvement (n=1) - Mildly dysmetric horizontal VGS in some CTX patients (n=2) - Moderately to strongly increased AS error rate (n=1) - Severely increased AS latency in CTX patients with DN involvement (n=1) | NaN | Microsaccadic oscillations in some patients (n=1) | NaN | NaN | NaN |
| **Discrimination between patients / controls (0/1)** | Assessed in 1 study, significant differences in 1 | NaN | None | NaN | NaN | NaN |
| **Longitudinal data demonstrating significant changes §** | None | NaN | None | NaN | NaN | NaN |
| **Treatment response data demonstrating significant changes §** | None | NaN | None | NaN | NaN | NaN |
| **Significant correlation analyses #** | None | NaN | None | NaN | NaN | NaN |
| **Preferred endpoint for given oculomotor domain** | **AS latency and error rate** | NaN | None | NaN | NaN | NaN |
|  |  | NaN |  | NaN | NaN | NaN |
| **Overall quality rating (points)** | **2** | NaN | **1** | NaN | NaN | NaN |

* Early disease stage defined as within 2 years of symptom onset (i.e., disease duration < 2 years).

§ Rating (points): 0=no data available or data available (from at least 1 study) demonstrated no significant changes; 1= data from at least 1 study available, demonstrating significant changes.

# Rating (points): 0=no correlation analyses available or correlation analyses available being non-significant or weak; 1=significant (moderate or strong) correlation analyses in one domain (domains=clinical scores, MRI, other imaging, genetics, clinical parameters [disease duration, age at symptom onset, age, time to manifestation], questionnaires); 2=significant (moderate or strong) correlation analyses in two or more domains.

**Abbreviations**: AS=anti-saccades; CTX=cerebrotendinous xanthomatosis; DN=dentate nucleus; MRI=magnetic resonance imaging; VGS=visually-guided saccades.

| **Table A5-15: Scoring sheet – assessing quality of potential OM endpoint markers for hereditary ataxias**  **Disease rated: SCA27B**  **Consensus ratings (PG and AAT)** | | | | | | |
| --- | --- | --- | --- | --- | --- | --- |
|  | **Oculomotor domain** | | | | | |
| **Parameter** | **Saccadic eye movements** | **Pursuit eye movements** | **Saccadic intrusions** | **Spontaneous nystagmus** | **Gaze-evoked nystagmus** | **Quantitative head-impulse test** |
| **Maximal number of studies reporting on a given parameter** | 0 | 0 | 0 | 2 (presence of SN) | 0 | 1 (vHIT gains) |
| **Inclusion of carriers and/or early disease stages (0/1) *** | NaN | NaN | NaN | No | NaN | No |
| **Abnormalities detected (studies [n]) (0/1)** | NaN | NaN | NaN | DBN in all patients (n=2) | NaN | Bilaterally reduced aVOR gains in some patients (n=1) |
| **Discrimination between patients / controls (0/1)** | NaN | NaN | NaN | yes | NaN | yes |
| **Longitudinal data demonstrating significant changes §** | NaN | NaN | NaN | No | NaN | No |
| **Treatment response data demonstrating significant changes §** | NaN | NaN | NaN | DBN SPV decreased after treatment with 4-AP | NaN | No |
| **Significant correlation analyses #** | NaN | NaN | NaN | No | NaN | No |
| **Preferred endpoint for given oculomotor domain** | NaN | NaN | NaN | Presence of DBN | NaN | vHIT gain |
|  |  |  |  |  |  |  |
| **Overall quality rating (points)** | NaN | NaN | NaN | 3 | NaN | 2 |

* Early disease stage defined as within 2 years of symptom onset (i.e., disease duration < 2 years).

§ Rating (points): 0=no data available or data available (from at least 1 study) demonstrated no significant changes; 1= data from at least 1 study available, demonstrating significant changes.

# Rating (points): 0=no correlation analyses available or correlation analyses available being non-significant or weak; 1=significant (moderate or strong) correlation analyses in one domain (domains=clinical scores, MRI, other imaging, genetics, clinical parameters [disease duration, age at symptom onset, age, time to manifestation], questionnaires); 2=significant (moderate or strong) correlation analyses in two or more domains.

**Summary of preferred paradigms for selected hereditary ataxias**

| **Table A5-16: Scoring sheet – assessing quality of potential OM endpoint markers for hereditary ataxias**  **Summary sheet**  **Consensus ratings (PG and AAT)** | | | | | | | |
| --- | --- | --- | --- | --- | --- | --- | --- |
|  | **Oculomotor domain** | | | | | | **Comments** |
| **Parameter** | **Saccadic eye movements** | **Pursuit eye movements** | **Saccadic intrusions** | **Spontaneous nystagmus** | **Gaze-evoked nystagmus** | **Quantitative head-impulse test** |  |
| **FRDA** | **VGS latency [1-7] and/or AS latency [3] (4 pts)** | Pursuit gain (2pts) | **Presence/frequency of SWJ [1, 2, 5, 8-14] (4 pts)** | Presence of DBN (2 pts) | Presence of GEN (1 pt) | Bilateral HC vHIT gains (2 pts) | Significant correlation analyses available for these parameters |
| **SCA1** | **Peak velocity VGS [1, 15-18] (2 pts)** | Pursuit gain (2 pts) | **Presence of SWJ [1, 17, 19] (2 pts)** | None | **Presence of GEN [17, 19] (2 pts)** | None | Most frequently observed abnormalities, but no correlation analyses available |
| **SCA2** | **Peak velocity VGS [15-17, 20-30] (7 pts)** | Pursuit gain (2 pt) | Presence of SWJ (2 pts) | None | Presence of GEN (2 pts) | None | Slow saccades are hallmark of SCA2, little data on other OM parameters |
| **SCA3** | **Peak velocity VGS [21, 31-35] (5 pts)** | Pursuit gain (5 pts) | Frequency of SWJ (4 pts) | SPV SN (3 pts) | **Presence of GEN [17, 19, 21, 32-34] (5 pts)** | **Bilateral HC vHIT gains [21, 32, 36-39] (6 pts)** | Several significant correlation analyses available, including pre-ataxic carriers |
| **SCA6** | **VGS metrics [17, 19, 21, 40, 41] (3 pts)** | **Pursuit gain [17, 18, 21, 40-43] (3 pts)** | Frequency of SWJ (3 pts) | **DBN [17, 19, 21, 40, 44] (3 pts)** | Presence of GEN (3 pts) | **Bilateral HC and VC vHIT gains [21, 45] (3 pts)** | Sign. correlations for vHIT gains and saccades. No sign. correlations for pre-ataxic carriers |
| **SCA7** | **Peak velocity VGS [21, 46] (2 pts)** | **Pursuit gain [21, 46] (2 pts)** | None | None | None | **Bilateral HC and VC vHIT gains [21, 46] (2 pts)** | Limited data, only two publications (with a total of 11 patients) identified and only three OM domains studied. No correlation analyses. |
| **EA2** | **Pursuit gain [23, 54, 57-59] (2 pts)** | **Bilateral HC and VC vHIT gains [30, 42, 47, 49, 50] (2 pts)** | **Presence of GEN [49, 51] (1 pt)** | None | None | None | No correlation analyses. No longitudinal data and no treatment trials. |
| **A-T** | **VGS hypometria [47-49] (2 pts)** | **Pursuit gain [47-50] (4 pts)** | **Presence of SI [48, 49, 51] (2 pts)** | **Presence of DBN [49, 51, 52] (3 pts)** | Presence of GEN (2 pts) | Bilteral vHIT gains (1 pt) | Most data on VGS metrics, significant correlations for PEM and significant treatment response for DBN. Frequent SWJ |
| **NPC** | **Peak velocity vertical VGS [69-71, 74, 80, 81]**  **(5 pts)** | Pursuit gain (1 pt) | NA | NA | None (0 pts) | None (0 pts) | Most data on vertical SEM, clinical key finding. Significant correlations for SEM and significant treatment response to miglustat |
| **RFC1-related disease** | NA | NA | NA | **Presence of DBN [56] (1 pt)** | NA | **Bilateral HC vHIT gain [56-59] (3 pts)** | Limited data, only two domains studied. Combination of peripheral and central vestibular deficits is hallmark sign. |
| **AOA1** | **Metrics of VGS [49, 60] and AS error rate [49, 60] (2 pts)** | None (0 pts) | Presence and frequency of SWJ (1 pt) | Presence of horizontal SN (1 pt) | **Presence of horizontal GEN [49] (2 pts)** | NA | Limited data, only two publications identified. No correlation analyses available. No qHIT data. |
| **AOA2** | **VGS (metrics [49, 61-63] / velocity [49, 61, 62]) and AS error rate [49, 61-63] (2 pts)** | None (0 pts) | Presence and frequency of SWJ (2 pts) | Presence of horizontal SN (2 pts) | **Presence of horizontal GEN [49] (2 pts)** | NA | Very limited data on most OM domains (PEM, SI, SN, GEN). No correlation analyses. No qHIT data. |
| **FXTAS** | **AS latency [64-67] and error rate [64-67] (4 pts)** | Pursuit gain (1 pts) | NA | NA | NA | NA | Most data on SEM, very limited (PEM) or lacking data for other OM domains (SI, SN, GEN, qHIT). |
| **CTX** | **VGS (metrics [68, 69] / latency [68]) and AS latency [68] and error rate [68] (2 pts)** | NA | Presence and frequency of SI (1 pt) | NA | NA | NA | Limited data, only two publications (with a total of 23 patients) identified and only two OM domains studied. No correlation analyses. |
| **SCA27B** | NA | NA | NA | **Presence of DBN [70, 71] (3 pts)** | NA | **Bilateral HC vHIT gain [70] (2 points)** | Limited data, only two domains studied. Mild aVOR impairment only. |

**Abbreviations**: AOA=ataxia with oculomotor apraxia; AS=anti-saccades; A-T=ataxia telangiectasia; aVOR=angular vestibulo-ocular reflex; CTX=cerebrotendinous xanthomatosis; DBN=downbeat nystagmus; FRDA=Friedreich Ataxia; fXPCs=fragile X premutation carriers; FXTAS=fragile-X associated tremor/ataxia syndrome; GEN=gaze-evoked nystagmus; HC=horizontal canal; Hor=horizontal; MGS=memory-guided saccades; MRI=magnetic resonance imaging; NPC=Niemann-Pick disease Type C; OM=oculomotor; PEM=pursuit eye movements; PV=peak velocity; qHIT=quantitative head-impulse test; RFC1=replication factor C subunit 1; SCA=spinocerebellar ataxia; SEM=saccadic eye movements; SI=saccadic intrusions; SN=spontaneous nystagmus; SPV=slow phase velocity; SWJ=square-wave jerks; Tc=time constant; tx=treatment; VC=vertical canal; VGS=visually-guided saccades; vHIT=video-head-impulse test.

**References**

[1] Alexandre MF, Rivaud-Péchoux S, Challe G, Durr A and Gaymard B. Functional consequences of oculomotor disorders in hereditary cerebellar ataxias. Cerebellum (London, England) 2013: 12:396-405. doi 10.1007/s12311-012-0433-z

[2] Fahey MC, Cremer PD, Aw ST, Millist L, Todd MJ, White OB, Halmagyi M, Corben LA, Collins V, Churchyard AJ, Tan K, Kowal L and Delatycki MB. Vestibular, saccadic and fixation abnormalities in genetically confirmed Friedreich ataxia. Brain : a journal of neurology 2008: 131:1035-45. doi 10.1093/brain/awm323

[3] Fielding J, Corben L, Cremer P, Millist L, White O and Delatycki M. Disruption to higher order processes in Friedreich ataxia. Neuropsychologia 2010: 48:235-42. doi 10.1016/j.neuropsychologia.2009.09.009

[4] Hocking DR, Corben LA, Fielding J, Cremer PD, Millist L, White OB and Delatycki MB. Saccade reprogramming in Friedreich ataxia reveals impairments in the cognitive control of saccadic eye movement. Brain and cognition 2014: 87:161-7. doi 10.1016/j.bandc.2014.03.018

[5] Wessel K, Moschner C, Wandinger KP, Kömpf D and Heide W. Oculomotor testing in the differential diagnosis of degenerative ataxic disorders. Archives of neurology 1998: 55:949-56. doi 10.1001/archneur.55.7.949

[6] Moschner C, Perlman S and Baloh RW. Comparison of oculomotor findings in the progressive ataxia syndromes. Brain : a journal of neurology 1994: 117 ( Pt 1):15-25. doi 10.1093/brain/117.1.15

[7] Hocking DR, Fielding J, Corben LA, Cremer PD, Millist L, White OB and Delatycki MB. Ocular motor fixation deficits in Friedreich ataxia. Cerebellum (London, England) 2010: 9:411-8. doi 10.1007/s12311-010-0178-5

[8] Baloh RW, Konrad HR and Honrubia V. Vestibulo-ocular function in patients with cerebellar atrophy. Neurology 1975: 25:160-8. doi 10.1212/wnl.25.2.160

[9] Ciuffreda KJ, Kenyon RV and Stark L. Eye movements during reading: further case reports. American journal of optometry and physiological optics 1985: 62:844-52. doi 10.1097/00006324-198512000-00005

[10] Dale RT, Kirby AW and Jampel RS. Square wave jerks in Friedreich's ataxia. American journal of ophthalmology 1978: 85:400-6. doi 10.1016/s0002-9394(14)77738-4

[11] Ell J, Prasher D and Rudge P. Neuro-otological abnormalities in Friedreich's ataxia. Journal of neurology, neurosurgery, and psychiatry 1984: 47:26-32. doi 10.1136/jnnp.47.1.26

[12] Furman JM, Perlman S and Baloh RW. Eye movements in Friedreich's ataxia. Archives of neurology 1983: 40:343-6. doi 10.1001/archneur.1983.04050060043006

[13] Ribaï P, Pousset F, Tanguy ML, Rivaud-Pechoux S, Le Ber I, Gasparini F, Charles P, Béraud AS, Schmitt M, Koenig M, Mallet A, Brice A and Dürr A. Neurological, cardiological, and oculomotor progression in 104 patients with Friedreich ataxia during long-term follow-up. Archives of neurology 2007: 64:558-64. doi 10.1001/archneur.64.4.558

[14] Spieker S, Schulz JB, Petersen D, Fetter M, Klockgether T and Dichgans J. Fixation instability and oculomotor abnormalities in Friedreich's ataxia. Journal of neurology 1995: 242:517-21. doi 10.1007/bf00867423

[15] Bürk K, Fetter M, Skalej M, Laccone F, Stevanin G, Dichgans J and Klockgether T. Saccade velocity in idiopathic and autosomal dominant cerebellar ataxia. Journal of neurology, neurosurgery, and psychiatry 1997: 62:662-4. doi 10.1136/jnnp.62.6.662

[16] Bürk K, Abele M, Fetter M, Dichgans J, Skalej M, Laccone F, Didierjean O, Brice A and Klockgether T. Autosomal dominant cerebellar ataxia type I clinical features and MRI in families with SCA1, SCA2 and SCA3. Brain : a journal of neurology 1996: 119 ( Pt 5):1497-505. doi 10.1093/brain/119.5.1497

[17] Buttner N, Geschwind D, Jen JC, Perlman S, Pulst SM and Baloh RW. Oculomotor phenotypes in autosomal dominant ataxias. Archives of neurology 1998: 55:1353-7. doi 10.1001/archneur.55.10.1353

[18] Kerber KA, Jen JC, Perlman S and Baloh RW. Late-onset pure cerebellar ataxia: differentiating those with and without identifiable mutations. Journal of the neurological sciences 2005: 238:41-5. doi 10.1016/j.jns.2005.06.006

[19] Kim JS, Kim JS, Youn J, Seo DW, Jeong Y, Kang JH, Park JH and Cho JW. Ocular motor characteristics of different subtypes of spinocerebellar ataxia: distinguishing features. Movement disorders : official journal of the Movement Disorder Society 2013: 28:1271-7. doi 10.1002/mds.25464

[20] Anderson JH, Christova PS, Xie TD, Schott KS, Ward K and Gomez CM. Spinocerebellar ataxia in monozygotic twins. Archives of neurology 2002: 59:1945-51. doi 10.1001/archneur.59.12.1945

[21] Kim JM, Nam TS, Choi SM, Kim BC and Lee SH. Clinical value of vestibulo-ocular reflex in the differentiation of spinocerebellar ataxias. Scientific reports 2023: 13:14783. doi 10.1038/s41598-023-41924-6

[22] Federighi P, Cevenini G, Dotti MT, Rosini F, Pretegiani E, Federico A and Rufa A. Differences in saccade dynamics between spinocerebellar ataxia 2 and late-onset cerebellar ataxias. Brain : a journal of neurology 2011: 134:879-91. doi 10.1093/brain/awr009

[23] Reetz K, Rodríguez-Labrada R, Dogan I, Mirzazade S, Romanzetti S, Schulz JB, Cruz-Rivas EM, Alvarez-Cuesta JA, Aguilera Rodríguez R, Gonzalez Zaldivar Y, Auburger G and Velázquez-Pérez L. Brain atrophy measures in preclinical and manifest spinocerebellar ataxia type 2. Annals of clinical and translational neurology 2018: 5:128-37. doi 10.1002/acn3.504

[24] Rodríguez-Labrada R, Vázquez-Mojena Y, Canales-Ochoa N, Medrano-Montero J and Velázquez-Pérez L. Heritability of saccadic eye movements in spinocerebellar ataxia type 2: insights into an endophenotype marker. Cerebellum & ataxias 2017: 4:19. doi 10.1186/s40673-017-0078-2

[25] Rodríguez-Labrada R, Velázquez-Pérez L, Auburger G, Ziemann U, Canales-Ochoa N, Medrano-Montero J, Vázquez-Mojena Y and González-Zaldivar Y. Spinocerebellar ataxia type 2: Measures of saccade changes improve power for clinical trials. Movement disorders : official journal of the Movement Disorder Society 2016: 31:570-8. doi 10.1002/mds.26532

[26] Rufa A and Federighi P. Fast versus slow: different saccadic behavior in cerebellar ataxias. Annals of the New York Academy of Sciences 2011: 1233:148-54. doi 10.1111/j.1749-6632.2011.06126.x

[27] Seifried C, Velázquez-Pérez L, Santos-Falcón N, Abele M, Ziemann U, Almaguer LE, Martínez-Góngora E, Sánchez-Cruz G, Canales N, Pérez-González R, Velázquez-Manresa M, Viebahn B, Stuckrad-Barre S, Klockgether T, Fetter M and Auburger G. Saccade velocity as a surrogate disease marker in spinocerebellar ataxia type 2. Annals of the New York Academy of Sciences 2005: 1039:524-7. doi 10.1196/annals.1325.059

[28] Velázquez-Pérez L, Rodríguez-Labrada R, Álvarez-González L, Aguilera-Rodríguez R, Álvarez Sánchez M, Canales-Ochoa N, Galicia Polo L, Haro-Valencia R, Medrano-Montero J, Vázquez-Mojena Y, Peña-Acosta A, Estupiñán-Rodríguez A and Rodríguez Pupo N. Lisuride reduces involuntary periodic leg movements in spinocerebellar ataxia type 2 patients. Cerebellum (London, England) 2012: 11:1051-6. doi 10.1007/s12311-012-0382-6

[29] Velázquez-Pérez L, Rodríguez-Chanfrau J, García-Rodríguez JC, Sánchez-Cruz G, Aguilera-Rodríguez R, Rodríguez-Labrada R, Rodríguez-Díaz JC, Canales-Ochoa N, Gotay DA, Almaguer Mederos LE, Laffita Mesa JM, Porto-Verdecia M, Triana CG, Pupo NR, Batista IH, López-Hernandez OD, Polanco ID and Novas AJ. Oral zinc sulphate supplementation for six months in SCA2 patients: a randomized, double-blind, placebo-controlled trial. Neurochemical research 2011: 36:1793-800. doi 10.1007/s11064-011-0496-0

[30] Rodriguez-Labrada R, Ortega-Sanchez R, Hernandez Casana P, Santos Morales O, Padron-Estupinan MDC, Batista-Nunez M, Jimenez Rodriguez D, Canales-Ochoa N, Pena Acosta A, Medrano Montero J, Labrada Aguilera PE, Estupinan Rodriguez A, Vazquez-Mojena Y, Almaguer Gotay D, Aymed-Garcia J, Garcia-Garcia I, Torres Vega R, Viada Gonzalez C, Valenzuela Silva CM, Silva Ricardo Y, Columbie Ximelis J, Tribin Rivero K, Valle Cabrera R, Garcia-Rodriguez JC, Crombet Ramos T, Amaro-Gonzalez D, Rodriguez-Obaya T and Velazquez-Perez L. Erythropoietin in Spinocerebellar Ataxia Type 2: Feasibility and Proof-of-Principle Issues from a Randomized Controlled Study. Movement disorders : official journal of the Movement Disorder Society 2022: 37:1516-25. doi 10.1002/mds.29045

[31] Caspi A, Zivotofsky AZ and Gordon CR. Multiple saccadic abnormalities in spinocerebellar ataxia type 3 can be linked to a single deficiency in velocity feedback. Investigative ophthalmology & visual science 2013: 54:731-8. doi 10.1167/iovs.12-10689

[32] de Oliveira CM, Leotti VB, Bolzan G, Cappelli AH, Rocha AG, Ecco G, Kersting N, Rieck M, Martins AC, Sena LS, Saraiva-Pereira ML and Jardim LB. Pre-ataxic Changes of Clinical Scales and Eye Movement in Machado-Joseph Disease: BIGPRO Study. Movement disorders : official journal of the Movement Disorder Society 2021. doi 10.1002/mds.28466

[33] Ghasia FF, Wilmot G, Ahmed A and Shaikh AG. Strabismus and Micro-Opsoclonus in Machado-Joseph Disease. Cerebellum (London, England) 2016: 15:491-7. doi 10.1007/s12311-015-0718-0

[34] Lemos J, Novo A, Duque C, Castelhano J, Eggenberger E and Januário C. "Pinball" intrusions in spinocerebellar ataxia type 3. Neurology 2018: 90:36-7. doi 10.1212/wnl.0000000000004772

[35] Wu C, Chen DB, Feng L, Zhou XX, Zhang JW, You HJ, Liang XL, Pei Z and Li XH. Oculomotor deficits in spinocerebellar ataxia type 3: Potential biomarkers of preclinical detection and disease progression. CNS Neurosci Ther 2017: 23:321-8. doi 10.1111/cns.12676

[36] Elyoseph Z, Geisinger D, Zaltzman R, Mintz M and Gordon CR. Horizontal Vestibulo-Ocular Reflex Deficit as a Biomarker for Clinical Disease Onset, Severity, and Progression of Machado-Joseph Disease. Cerebellum (London, England) 2023. doi 10.1007/s12311-023-01552-2

[37] Luis L, Costa J, Munoz E, de Carvalho M, Carmona S, Schneider E, Gordon CR and Valls-Sole J. Vestibulo-ocular reflex dynamics with head-impulses discriminates spinocerebellar ataxias types 1, 2 and 3 and Friedreich ataxia. Journal of vestibular research : equilibrium & orientation 2016: 26:327-34. doi 10.3233/VES-160579

[38] Geisinger D, Elyoseph Z, Zaltzman R, Mintz M and Gordon CR. Angular vestibulo ocular reflex loss with preserved saccular function in Machado-Joseph disease. Journal of the neurological sciences 2021: 424:117393. doi 10.1016/j.jns.2021.117393

[39] Gordon CR, Zivotofsky AZ and Caspi A. Impaired vestibulo-ocular reflex (VOR) in spinocerebellar ataxia type 3 (SCA3): bedside and search coil evaluation. Journal of vestibular research : equilibrium & orientation 2014: 24:351-5. doi 10.3233/ves-140527

[40] Bour LJ, van Rootselaar AF, Koelman JH and Tijssen MA. Oculomotor abnormalities in myoclonic tremor: a comparison with spinocerebellar ataxia type 6. Brain : a journal of neurology 2008: 131:2295-303. doi 10.1093/brain/awn177

[41] Christova P, Anderson JH and Gomez CM. Impaired eye movements in presymptomatic spinocerebellar ataxia type 6. Archives of neurology 2008: 65:530-6. doi 10.1001/archneur.65.4.530

[42] Takeichi N, Fukushima K, Sasaki H, Yabe I, Tashiro K and Inuyama Y. Dissociation of smooth pursuit and vestibulo-ocular reflex cancellation in SCA-6. Neurology 2000: 54:860-6. doi 10.1212/wnl.54.4.860

[43] Wiest G, Tian JR, Baloh RW, Crane BT and Demer JL. Otolith function in cerebellar ataxia due to mutations in the calcium channel gene CACNA1A. Brain : a journal of neurology 2001: 124:2407-16. doi 10.1093/brain/124.12.2407

[44] Gomez CM, Thompson RM, Gammack JT, Perlman SL, Dobyns WB, Truwit CL, Zee DS, Clark HB and Anderson JH. Spinocerebellar ataxia type 6: gaze-evoked and vertical nystagmus, Purkinje cell degeneration, and variable age of onset. Annals of neurology 1997: 42:933-50. doi 10.1002/ana.410420616

[45] Huh YE, Kim JS, Kim HJ, Park SH, Jeon BS, Kim JM, Cho JW and Zee DS. Vestibular Performance During High-Acceleration Stimuli Correlates with Clinical Decline in SCA6. Cerebellum (London, England) 2015: 14:284-91. doi 10.1007/s12311-015-0650-3

[46] Oh AK, Jacobson KM, Jen JC and Baloh RW. Slowing of voluntary and involuntary saccades: an early sign in spinocerebellar ataxia type 7. Annals of neurology 2001: 49:801-4. doi 10.1002/ana.1059

[47] Lewis RF and Crawford TO. Slow target-directed eye movements in ataxia-telangiectasia. Investigative ophthalmology & visual science 2002: 43:686-91.

[48] Lewis RF, Lederman HM and Crawford TO. Ocular motor abnormalities in ataxia telangiectasia. Annals of neurology 1999: 46:287-95. doi 10.1002/1531-8249(199909)46:3<287::aid-ana3>3.0.co;2-0

[49] Mariani LL, Rivaud-Pechoux S, Charles P, Ewenczyk C, Meneret A, Monga BB, Fleury MC, Hainque E, Maisonobe T, Degos B, Echaniz-Laguna A, Renaud M, Wirth T, Grabli D, Brice A, Vidailhet M, Stoppa-Lyonnet D, Dubois-d'Enghien C, Le Ber I, Koenig M, Roze E, Tranchant C, Durr A, Gaymard B and Anheim M. Comparing ataxias with oculomotor apraxia: a multimodal study of AOA1, AOA2 and AT focusing on video-oculography and alpha-fetoprotein. Scientific reports 2017: 7:15284. doi 10.1038/s41598-017-15127-9

[50] Baloh RW, Yee RD and Boder E. Eye movements in ataxia-telangiectasia. Neurology 1978: 28:1099-104. doi 10.1212/wnl.28.11.1099

[51] Shaikh AG, Marti S, Tarnutzer AA, Palla A, Crawford TO, Straumann D, Taylor AM and Zee DS. Gaze fixation deficits and their implication in ataxia-telangiectasia. Journal of neurology, neurosurgery, and psychiatry 2009: 80:858-64. doi 10.1136/jnnp.2008.170522

[52] Brueggemann A, Bicvic A, Goeldlin M, Kalla R, Kerkeni H, Mantokoudis G, Abegg M, Kolnikova M, Mohaupt M and Bremova-Ertl T. Effects of Acetyl-DL-Leucine on Ataxia and Downbeat-Nystagmus in Six Patients With Ataxia Telangiectasia. J Child Neurol 2022: 37:20-7. doi 10.1177/08830738211028394

[53] Solomon D, Winkelman AC, Zee DS, Gray L and Büttner-Ennever J. Niemann-Pick type C disease in two affected sisters: ocular motor recordings and brain-stem neuropathology. Annals of the New York Academy of Sciences 2005: 1039:436-45. doi 10.1196/annals.1325.041

[54] Havla J, Moser M, Sztatecsny C, Lotz-Havla AS, Maier EM, Hizli B, Schinner R, Kümpfel T, Strupp M, Bremova-Ertl T and Schneider SA. Retinal axonal degeneration in Niemann-Pick type C disease. Journal of neurology 2020: 267:2070-82. doi 10.1007/s00415-020-09796-2

[55] Bremova T, Malinova V, Amraoui Y, Mengel E, Reinke J, Kolnikova M and Strupp M. Acetyl-dl-leucine in Niemann-Pick type C: A case series. Neurology 2015: 85:1368-75. doi 10.1212/WNL.0000000000002041

[56] Costales M, Casanueva R, Suárez V, Asensi JM, Cifuentes GA, Diñeiro M, Cadiñanos J, López F, Álvarez-Marcos C, Otero A, Gómez J, Llorente JL and Cabanillas R. CANVAS: A New Genetic Entity in the Otorhinolaryngologist's Differential Diagnosis. Otolaryngology--head and neck surgery : official journal of American Academy of Otolaryngology-Head and Neck Surgery 2021:1945998211008398. doi 10.1177/01945998211008398

[57] Borsche M, Tadic V, Konig IR, Lohmann K, Helmchen C and Bruggemann N. Head impulse testing in bilateral vestibulopathy in patients with genetically defined CANVAS. Brain Behav 2022: 12:e32546. doi 10.1002/brb3.2546

[58] Pellerin D, Heindl F, Traschutz A, Rujescu D, Hartmann AM, Brais B, Houlden H, Dufke C, Riess O, Haack T, Strupp M and Synofzik M. RFC1 repeat expansions in downbeat nystagmus syndromes: frequency and phenotypic profile. Journal of neurology 2024: 271:2886-92. doi 10.1007/s00415-024-12229-z

[59] Harrell RG, Cassidy AR, Klatt BN, Hovareshti P and Whitney SL. Vestibular rehabilitation in cerebellar ataxia with neuropathy and vestibular areflexia syndrome (CANVAS)- A case report. J Otol 2023: 18:199-207. doi 10.1016/j.joto.2023.06.004

[60] Le Ber I, Moreira MC, Rivaud-Pechoux S, Chamayou C, Ochsner F, Kuntzer T, Tardieu M, Said G, Habert MO, Demarquay G, Tannier C, Beis JM, Brice A, Koenig M and Durr A. Cerebellar ataxia with oculomotor apraxia type 1: clinical and genetic studies. Brain : a journal of neurology 2003: 126:2761-72. doi 10.1093/brain/awg283

[61] Bargagli A, Rosini F, Zanca D, Serchi V and Rufa A. Ataxia with oculomotor apraxia type 2 (AOA2): an eye movement study of two siblings. Neurological sciences : official journal of the Italian Neurological Society and of the Italian Society of Clinical Neurophysiology 2021: 42:3039-42. doi 10.1007/s10072-021-05206-1

[62] Le Ber I, Bouslam N, Rivaud-Pechoux S, Guimaraes J, Benomar A, Chamayou C, Goizet C, Moreira MC, Klur S, Yahyaoui M, Agid Y, Koenig M, Stevanin G, Brice A and Durr A. Frequency and phenotypic spectrum of ataxia with oculomotor apraxia 2: a clinical and genetic study in 18 patients. Brain : a journal of neurology 2004: 127:759-67. doi 10.1093/brain/awh080

[63] Panouilleres M, Frismand S, Sillan O, Urquizar C, Vighetto A, Pelisson D and Tilikete C. Saccades and eye-head coordination in ataxia with oculomotor apraxia type 2. Cerebellum (London, England) 2013: 12:557-67. doi 10.1007/s12311-013-0463-1

[64] McLennan YA, Mosconi MW, McKenzie FJ, Famula J, Krawchuk B, Kim K, Clark CJ, Hessl D, Rivera SM, Simon TJ, Tassone F and Hagerman RJ. Prosaccade and Antisaccade Behavior in Fragile X-Associated Tremor/Ataxia Syndrome Progression. Mov Disord Clin Pract 2022: 9:473-8. doi 10.1002/mdc3.13449

[65] Fielding-Gebhardt H, Kelly SE, Unruh KE, Schmitt LM, Pulver SL, Khemani P and Mosconi MW. Sensorimotor and inhibitory control in aging FMR1 premutation carriers. Front Hum Neurosci 2023: 17:1271158. doi 10.3389/fnhum.2023.1271158

[66] Wong LM, Goodrich-Hunsaker NJ, McLennan Y, Tassone F, Zhang M, Rivera SM and Simon TJ. Eye movements reveal impaired inhibitory control in adult male fragile X premutation carriers asymptomatic for FXTAS. Neuropsychology 2014: 28:571-84. doi 10.1037/neu0000066

[67] Lasker AG, Mazzocco MM and Zee DS. Ocular motor indicators of executive dysfunction in fragile X and Turner syndromes. Brain and cognition 2007: 63:203-20. doi 10.1016/j.bandc.2006.08.002

[68] Rosini F, Pretegiani E, Mignarri A, Optican LM, Serchi V, De Stefano N, Battaglini M, Monti L, Dotti MT, Federico A and Rufa A. The role of dentate nuclei in human oculomotor control: insights from cerebrotendinous xanthomatosis. J Physiol 2017: 595:3607-20. doi 10.1113/JP273670

[69] Koens LH, Tuitert I, Blokzijl H, Engelen M, Klouwer FCC, Lange F, Leen WG, Lunsing RJ, Koelman J, Verrips A, de Koning TJ and Tijssen MAJ. Eye movement disorders in inborn errors of metabolism: A quantitative analysis of 37 patients. J Inherit Metab Dis 2022: 45:981-95. doi 10.1002/jimd.12533

[70] Pellerin D, Heindl F, Wilke C, Danzi MC, Traschutz A, Ashton C, Dicaire MJ, Cuillerier A, Del Gobbo G, Boycott KM, Claassen J, Rujescu D, Hartmann AM, Zuchner S, Brais B, Strupp M and Synofzik M. GAA-FGF14 disease: defining its frequency, molecular basis, and 4-aminopyridine response in a large downbeat nystagmus cohort. EBioMedicine 2024: 102:105076. doi 10.1016/j.ebiom.2024.105076

[71] Shirai S, Mizushima K, Fujiwara K, Koshimizu E, Matsushima M, Miyatake S, Iwata I, Yaguchi H, Matsumoto N and Yabe I. Case series: Downbeat nystagmus in SCA27B. Journal of the neurological sciences 2023: 454:120849. doi 10.1016/j.jns.2023.120849
