# Supplementary material for: Best Oculomotor Endpoints for Clinical Trials in Hereditary Ataxias: A Systematic Review and Consensus by the Ataxia Global Initiative Working Group on Digital‑Motor Biomarkers
Source: Cerebellum. 2025 Aug 13;24(5):141. doi: 10.1007/s12311-025-01894-z (PMC12350468; doi:10.1007/s12311-025-01894-z)
Supplement: Supplementary file 4 — Supplementary file4 (DOCX 447 KB) [file 12311_2025_1894_MOESM4_ESM.docx]

**Appendix 6 – Proposed disease-specific parameters for selected OM / vestibular paradigms**

**Table A6-1: Disease-specific parameters for selected OM / vestibular paradigms in order of priority (1-4)***

| **Disease** | **OM-domain 1 - selected parameter(s)** | **OM-domain 2 - selected parameter(s)** | **OM-domain 3 - selected parameter(s)** | **OM-domain 4 - selected parameter(s)** | **Comments** |
| --- | --- | --- | --- | --- | --- |
| **FRDA** | **VGS latency [1-7]:**   - Horizontal saccades (random order, centripetal / centrifugal) - Amplitudes: ±10°, ±20° and ±40° - Inter-step interval: 0.5-2.5sec - >10 trials per condition - **Endpoint: significant changes in VGS latency**   **AS latency [3]:**   - Duration of center target: 150msec - Horizontal target (±5°, ±10°) - Instruction to look in opposite hemifield as quickly as possible - >10 trials per condition - **Endpoint: significant changes in AS latency** | **Presence/frequency of SWJ [1, 2, 5, 8-14]**   - 2 conditions: central target-on and central target-off (with refixation flash for 50msec every 2sec) - Recording duration: 60sec each. - Distinction between micro-SWJ, SWJ and macro-SWJ. - Analysis of frequency and amplitude of SWJ. - **Endpoint: significant changes in frequency and/or amplitude of SWJ** | None | None | - No longitudinal data - 1 treatment trial with non-significant findings |
| **SCA1** | **PV VGS [1, 15-18]**   - Horizontal saccades (random order, centripetal / centrifugal) - Amplitudes: ±5°, ±10°, ±20° and ±30° - Inter-step interval: 0.5-2.5sec - >10 trials per condition - **Endpoint: significant changes in VGS peak velocity** | **Presence of SWJ [1, 17, 19]**   - Same parameters as for FRDA - **Endpoint: significant changes in frequency and/or amplitude of SWJ** | **Presence of GEN [17, 19]**   - Horizontal eccentric gaze-holding at ±30° eccentricity over 10sec (in darkness, with refixation flash for 50msec every 2sec). - Analysis of horizontal SPV at eccentric gaze - **Endpoint: significant changes in SPV GEN** | None | - No longitudinal data - No treatment trials - No significant correlations with other parameters |
| **SCA2** | **PV VGS [15-17, 20-30]**   - Horizontal saccades (random order, centripetal / centrifugal) - Amplitudes: ±5°, ±10°, ±30°, and ±60° - Inter-step interval: 0.5-2.5sec - >10 trials per condition - **Endpoint: significant changes in VGS peak velocity** | None | None | None | - Pvel and accuracy sign. decreased and latency sign. increased over 60 months [24], no sign. changes over 12 months [26] - Saccade latencies reduced after treatment with zinc sulfate [28] - Saccade latency sign. decreased in NeuroEPO group, but not in placebo group [29] |
| **SCA3** | **PV VGS [30-35]**   - Vertical saccades (random order, centripetal / centrifugal) - Amplitudes: ±10°, ±15°, and ±20° - Inter-step interval: 1-2sec - **Endpoint: significant changes in vertical VGS peak velocity** | **Presence of GEN [17, 19, 30, 32-34]**   - Same parameters as for SCA1 - **Endpoint: significant changes in SPV GEN** | **HC and VC vHIT gains [30, 32, 36-39]**   - Impulse-amplitude: 10-20° - Impulse-velocity: 150-300°/sec - At least 10 trials per canal - aVOR gain calculation depends on the VOG device used - Cut-off values as published by manufacturer (<0.8 for HC) or as found in healthy controls. - **Endpoint: significant changes in HC vHIT gains** | none | - Change in SARA over time correlated with change in HC vHIT gain over time [30, 36] |
| **SCA6** | **VGS dysmetria [17, 19, 40-42]**   - Horizontal saccades (random order, centripetal / centrifugal) - Amplitudes: ±5°, ±10°, ±20° and ±39° - Inter-step interval: 0.5-3sec - >10 trials per condition - **Endpoint: significant changes in VGS metrics** | **Pursuit gain [17, 18, 30, 40-45]**   - Horizontal pursuit eye movements - Sinusoidally moving target at frequencies of 0.1, 0.2, and 0.4 Hz (PV: 11.3/s, 22.6/s, 45.2/s). - **Endpoint: significant changes in PEM gain** | **Presence of DBN [17, 19, 30, 42, 43, 46]**   - Recording of SN both with central target-on and target-off (with refixation flash for 50msec every 2sec) - Duration: 60sec per condition - **Endpoint: significant changes in SPV DBN** | **HC and VC vHIT gains [30, 42, 47]**   - Same parameters as for SCA3 - **Endpoint: significant changes in HC and/or VC vHIT gains** | - SARA correlated with HC, AC and PC vHIT gains [42] - At follow-up (after 12 months [median], IQR=9-50 months) HC and AC vHIT gains significantly decreased, but PC vHIT gains remained unchanged [42] - No changes in OM parameters over time in pre-symptomatic carriers [40] - No treatment trials |
| **SCA7** | **PV VGS [30, 48]**   - Horizontal saccades (random order, centripetal / centrifugal) - Amplitudes: ±5°, ±10°, and ±20° - Inter-step interval: 0.5-2.5sec - >10 trials per condition - **Endpoint: significant changes in VGS peak velocity** | **Pursuit gain [30, 48]**   - Same parameters as for SCA6 - **Endpoint: significant changes in PEM gain** | **HC and VC vHIT gains [30, 48]**   - Same parameters as for SCA3 - **Endpoint: significant changes in HC and/or VC vHIT gains** |  | - No longitudinal data - No treatment trials - Only three OM domains studied |
| **EA2** | **Pursuit gain [20, 45, 49-51]**   - Same parameters as for SCA6 - **Endpoint: significant changes in PEM gain** | **HC and VC vHIT gains [30, 42, 47, 49, 50]**   - Same parameters as for SCA3 - **Endpoint: significant changes in HC and/or VC vHIT gains** | **Presence of GEN [49, 51]**   - Same parameters as for SCA1 - **Endpoint: significant changes in SPV GEN** |  | - No longitudinal data - No treatment trials - No significant correlations with other parameters |
| **A-T** | **VGS hypometria [52-54]**   - Horizontal saccades (random order, from center to target) - Amplitudes: ±10° and ±20° - Center target on: 1-4sec - Inter-step interval: 2-3sec - >10 trials per condition - **Endpoint: significant changes in VGS metrics** | **Pursuit gain [52-55]**   - Same parameters as for SCA6 - **Endpoint: significant changes in PEM gain** | **Presence of DBN [54, 56-58]**   - Same parameters as for SCA6 - **Endpoint: significant changes in SPV DBN** | **Presence of SWJ**  **[53, 54, 56]**   - Same parameters as for FRDA - **Endpoint: significant changes in frequency and/or amplitude of SWJ** | - SPV DBN sign. decreased under acetyl-DL-leucine tx vs. placebo (after 1 month) [58] - VOR Tc and SN SPV decreased after treatment with 4-AP [57] |
| **NPC** | **PV VGS [59-64]**   - Vertical saccades (random order, from center to target) - Amplitudes: ±10° and ±20° - Center target on: 1-4sec - Inter-step interval: 2-3sec - **Endpoint: significant changes in vertical VGS peak velocity** | None | None | None | - No changes in OM parameters under treatment with acetyl-DL-leucine after 4 weeks [61] - Improvement of horizontal VGS PV and gain under miglustat [65, 66], with evaluation after 12 months [67-69]and 24 months of treatment [68, 70] |
| **RFC1-related ataxia** | **HC and VC vHIT gains [71-74]**   - Same parameters as for SCA3 - **Endpoint: significant changes in HC vHIT gains** | **Presence of DBN [71]**   - Same parameters as for SCA6 - **Endpoint: significant changes in SPV DBN** | None | none | - No longitudinal data - No treatment trials - Only two OM domains studied |
| **SCA27B** | **Presence of DBN [75, 76]**   - Same parameters as for SCA6 - **Endpoint: significant changes in SPV DBN** | **Bilateral HC vHIT gain [75]**   - Same parameters as for SCA3 - **Endpoint: significant changes in HC vHIT gains** |  |  | - Limited data, only two domains studied. - Mild aVOR impairment only. |
| **AOA1** | **VGS dysmetria [54, 77]**   - Horizontal saccades (random order, from center to target) - Amplitudes: ±13° and ±25° - Duration of center target: 1-4sec - Inter-step interval: 2-3sec - >10 trials per condition - **Endpoint: significant changes in VGS metrics**   **AS error rate [54, 77]**   - Duration of center target: 150msec - Horizontal target (±13°, ±25°) - Instruction to look in opposite hemifield as quickly as possible - Inter-step interval: 1sec - >10 trials per condition - **Endpoint: significant changes in AS error rate** | **Presence of GEN [54]**   - Same parameters as for SCA1 - **Endpoint: significant changes in SPV GEN** | None | None | - No longitudinal data - No treatment trials - No correlation analyses available |
| **AOA2** | **VGS dysmetria [54, 78-80]**   - Horizontal saccades (random order, from center to target) - Amplitude: ±10°, ±18° and ±25° - Duration of center target: 0.5-1sec - Inter-step interval: 1sec - >10 trials per condition - **Endpoint: significant changes in VGS metrics**   **PV horizontal VGS [54, 78, 79])**   - Same parameters as for VGS dysmetria - **Endpoint: significant changes in horizontal VGS peak velocity**   **AS error rate [54, 78-80]**   - Duration of center target: 150msec - Horizontal target (±13°, ±25°) - Instruction to look in opposite hemifield as quickly as possible - Inter-step interval: 1sec - >10 trials per condition - **Endpoint: significant changes in AS error rate** | **Presence of GEN [54]**   - Same parameters as for SCA1 - **Endpoint: significant changes in SPV GEN** | None | None | - No longitudinal data - No treatment trials - No correlation analyses available |
| **FXTAS** | **AS latency [81-84] and error rate [81, 82, 84]**   - Duration of center target: 1.4-2.4sec - Horizontal target (±5°, ±20°) - Instruction to look in opposite hemifield as quickly as possible - >10 trials per condition - **Endpoint: significant changes in AS error rate and latency** | None | None | None | - Most data on SEM - Very limited (PEM) or lacking data for other OM domains (SI, SN, GEN, qHIT). - No longitudinal data - No treatment trials |
| **CTX** | **Horizontal VGS metrics [85, 86]**   - Horizontal saccades (random order, from center to target) - Amplitude: ±10°, ±18° and ±40° - Duration of center target: 1.5-2.5sec - Inter-step interval: 1.5sec - >10 trials per condition - **Endpoint: significant changes in VGS metrics**   **VGS latency [85])**   - Same parameters as for VGS metrics (CTX) - **Endpoint: significant changes in VGS latency**   **AS latency [85] and error rate [85]**   - Duration of center target: 1.5-2.5sec - Horizontal target (±10°, ±18°) - Instruction to look in opposite hemifield as quickly as possible - Inter-step interval: 2.5sec - >10 trials per condition - **Endpoints: significant changes in AS error rate and/or AS latency** | None | None | None | - Limited data, only two publications (with a total of 23 patients) identified and only two OM domains studied. No correlation analyses. - No longitudinal data - No treatment trials |

* For all paradigms comparison with age-matched healthy controls is recommended (including statistical analysis), for longitudinal (observational) studies comparison with baseline is recommended to monitor disease progression. For treatment trials both comparison pre-/post treatment and to placebo group is recommended. Minimal recommended recording frequency for VOG is 200Hz. Whenever specific parameters (e.g. inter-step interval or center target on) were lacking for a given disease, consensus generic parameters proposed by Garces et al. 2023 [87] were used. For some diseases and OM domains, more than one paradigm has been proposed (e.g. VGS latency or AS latency for FRDA). Depending on the specific research question, selected paradigms may be used only.

**Abbreviations**: 4-AP=4-aminopyridine; AOA=ataxia with oculomotor apraxia; AS=anti-saccades; A-T=ataxia telangiectasia; aVOR=angular vestibulo-ocular reflex; CTX=cerebrotendinous xanthomatosis; DBN=downbeat nystagmus; EA2=episodic ataxia type 2; FRDA=Friedreich Ataxia; fXPCs=fragile X premutation carriers; FXTAS=fragile-X associated tremor/ataxia syndrome; GEN=gaze-evoked nystagmus; HC=horizontal canal; Hor=horizontal; IQR=inter-quartile range; MGS=memory-guided saccades; MRI=magnetic resonance imaging; NPC=Niemann-Pick disease Type C; OM=oculomotor; PEM=pursuit eye movements; PV=peak velocity; qHIT=quantitative head-impulse test; RFC1=replication factor C subunit 1; SARA=Scale for the Assessment and Rating of Ataxia; SCA=spinocerebellar ataxia; SEM=saccadic eye movements; SI=saccadic intrusions; SN=spontaneous nystagmus; SPV=slow phase velocity; SWJ=square-wave jerks; Tc=time constant; tx=treatment; VC=vertical canal; VGS=visually-guided saccades; vHIT=video-head-impulse test.

**Table A6-2: Proposed disease-specific oculomotor paradigms (from up to 4 OM domains) stratified for the specific research question**

| **Disease** | **Disease characterization** | **Natural course of disease** | **Treatment response** | **Pre-ataxic carriers** |
| --- | --- | --- | --- | --- |
| **FRDA** | **VGS latency [1-7]:**   - See table A6-1 (FRDA) for parameters - **Endpoint: significant increase in VGS latency**   **AS latency [3]:**   - See table A6-1 (FRDA) for parameters - **Endpoint: significant increase in AS latency** | No longitudinal studies identified | **Presence of SWJ** [13], but no significant treatment effect of study drug (idebenone) observed | No studies with pre-symptomatic carriers identified |
| **SCA1** | **PV VGS [1, 15-18]**   - See table A6-1 (SCA1) for parameters - **Endpoint: significant reduction in VGS peak velocity**   **Presence of SWJ [1, 17, 19]**   - See table A6-1 (FRDA) for parameters - **Endpoints: significant increase in frequency and/or amplitude of SWJ**   **Presence of GEN [17, 19]**   - See table A6-1 (SCA1) for parameters - **Endpoint: significant increase in SPV GEN** | No longitudinal studies identified | No treatment studies identified | No studies with pre-symptomatic carriers identified |
| **SCA2** | **PV VGS [15-17, 20-30]**   - See table A6-1 (SCA2) for parameters - **Endpoint: significant reduction in VGS peak velocity** | **PV, latency and accuracy of VGS [24, 26]**   - See table A6-1 (SCA2) for parameters - **Endpoints: significant reduction in VGS peak velocity, increase in VGS latency and/or decrease in VGS accuracy over 5 years** | **Latency of VGS [28, 29]**   - See table A6-1 (SCA2) for parameters - **Endpoints: significant reduction in VGS latency under treatment with zinc sulfate [28] or human recombinant EPO [29]** | **PV VGS ([22, 23])**   - See table A6-1 (SCA2) for parameters - **Endpoint: significant reduction in VGS peak velocity in pre-ataxic carriers** |
| **SCA3** | **PV VGS [30-35]**   - See table A6-1 (SCA3) for parameters - **Endpoint: significant reduction in vertical VGS peak velocity**   **Presence of GEN [17, 19, 30, 32-34]**   - See table A6-1 (SCA1) for parameters - **Endpoint: significant increase in SPV GEN**   **HC and VC vHIT gains [30, 32, 36-39]**   - See table A6-1 (SCA3) for parameters - **Endpoint: significant reduction in HC vHIT gains** | **HC vHIT gains [36]**   - See table A6-1 (SCA3) for parameters - **Endpoint: significant reduction in HC vHIT gains over 9-24 months** | No treatment studies identified | **PV VGS ([32, 35])**   - See table A6-1 (SCA3) for parameters - **Endpoint: significant reduction in vertical VGS peak velocity** **in pre-ataxic carriers**   **Pursuit gain ([32, 35])**   - Vertical pursuit eye movements - Constant velocity (10°/sec) moving target between ±10° eccentricity. - **Endpoint: significant reduction in PEM gain in pre-ataxic carriers**   **Presence of GEN [32]**   - See table A6-1 (SCA1) for parameters - **Endpoint: significant increase in SPV GEN in pre-ataxic carriers**   **HC vHIT gains [32]**   - See table A6-1 (SCA3) for parameters - **Endpoint: significant reduction in HC vHIT gains in pre-ataxic carriers** |
| **SCA6** | **VGS dysmetria [17, 19, 40-42]**   - See table A6-1 (SCA6) for parameters - **Endpoint: significant hypometria or hypermetria of VGS**   **Pursuit gain [17, 18, 30, 40-45]**   - See table A6-1 (SCA6) for parameters - **Endpoint: significant reduction in PEM gain**   **Presence of DBN [17, 19, 30, 42, 43, 46]**   - See table A6-1 (SCA6) for parameters - **Endpoint: significant increase in SPV DBN**   **HC and VC vHIT gains [30, 42, 47]**   - See table A6-1 (SCA3) for parameters - **Endpoint: significant reduction in HC and/or VC vHIT gains** | **HC and AC vHIT gains [42]**   - See table A6-1 (SCA6) for parameters - **Endpoint: significant reduction in HC and AC vHIT gains over 3-60 months** | No treatment studies identified | **VGS dysmetria ([40])**   - See table A6-1 (SCA6) for parameters - **Endpoint: significant hypometria or hypermetria of VGS in pre-ataxic carriers**   **Pursuit gain ([40])**   - See table A6-1 (SCA6) for parameters - **Endpoint: significant reduction in PEM gain in pre-ataxic carriers** |
| **SCA7** | **PV VGS [30, 48]**   - See table A6-1 (SCA7) for parameters - **Endpoint: significant reduction in VGS peak velocity**   **Pursuit gain [30, 48]**   - See table A6-1 (SCA7) for parameters - **Endpoint: significant reduction in PEM gain**   **HC and VC vHIT gains [30, 48]**   - See table A6-1 (SCA7) for parameters - **Endpoint: significant reduction in HC and/or VC vHIT gains** | No longitudinal studies identified | No treatment studies identified | No studies with pre-symptomatic carriers identified |
| **EA2** | **Pursuit gain [20, 45, 49-51]**   - See table A6-1 (SCA6) for parameters - **Endpoint: significant changes in PEM gain**   **HC and VC vHIT gains[30, 42, 47] [49, 50]**   - See table A6-1 (SCA3) for parameters - **Endpoint: significant changes in HC and/or VC vHIT gains**   **Presence of GEN [49, 51]**   - See table A6-1 (SCA1) for parameters - **Endpoint: significant changes in SPV GEN** | No longitudinal studies identified | No treatment studies identified | No studies with pre-symptomatic carriers identified |
| **A-T** | **VGS hypometria [52-54]**   - See table A6-1 (SCA6) for parameters - **Endpoint: significant hypometria of VGS**   **Pursuit gain [52-55]**   - See table A6-1 (SCA6) for parameters - **Endpoint: significant reduction in PEM gain**   **Presence of DBN [54, 56-58]**   - See table A6-1 (SCA6) for parameters - **Endpoint: significant increase in SPV DBN**   **Presence of SWJ**  **[53, 54, 56]**   - See table A6-1 (FRDA) for parameters - **Endpoint: significant increase in frequency and/or amplitude of SWJ** | No longitudinal studies identified | **Presence of DBN [57, 58]**   - See table A6-1 (SCA6) for parameters - **Endpoint: significant reduction in SPV DBN under treatment with acetyl-DL-leucine [58] or 4-AP [57]** | No studies with pre-symptomatic carriers identified |
| **NPC** | **PV VGS [59-64]**   - See table A6-1 (SCA3) for parameters - **Endpoint: significant reduction in vertical VGS peak velocity** | No longitudinal studies identified | **PV VGS [65-70]**   - See table A6-1 (SCA3) for parameters - **Endpoint: significant improvement in horizontal VGS peak velocity and/or gain** | No studies with pre-symptomatic carriers identified |
| **RFC1-related disease** | **HC and VC vHIT gains [71-74]**   - See table A6-1 (SCA3) for parameters - **Endpoint: significant reduction in HC vHIT gains**   **Presence of DBN [71]**   - See table A6-1 (A-T) for parameters - **Endpoint: significant increase in SPV DBN** | No longitudinal studies identified | No treatment studies identified | No studies with pre-symptomatic carriers identified |
| **AOA1** | **VGS dysmetria [54, 77]**   - See table A6-1 (AOA1) for parameters - **Endpoint: significant hypometria or hypermetria of VGS**   **AS error rate [54, 77]**   - See table A6-1 (AOA1) for parameters - **Endpoint: significant increase in AS error rate** | No longitudinal studies identified | No treatment studies identified | No studies with pre-symptomatic carriers identified |
| **AOA2** | **VGS dysmetria [54, 78-80]**   - See table A6-1 (AOA2) for parameters - **Endpoint: significant hypometria or hypermetria of VGS**   **PV horizontal VGS [54, 78, 79])**   - Same parameters as for VGS dysmetria - **Endpoint: significant reduction in horizontal VGS peak velocity**   **AS error rate [54, 78-80]**   - See table A6-1 (AOA2) for parameters - **Endpoint: significant increase in AS error rate** | No longitudinal studies identified | No treatment studies identified | No studies with pre-symptomatic carriers identified |
| **FXTAS** | **AS latency [81-84] and error rate [81, 82, 84]**   - See table A6-1 (FXTAS) for parameters - **Endpoint: significant increase in AS error rate and in latency** | No longitudinal studies identified | No treatment studies identified | **AS latency [81-84] and error rate [81, 82, 84]**   - See table A6-1 (FXTAS) for parameters - **Endpoint: significant increase in AS error rate and in latency in pre-ataxic carriers** |
| **CTX** | **Horizontal VGS metrics [85, 86]**   - See table A6-1 (AOA2) for parameters - **Endpoint: significant hypometria or hypermetria of VGS**   **VGS latency [85])**   - Same parameters as for VGS metrics (CTX) - **Endpoint: significant increase in VGS latency**   **AS latency [85] and error rate [85]**   - See table A6-1 (AOA2) for parameters - **Endpoints: significant increase in AS error rate and/or significant increase in AS latency** | No longitudinal studies identified | No treatment studies identified | No studies with pre-symptomatic carriers identified |
| **GAA-FGF14-related ataxia** | **Presence of DBN [75, 76]**   - See table A6-1 (SCA6) for parameters - **Endpoint: significant increase in SPV DBN**   **Bilateral HC vHIT gain [75]**   - See table A6-1 (SCA3) for parameters - **Endpoint: significant reduction in HC vHIT gains** | No longitudinal studies identified | **Presence of DBN [75]**   - See table A6-1 (SCA6) for parameters - **Endpoint: significant reduction in SPV DBN under treatment with 4-AP [75]** | No studies with pre-symptomatic carriers identified |

* For all paradigms comparison with age-matched healthy controls is recommended (including statistical analysis), for longitudinal (observational) studies comparison with baseline is recommended to monitor disease progression. For treatment trials both comparison pre-/post treatment and to placebo group is recommended. Minimal recommended recording frequency for VOG is 200Hz.

Abbreviations: 4-AP=4-aminopyridine; AOA=ataxia with oculomotor apraxia; AS=anti-saccades; A-T=ataxia telangiectasia; aVOR=angular vestibulo-ocular reflex; CTX=cerebrotendinous xanthomatosis; DBN=downbeat nystagmus; EA2=episodic ataxia type 2; FRDA=Friedreich Ataxia; fXPCs=fragile X premutation carriers; FXTAS=fragile-X associated tremor/ataxia syndrome; GEN=gaze-evoked nystagmus; HC=horizontal canal; Hor=horizontal; MGS=memory-guided saccades; MRI=magnetic resonance imaging; NPC=Niemann-Pick disease Type C; OM=oculomotor; PEM=pursuit eye movements; PV=peak velocity; qHIT=quantitative head-impulse test; RFC1=replication factor C subunit 1; SCA=spinocerebellar ataxia; SEM=saccadic eye movements; SI=saccadic intrusions; SN=spontaneous nystagmus; SPV=slow phase velocity; SWJ=square-wave jerks; Tc=time constant; tx=treatment; VGS=visually-guided saccades; vHIT=video-head-impulse test.

**References**

[1] Alexandre MF, Rivaud-Péchoux S, Challe G, Durr A and Gaymard B. Functional consequences of oculomotor disorders in hereditary cerebellar ataxias. Cerebellum (London, England) 2013: 12:396-405. doi 10.1007/s12311-012-0433-z

[2] Fahey MC, Cremer PD, Aw ST, Millist L, Todd MJ, White OB, Halmagyi M, Corben LA, Collins V, Churchyard AJ, Tan K, Kowal L and Delatycki MB. Vestibular, saccadic and fixation abnormalities in genetically confirmed Friedreich ataxia. Brain : a journal of neurology 2008: 131:1035-45. doi 10.1093/brain/awm323

[3] Fielding J, Corben L, Cremer P, Millist L, White O and Delatycki M. Disruption to higher order processes in Friedreich ataxia. Neuropsychologia 2010: 48:235-42. doi 10.1016/j.neuropsychologia.2009.09.009

[4] Hocking DR, Corben LA, Fielding J, Cremer PD, Millist L, White OB and Delatycki MB. Saccade reprogramming in Friedreich ataxia reveals impairments in the cognitive control of saccadic eye movement. Brain and cognition 2014: 87:161-7. doi 10.1016/j.bandc.2014.03.018

[5] Wessel K, Moschner C, Wandinger KP, Kömpf D and Heide W. Oculomotor testing in the differential diagnosis of degenerative ataxic disorders. Archives of neurology 1998: 55:949-56. doi 10.1001/archneur.55.7.949

[6] Moschner C, Perlman S and Baloh RW. Comparison of oculomotor findings in the progressive ataxia syndromes. Brain : a journal of neurology 1994: 117 ( Pt 1):15-25. doi 10.1093/brain/117.1.15

[7] Hocking DR, Fielding J, Corben LA, Cremer PD, Millist L, White OB and Delatycki MB. Ocular motor fixation deficits in Friedreich ataxia. Cerebellum (London, England) 2010: 9:411-8. doi 10.1007/s12311-010-0178-5

[8] Baloh RW, Konrad HR and Honrubia V. Vestibulo-ocular function in patients with cerebellar atrophy. Neurology 1975: 25:160-8. doi 10.1212/wnl.25.2.160

[9] Ciuffreda KJ, Kenyon RV and Stark L. Eye movements during reading: further case reports. American journal of optometry and physiological optics 1985: 62:844-52. doi 10.1097/00006324-198512000-00005

[10] Dale RT, Kirby AW and Jampel RS. Square wave jerks in Friedreich's ataxia. American journal of ophthalmology 1978: 85:400-6. doi 10.1016/s0002-9394(14)77738-4

[11] Ell J, Prasher D and Rudge P. Neuro-otological abnormalities in Friedreich's ataxia. Journal of neurology, neurosurgery, and psychiatry 1984: 47:26-32. doi 10.1136/jnnp.47.1.26

[12] Furman JM, Perlman S and Baloh RW. Eye movements in Friedreich's ataxia. Archives of neurology 1983: 40:343-6. doi 10.1001/archneur.1983.04050060043006

[13] Ribaï P, Pousset F, Tanguy ML, Rivaud-Pechoux S, Le Ber I, Gasparini F, Charles P, Béraud AS, Schmitt M, Koenig M, Mallet A, Brice A and Dürr A. Neurological, cardiological, and oculomotor progression in 104 patients with Friedreich ataxia during long-term follow-up. Archives of neurology 2007: 64:558-64. doi 10.1001/archneur.64.4.558

[14] Spieker S, Schulz JB, Petersen D, Fetter M, Klockgether T and Dichgans J. Fixation instability and oculomotor abnormalities in Friedreich's ataxia. Journal of neurology 1995: 242:517-21. doi 10.1007/bf00867423

[15] Bürk K, Fetter M, Skalej M, Laccone F, Stevanin G, Dichgans J and Klockgether T. Saccade velocity in idiopathic and autosomal dominant cerebellar ataxia. Journal of neurology, neurosurgery, and psychiatry 1997: 62:662-4. doi 10.1136/jnnp.62.6.662

[16] Bürk K, Abele M, Fetter M, Dichgans J, Skalej M, Laccone F, Didierjean O, Brice A and Klockgether T. Autosomal dominant cerebellar ataxia type I clinical features and MRI in families with SCA1, SCA2 and SCA3. Brain : a journal of neurology 1996: 119 ( Pt 5):1497-505. doi 10.1093/brain/119.5.1497

[17] Buttner N, Geschwind D, Jen JC, Perlman S, Pulst SM and Baloh RW. Oculomotor phenotypes in autosomal dominant ataxias. Archives of neurology 1998: 55:1353-7. doi 10.1001/archneur.55.10.1353

[18] Kerber KA, Jen JC, Perlman S and Baloh RW. Late-onset pure cerebellar ataxia: differentiating those with and without identifiable mutations. Journal of the neurological sciences 2005: 238:41-5. doi 10.1016/j.jns.2005.06.006

[19] Kim JS, Kim JS, Youn J, Seo DW, Jeong Y, Kang JH, Park JH and Cho JW. Ocular motor characteristics of different subtypes of spinocerebellar ataxia: distinguishing features. Movement disorders : official journal of the Movement Disorder Society 2013: 28:1271-7. doi 10.1002/mds.25464

[20] Anderson JH, Christova PS, Xie TD, Schott KS, Ward K and Gomez CM. Spinocerebellar ataxia in monozygotic twins. Archives of neurology 2002: 59:1945-51. doi 10.1001/archneur.59.12.1945

[21] Federighi P, Cevenini G, Dotti MT, Rosini F, Pretegiani E, Federico A and Rufa A. Differences in saccade dynamics between spinocerebellar ataxia 2 and late-onset cerebellar ataxias. Brain : a journal of neurology 2011: 134:879-91. doi 10.1093/brain/awr009

[22] Reetz K, Rodríguez-Labrada R, Dogan I, Mirzazade S, Romanzetti S, Schulz JB, Cruz-Rivas EM, Alvarez-Cuesta JA, Aguilera Rodríguez R, Gonzalez Zaldivar Y, Auburger G and Velázquez-Pérez L. Brain atrophy measures in preclinical and manifest spinocerebellar ataxia type 2. Annals of clinical and translational neurology 2018: 5:128-37. doi 10.1002/acn3.504

[23] Rodríguez-Labrada R, Vázquez-Mojena Y, Canales-Ochoa N, Medrano-Montero J and Velázquez-Pérez L. Heritability of saccadic eye movements in spinocerebellar ataxia type 2: insights into an endophenotype marker. Cerebellum & ataxias 2017: 4:19. doi 10.1186/s40673-017-0078-2

[24] Rodríguez-Labrada R, Velázquez-Pérez L, Auburger G, Ziemann U, Canales-Ochoa N, Medrano-Montero J, Vázquez-Mojena Y and González-Zaldivar Y. Spinocerebellar ataxia type 2: Measures of saccade changes improve power for clinical trials. Movement disorders : official journal of the Movement Disorder Society 2016: 31:570-8. doi 10.1002/mds.26532

[25] Rufa A and Federighi P. Fast versus slow: different saccadic behavior in cerebellar ataxias. Annals of the New York Academy of Sciences 2011: 1233:148-54. doi 10.1111/j.1749-6632.2011.06126.x

[26] Seifried C, Velázquez-Pérez L, Santos-Falcón N, Abele M, Ziemann U, Almaguer LE, Martínez-Góngora E, Sánchez-Cruz G, Canales N, Pérez-González R, Velázquez-Manresa M, Viebahn B, Stuckrad-Barre S, Klockgether T, Fetter M and Auburger G. Saccade velocity as a surrogate disease marker in spinocerebellar ataxia type 2. Annals of the New York Academy of Sciences 2005: 1039:524-7. doi 10.1196/annals.1325.059

[27] Velázquez-Pérez L, Rodríguez-Labrada R, Álvarez-González L, Aguilera-Rodríguez R, Álvarez Sánchez M, Canales-Ochoa N, Galicia Polo L, Haro-Valencia R, Medrano-Montero J, Vázquez-Mojena Y, Peña-Acosta A, Estupiñán-Rodríguez A and Rodríguez Pupo N. Lisuride reduces involuntary periodic leg movements in spinocerebellar ataxia type 2 patients. Cerebellum (London, England) 2012: 11:1051-6. doi 10.1007/s12311-012-0382-6

[28] Velázquez-Pérez L, Rodríguez-Chanfrau J, García-Rodríguez JC, Sánchez-Cruz G, Aguilera-Rodríguez R, Rodríguez-Labrada R, Rodríguez-Díaz JC, Canales-Ochoa N, Gotay DA, Almaguer Mederos LE, Laffita Mesa JM, Porto-Verdecia M, Triana CG, Pupo NR, Batista IH, López-Hernandez OD, Polanco ID and Novas AJ. Oral zinc sulphate supplementation for six months in SCA2 patients: a randomized, double-blind, placebo-controlled trial. Neurochemical research 2011: 36:1793-800. doi 10.1007/s11064-011-0496-0

[29] Rodriguez-Labrada R, Ortega-Sanchez R, Hernandez Casana P, Santos Morales O, Padron-Estupinan MDC, Batista-Nunez M, Jimenez Rodriguez D, Canales-Ochoa N, Pena Acosta A, Medrano Montero J, Labrada Aguilera PE, Estupinan Rodriguez A, Vazquez-Mojena Y, Almaguer Gotay D, Aymed-Garcia J, Garcia-Garcia I, Torres Vega R, Viada Gonzalez C, Valenzuela Silva CM, Silva Ricardo Y, Columbie Ximelis J, Tribin Rivero K, Valle Cabrera R, Garcia-Rodriguez JC, Crombet Ramos T, Amaro-Gonzalez D, Rodriguez-Obaya T and Velazquez-Perez L. Erythropoietin in Spinocerebellar Ataxia Type 2: Feasibility and Proof-of-Principle Issues from a Randomized Controlled Study. Movement disorders : official journal of the Movement Disorder Society 2022: 37:1516-25. doi 10.1002/mds.29045

[30] Kim JM, Nam TS, Choi SM, Kim BC and Lee SH. Clinical value of vestibulo-ocular reflex in the differentiation of spinocerebellar ataxias. Scientific reports 2023: 13:14783. doi 10.1038/s41598-023-41924-6

[31] Caspi A, Zivotofsky AZ and Gordon CR. Multiple saccadic abnormalities in spinocerebellar ataxia type 3 can be linked to a single deficiency in velocity feedback. Investigative ophthalmology & visual science 2013: 54:731-8. doi 10.1167/iovs.12-10689

[32] de Oliveira CM, Leotti VB, Bolzan G, Cappelli AH, Rocha AG, Ecco G, Kersting N, Rieck M, Martins AC, Sena LS, Saraiva-Pereira ML and Jardim LB. Pre-ataxic Changes of Clinical Scales and Eye Movement in Machado-Joseph Disease: BIGPRO Study. Movement disorders : official journal of the Movement Disorder Society 2021. doi 10.1002/mds.28466

[33] Ghasia FF, Wilmot G, Ahmed A and Shaikh AG. Strabismus and Micro-Opsoclonus in Machado-Joseph Disease. Cerebellum (London, England) 2016: 15:491-7. doi 10.1007/s12311-015-0718-0

[34] Lemos J, Novo A, Duque C, Castelhano J, Eggenberger E and Januário C. "Pinball" intrusions in spinocerebellar ataxia type 3. Neurology 2018: 90:36-7. doi 10.1212/wnl.0000000000004772

[35] Wu C, Chen DB, Feng L, Zhou XX, Zhang JW, You HJ, Liang XL, Pei Z and Li XH. Oculomotor deficits in spinocerebellar ataxia type 3: Potential biomarkers of preclinical detection and disease progression. CNS Neurosci Ther 2017: 23:321-8. doi 10.1111/cns.12676

[36] Elyoseph Z, Geisinger D, Zaltzman R, Mintz M and Gordon CR. Horizontal Vestibulo-Ocular Reflex Deficit as a Biomarker for Clinical Disease Onset, Severity, and Progression of Machado-Joseph Disease. Cerebellum (London, England) 2023. doi 10.1007/s12311-023-01552-2

[37] Luis L, Costa J, Munoz E, de Carvalho M, Carmona S, Schneider E, Gordon CR and Valls-Sole J. Vestibulo-ocular reflex dynamics with head-impulses discriminates spinocerebellar ataxias types 1, 2 and 3 and Friedreich ataxia. Journal of vestibular research : equilibrium & orientation 2016: 26:327-34. doi 10.3233/VES-160579

[38] Geisinger D, Elyoseph Z, Zaltzman R, Mintz M and Gordon CR. Angular vestibulo ocular reflex loss with preserved saccular function in Machado-Joseph disease. Journal of the neurological sciences 2021: 424:117393. doi 10.1016/j.jns.2021.117393

[39] Gordon CR, Zivotofsky AZ and Caspi A. Impaired vestibulo-ocular reflex (VOR) in spinocerebellar ataxia type 3 (SCA3): bedside and search coil evaluation. Journal of vestibular research : equilibrium & orientation 2014: 24:351-5. doi 10.3233/ves-140527

[40] Christova P, Anderson JH and Gomez CM. Impaired eye movements in presymptomatic spinocerebellar ataxia type 6. Archives of neurology 2008: 65:530-6. doi 10.1001/archneur.65.4.530

[41] Hashimoto T, Sasaki O, Yoshida K, Takei Y and Ikeda S. Periodic alternating nystagmus and rebound nystagmus in spinocerebellar ataxia type 6. Movement disorders : official journal of the Movement Disorder Society 2003: 18:1201-4. doi 10.1002/mds.10511

[42] Lee SU, Kim JS, Kim HJ, Choi JY, Park JY, Kim JM and Yang X. Evolution of the vestibular function during head impulses in spinocerebellar ataxia type 6. Journal of neurology 2020: 267:1672-8. doi 10.1007/s00415-020-09756-w

[43] Bour LJ, van Rootselaar AF, Koelman JH and Tijssen MA. Oculomotor abnormalities in myoclonic tremor: a comparison with spinocerebellar ataxia type 6. Brain : a journal of neurology 2008: 131:2295-303. doi 10.1093/brain/awn177

[44] Takeichi N, Fukushima K, Sasaki H, Yabe I, Tashiro K and Inuyama Y. Dissociation of smooth pursuit and vestibulo-ocular reflex cancellation in SCA-6. Neurology 2000: 54:860-6. doi 10.1212/wnl.54.4.860

[45] Wiest G, Tian JR, Baloh RW, Crane BT and Demer JL. Otolith function in cerebellar ataxia due to mutations in the calcium channel gene CACNA1A. Brain : a journal of neurology 2001: 124:2407-16. doi 10.1093/brain/124.12.2407

[46] Gomez CM, Thompson RM, Gammack JT, Perlman SL, Dobyns WB, Truwit CL, Zee DS, Clark HB and Anderson JH. Spinocerebellar ataxia type 6: gaze-evoked and vertical nystagmus, Purkinje cell degeneration, and variable age of onset. Annals of neurology 1997: 42:933-50. doi 10.1002/ana.410420616

[47] Huh YE, Kim JS, Kim HJ, Park SH, Jeon BS, Kim JM, Cho JW and Zee DS. Vestibular Performance During High-Acceleration Stimuli Correlates with Clinical Decline in SCA6. Cerebellum (London, England) 2015: 14:284-91. doi 10.1007/s12311-015-0650-3

[48] Oh AK, Jacobson KM, Jen JC and Baloh RW. Slowing of voluntary and involuntary saccades: an early sign in spinocerebellar ataxia type 7. Annals of neurology 2001: 49:801-4. doi 10.1002/ana.1059

[49] Choi JH, Oh EH, Choi SY, Kim HJ, Lee SK, Choi JY, Kim JS and Choi KD. Vestibular impairments in episodic ataxia type 2. Journal of neurology 2022: 269:2687-95. doi 10.1007/s00415-021-10856-4

[50] Gordon CR, Caspi A, Levite R and Zivotofsky AZ. Mechanisms of vestibulo-ocular reflex (VOR) cancellation in spinocerebellar ataxia type 3 (SCA-3) and episodic ataxia type 2 (EA-2). Prog Brain Res 2008: 171:519-25. doi 10.1016/S0079-6123(08)00674-2

[51] Baloh RW, Yue Q, Furman JM and Nelson SF. Familial episodic ataxia: clinical heterogeneity in four families linked to chromosome 19p. Annals of neurology 1997: 41:8-16. doi 10.1002/ana.410410105

[52] Lewis RF and Crawford TO. Slow target-directed eye movements in ataxia-telangiectasia. Investigative ophthalmology & visual science 2002: 43:686-91.

[53] Lewis RF, Lederman HM and Crawford TO. Ocular motor abnormalities in ataxia telangiectasia. Annals of neurology 1999: 46:287-95. doi 10.1002/1531-8249(199909)46:3<287::aid-ana3>3.0.co;2-0

[54] Mariani LL, Rivaud-Pechoux S, Charles P, Ewenczyk C, Meneret A, Monga BB, Fleury MC, Hainque E, Maisonobe T, Degos B, Echaniz-Laguna A, Renaud M, Wirth T, Grabli D, Brice A, Vidailhet M, Stoppa-Lyonnet D, Dubois-d'Enghien C, Le Ber I, Koenig M, Roze E, Tranchant C, Durr A, Gaymard B and Anheim M. Comparing ataxias with oculomotor apraxia: a multimodal study of AOA1, AOA2 and AT focusing on video-oculography and alpha-fetoprotein. Scientific reports 2017: 7:15284. doi 10.1038/s41598-017-15127-9

[55] Baloh RW, Yee RD and Boder E. Eye movements in ataxia-telangiectasia. Neurology 1978: 28:1099-104. doi 10.1212/wnl.28.11.1099

[56] Shaikh AG, Marti S, Tarnutzer AA, Palla A, Crawford TO, Straumann D, Taylor AM and Zee DS. Gaze fixation deficits and their implication in ataxia-telangiectasia. Journal of neurology, neurosurgery, and psychiatry 2009: 80:858-64. doi 10.1136/jnnp.2008.170522

[57] Shaikh AG, Marti S, Tarnutzer AA, Palla A, Crawford TO, Zee DS and Straumann D. Effects of 4-aminopyridine on nystagmus and vestibulo-ocular reflex in ataxia-telangiectasia. Journal of neurology 2013: 260:2728-35. doi 10.1007/s00415-013-7046-4

[58] Brueggemann A, Bicvic A, Goeldlin M, Kalla R, Kerkeni H, Mantokoudis G, Abegg M, Kolnikova M, Mohaupt M and Bremova-Ertl T. Effects of Acetyl-DL-Leucine on Ataxia and Downbeat-Nystagmus in Six Patients With Ataxia Telangiectasia. J Child Neurol 2022: 37:20-7. doi 10.1177/08830738211028394

[59] Solomon D, Winkelman AC, Zee DS, Gray L and Büttner-Ennever J. Niemann-Pick type C disease in two affected sisters: ocular motor recordings and brain-stem neuropathology. Annals of the New York Academy of Sciences 2005: 1039:436-45. doi 10.1196/annals.1325.041

[60] Havla J, Moser M, Sztatecsny C, Lotz-Havla AS, Maier EM, Hizli B, Schinner R, Kümpfel T, Strupp M, Bremova-Ertl T and Schneider SA. Retinal axonal degeneration in Niemann-Pick type C disease. Journal of neurology 2020: 267:2070-82. doi 10.1007/s00415-020-09796-2

[61] Bremova T, Malinova V, Amraoui Y, Mengel E, Reinke J, Kolnikova M and Strupp M. Acetyl-dl-leucine in Niemann-Pick type C: A case series. Neurology 2015: 85:1368-75. doi 10.1212/WNL.0000000000002041

[62] Rottach KG, von Maydell RD, Das VE, Zivotofsky AZ, Discenna AO, Gordon JL, Landis DM and Leigh RJ. Evidence for independent feedback control of horizontal and vertical saccades from Niemann-Pick type C disease. Vision Res 1997: 37:3627-38. doi 10.1016/S0042-6989(96)00066-1

[63] Karaaslan Z, Hanagasi HA, Gurvit IH and Bilgic B. Video-Oculography Assessment in Neurodegenerative Ataxias and Niemann Pick Type C. Noro Psikiyatr Ars 2024: 61:101-6. doi 10.29399/npa.28563

[64] Bremova-Ertl T, Abel L, Walterfang M, Salsano E, Ardissone A, Malinova V, Kolnikova M, Gascon Bayarri J, Reza Tavasoli A, Reza Ashrafi M, Amraoui Y, Mengel E, Kolb SA, Brecht A, Bardins S and Strupp M. A cross-sectional, prospective ocular motor study in 72 patients with Niemann-Pick disease type C. European journal of neurology 2021: 28:3040-50. doi 10.1111/ene.14955

[65] Abel LA, Walterfang M, Stainer MJ, Bowman EA and Velakoulis D. Longitudinal assessment of reflexive and volitional saccades in Niemann-Pick Type C disease during treatment with miglustat. Orphanet J Rare Dis 2015: 10:160. doi 10.1186/s13023-015-0377-8

[66] Bowman EA, Walterfang M, Abel L, Desmond P, Fahey M and Velakoulis D. Longitudinal changes in cerebellar and subcortical volumes in adult-onset Niemann-Pick disease type C patients treated with miglustat. Journal of neurology 2015: 262:2106-14. doi 10.1007/s00415-015-7819-z

[67] Patterson MC, Vecchio D, Prady H, Abel L and Wraith JE. Miglustat for treatment of Niemann-Pick C disease: a randomised controlled study. Lancet Neurol 2007: 6:765-72. doi 10.1016/S1474-4422(07)70194-1

[68] Wraith JE, Vecchio D, Jacklin E, Abel L, Chadha-Boreham H, Luzy C, Giorgino R and Patterson MC. Miglustat in adult and juvenile patients with Niemann-Pick disease type C: long-term data from a clinical trial. Molecular genetics and metabolism 2010: 99:351-7. doi 10.1016/j.ymgme.2009.12.006

[69] Zhang H, Xiong H, Wei C, Yi M, Che Y, Zhuo J and Li X. Evaluation of the safety and efficacy of miglustat for the treatment of Chinese patients with Niemann-Pick disease type C: A prospective, open-label, single-arm, phase IV trial. Intractable Rare Dis Res 2024: 13:227-35. doi 10.5582/irdr.2024.01056

[70] Patterson MC, Vecchio D, Jacklin E, Abel L, Chadha-Boreham H, Luzy C, Giorgino R and Wraith JE. Long-term miglustat therapy in children with Niemann-Pick disease type C. J Child Neurol 2010: 25:300-5. doi 10.1177/0883073809344222

[71] Costales M, Casanueva R, Suárez V, Asensi JM, Cifuentes GA, Diñeiro M, Cadiñanos J, López F, Álvarez-Marcos C, Otero A, Gómez J, Llorente JL and Cabanillas R. CANVAS: A New Genetic Entity in the Otorhinolaryngologist's Differential Diagnosis. Otolaryngology--head and neck surgery : official journal of American Academy of Otolaryngology-Head and Neck Surgery 2021:1945998211008398. doi 10.1177/01945998211008398

[72] Borsche M, Tadic V, Konig IR, Lohmann K, Helmchen C and Bruggemann N. Head impulse testing in bilateral vestibulopathy in patients with genetically defined CANVAS. Brain Behav 2022: 12:e32546. doi 10.1002/brb3.2546

[73] Pellerin D, Heindl F, Traschutz A, Rujescu D, Hartmann AM, Brais B, Houlden H, Dufke C, Riess O, Haack T, Strupp M and Synofzik M. RFC1 repeat expansions in downbeat nystagmus syndromes: frequency and phenotypic profile. Journal of neurology 2024: 271:2886-92. doi 10.1007/s00415-024-12229-z

[74] Harrell RG, Cassidy AR, Klatt BN, Hovareshti P and Whitney SL. Vestibular rehabilitation in cerebellar ataxia with neuropathy and vestibular areflexia syndrome (CANVAS)- A case report. J Otol 2023: 18:199-207. doi 10.1016/j.joto.2023.06.004

[75] Pellerin D, Heindl F, Wilke C, Danzi MC, Traschutz A, Ashton C, Dicaire MJ, Cuillerier A, Del Gobbo G, Boycott KM, Claassen J, Rujescu D, Hartmann AM, Zuchner S, Brais B, Strupp M and Synofzik M. GAA-FGF14 disease: defining its frequency, molecular basis, and 4-aminopyridine response in a large downbeat nystagmus cohort. EBioMedicine 2024: 102:105076. doi 10.1016/j.ebiom.2024.105076

[76] Shirai S, Mizushima K, Fujiwara K, Koshimizu E, Matsushima M, Miyatake S, Iwata I, Yaguchi H, Matsumoto N and Yabe I. Case series: Downbeat nystagmus in SCA27B. Journal of the neurological sciences 2023: 454:120849. doi 10.1016/j.jns.2023.120849

[77] Le Ber I, Moreira MC, Rivaud-Pechoux S, Chamayou C, Ochsner F, Kuntzer T, Tardieu M, Said G, Habert MO, Demarquay G, Tannier C, Beis JM, Brice A, Koenig M and Durr A. Cerebellar ataxia with oculomotor apraxia type 1: clinical and genetic studies. Brain : a journal of neurology 2003: 126:2761-72. doi 10.1093/brain/awg283

[78] Bargagli A, Rosini F, Zanca D, Serchi V and Rufa A. Ataxia with oculomotor apraxia type 2 (AOA2): an eye movement study of two siblings. Neurological sciences : official journal of the Italian Neurological Society and of the Italian Society of Clinical Neurophysiology 2021: 42:3039-42. doi 10.1007/s10072-021-05206-1

[79] Le Ber I, Bouslam N, Rivaud-Pechoux S, Guimaraes J, Benomar A, Chamayou C, Goizet C, Moreira MC, Klur S, Yahyaoui M, Agid Y, Koenig M, Stevanin G, Brice A and Durr A. Frequency and phenotypic spectrum of ataxia with oculomotor apraxia 2: a clinical and genetic study in 18 patients. Brain : a journal of neurology 2004: 127:759-67. doi 10.1093/brain/awh080

[80] Panouilleres M, Frismand S, Sillan O, Urquizar C, Vighetto A, Pelisson D and Tilikete C. Saccades and eye-head coordination in ataxia with oculomotor apraxia type 2. Cerebellum (London, England) 2013: 12:557-67. doi 10.1007/s12311-013-0463-1

[81] McLennan YA, Mosconi MW, McKenzie FJ, Famula J, Krawchuk B, Kim K, Clark CJ, Hessl D, Rivera SM, Simon TJ, Tassone F and Hagerman RJ. Prosaccade and Antisaccade Behavior in Fragile X-Associated Tremor/Ataxia Syndrome Progression. Mov Disord Clin Pract 2022: 9:473-8. doi 10.1002/mdc3.13449

[82] Wong LM, Goodrich-Hunsaker NJ, McLennan Y, Tassone F, Zhang M, Rivera SM and Simon TJ. Eye movements reveal impaired inhibitory control in adult male fragile X premutation carriers asymptomatic for FXTAS. Neuropsychology 2014: 28:571-84. doi 10.1037/neu0000066

[83] Lasker AG, Mazzocco MM and Zee DS. Ocular motor indicators of executive dysfunction in fragile X and Turner syndromes. Brain and cognition 2007: 63:203-20. doi 10.1016/j.bandc.2006.08.002

[84] Fielding-Gebhardt H, Kelly SE, Unruh KE, Schmitt LM, Pulver SL, Khemani P and Mosconi MW. Sensorimotor and inhibitory control in aging FMR1 premutation carriers. Front Hum Neurosci 2023: 17:1271158. doi 10.3389/fnhum.2023.1271158

[85] Rosini F, Pretegiani E, Mignarri A, Optican LM, Serchi V, De Stefano N, Battaglini M, Monti L, Dotti MT, Federico A and Rufa A. The role of dentate nuclei in human oculomotor control: insights from cerebrotendinous xanthomatosis. J Physiol 2017: 595:3607-20. doi 10.1113/JP273670

[86] Koens LH, Tuitert I, Blokzijl H, Engelen M, Klouwer FCC, Lange F, Leen WG, Lunsing RJ, Koelman J, Verrips A, de Koning TJ and Tijssen MAJ. Eye movement disorders in inborn errors of metabolism: A quantitative analysis of 37 patients. J Inherit Metab Dis 2022: 45:981-95. doi 10.1002/jimd.12533

[87] Garces P, Antoniades CA, Sobanska A, Kovacs N, Ying SH, Gupta AS, Perlman S, Szmulewicz DJ, Pane C, Nemeth AH, Jardim LB, Coarelli G, Dankova M, Traschutz A and Tarnutzer AA. Quantitative Oculomotor Assessment in Hereditary Ataxia: Systematic Review and Consensus by the Ataxia Global Initiative Working Group on Digital-motor Biomarkers. Cerebellum (London, England) 2023. doi 10.1007/s12311-023-01559-9
