# Supplementary material for: Best Oculomotor Endpoints for Clinical Trials in Hereditary Ataxias: A Systematic Review and Consensus by the Ataxia Global Initiative Working Group on Digital‑Motor Biomarkers
Source: Cerebellum. 2025 Aug 13;24(5):141. doi: 10.1007/s12311-025-01894-z (PMC12350468; doi:10.1007/s12311-025-01894-z)
Supplement: Supplementary file 5 — Supplementary file5 (DOCX 35 KB) [file 12311_2025_1894_MOESM5_ESM.docx]

**Appendix 7: quantitative oculomotor / vestibular findings in hereditary ataxias not meeting minimal number of studies for inclusion in the review (i.e. single study data)**

| **Table A7-1: quantitative oculomotor findings in additional hereditary ataxias** | | | | | | | |
| --- | --- | --- | --- | --- | --- | --- | --- |
|  | **Oculomotor domain** | | | | | | **Comments** |
| **Disease** | **Saccadic eye movements** | **Pursuit eye movements** | **Saccadic intrusions** | **Spontaneous nystagmus** | **Gaze-evoked nystagmus** | **Quantitative head-impulse test** |  |
| **ARSACS [1]** | - Within normal range | - Saccadic PEM | NR | - No SN | - No GEN | - Impaired HC and VC vHIT gains | Data from a single subject only |
| **Ataxia telangiectasia-like disorder type 1 [2]** | - Significantly hypermetric and fast saccades. - Significantly increased mean/peak saccadic velocity (2/2 patients) | NR | - Increased frequency of SI (macro SWJ, macrosaccadic oscillations) (2/2 patients) | - Vertical and horizontal SN observed (2/2 patients) | - GEN observed (2/2 patients) | NR |  |
| **Ataxia with vitamin E deficiency [3]** | NR | NR | - Microsaccadic oscillations at rest (2/2) | NR | NR | NR | VOG data also collected on SEM, PEM, OKN, GEN but no further details provided on findings. |
| **SCA17 [4]** | - VGS with normal latency and velocity, but significantly reduced gain (i.e. hypometric) - AS with significantly increased error rate - MGS: impaired suppression of reflexive saccades | - Significantly reduced PEM velocity | - Rarely SWJ (1/15) | - Vertical SN in few patients (DBN in 1, UBN in 1 patient) | - GEN in some patients (4/15), RBN rarely (1/15). | NR | - PEM gain significantly correlated inversely with disease duration and ICARS score - Saccadic hypometria correlated with disease duration |
| **Spinocerebellar ataxia, autosomal recessive 4 (SCAR4), formerly known as spinocerebellar ataxia with saccadic intrusions (SCASI) [5]** | - Significantly hypermetric horizontal (3/5 patients) and vertical (4/5 patients) VGS. - Significantly increased peak vel for larger saccades | NR | - Macrosaccadic oscillations when shifting gaze and at straight-ahead fixation in 5/5 patients | - No SN | - No GEN observed | NR |  |

Abbreviations: ARSACS=Autosomal recessive spastic ataxia of Charlevoix-Saguenay; AS=anti-saccades; DBN=downbeat nystagmus; GEN=gaze-evoked nystagmus; HC=horizontal canal; Hor=horizontal; ICARS=International Cooperative Ataxia Rating Scale; MGS=memory-guided saccades; NR=not reported; OKN=optokinetic nystagmus; OM=oculomotor; PEM=pursuit eye movements; PV=peak velocity; qHIT=quantitative head-impulse test; RBN=rebound nystagmus; SCA=spinocerebellar ataxia; SCAR4=spinocerebellar ataxia, autosomal recessive 4; SCASI=Spinocerebellar ataxia with saccadic intrusions; SEM=saccadic eye movements; SI=saccadic intrusions; SN=spontaneous nystagmus; SPV=slow phase velocity; SWJ=square-wave jerks; UBN=upbeat nystagmus; VGS=visually-guided saccades; vHIT=video-head-impulse test.

**References**

[1] Argenziano G, Cavallieri F, Castellucci A, Fioravanti V, Di Rauso G, Gessani A, Campanini I, Merlo A, Napoli M, Grisanti S, Rossi J, Toschi G, Zini C, Ghidini A and Valzania F. Vestibular Hypofunction in ARSACS Syndrome: A Possible Pitfall in the Differential Diagnosis of Recessive Cerebellar and Afferent Ataxias. Neurol Clin Pract 2024: 14:e200239. doi 10.1212/CPJ.0000000000200239

[2] Federighi P, Ramat S, Rosini F, Pretegiani E, Federico A and Rufa A. Characteristic Eye Movements in Ataxia-Telangiectasia-Like Disorder: An Explanatory Hypothesis. Frontiers in neurology 2017: 8:596. doi 10.3389/fneur.2017.00596

[3] Koens LH, Tuitert I, Blokzijl H, Engelen M, Klouwer FCC, Lange F, Leen WG, Lunsing RJ, Koelman J, Verrips A, de Koning TJ and Tijssen MAJ. Eye movement disorders in inborn errors of metabolism: A quantitative analysis of 37 patients. J Inherit Metab Dis 2022: 45:981-95. doi 10.1002/jimd.12533

[4] Hübner J, Sprenger A, Klein C, Hagenah J, Rambold H, Zühlke C, Kömpf D, Rolfs A, Kimmig H and Helmchen C. Eye movement abnormalities in spinocerebellar ataxia type 17 (SCA17). Neurology 2007: 69:1160-8. doi 10.1212/01.wnl.0000276958.91986.89

[5] Swartz BE, Li S, Bespalova I, Burmeister M, Dulaney E, Robinson FR and Leigh RJ. Pathogenesis of clinical signs in recessive ataxia with saccadic intrusions. Annals of neurology 2003: 54:824-8. doi 10.1002/ana.10758
